# Synthesis, Anticancer Screening, and In Silico Evaluations of Thieno[2,3-*c*]pyridine Derivatives as Hsp90 Inhibitors

**DOI:** 10.3390/ph18020153

**Published:** 2025-01-24

**Authors:** Balakumar Chandrasekaran, Mohammad F. Bayan, Ali Hmedat, Bilal A. Al-Jaidi, Deniz M. Al-Tawalbeh, Duaa Abuarqoub, Anas J. Rasras, Da’san M. M. Jaradat, Abdel Naser Dakkah, Wafa Hourani, Rajshekhar Karpoormath

**Affiliations:** 1 Department of Pharmaceutical Sciences, Faculty of Pharmacy, Philadelphia University, P.O. Box 1, Amman 19392, Jordan; mbayan@philadelphia.edu.jo (M.F.B.); adokka@philadelphia.edu.jo (A.N.D.); whourani@philadelphia.edu.jo (W.H.); 2 Department of Pharmaceutical Technology and Pharmaceutics, Faculty of Pharmacy, Yarmouk University, Irbid 21163, Jordan; ali.hmedat@yu.edu.jo; 3 Department of Medicinal Chemistry and Pharmacognosy, Faculty of Pharmacy, Yarmouk University, Irbid 21163, Jordan; bilal.aljaidi@yu.edu.jo (B.A.A.-J.); deniz.altawalbeh@yu.edu.jo (D.M.A.-T.); 4 Department of Pharmacology and Medical Sciences, Faculty of Pharmacy and Medical Sciences, University of Petra, Amman 11196, Jordan; duaa.abuarqoub@uop.edu.jo; 5 Department of Chemistry, Faculty of Science, Al-Balqa Applied University, P.O. Box 206, Al-Salt 19117, Jordan; rasras.chem@bau.edu.jo (A.J.R.); dasan.jaradat@bau.edu.jo (D.M.M.J.); 6 Department of Pharmaceutical Chemistry, College of Health Sciences, University of KwaZulu-Natal, Westville Campus, Durban 4000, South Africa; karpoormath@ukzn.ac.za

**Keywords:** Hsp90, anticancer agents, thieno[2,3-*c*]pyridines, cell cycle analysis, molecular docking, ADME prediction

## Abstract

**Background**: Thieno[2,3-*c*]pyridines and their analogs are not well explored for their anticancer properties. Hence, our research aimed to establish the anticancer potential of thieno[2,3-*c*]pyridines through cell-based assays and in silico evaluations. **Methods**: Thieno[2,3-*c*]pyridine derivatives **6(a–k)** were synthesized and characterized using FT-IR, ^1^H-NMR, ^13^C-NMR, and HRMS. All the synthesized compounds were screened initially for their anticancer activity against MCF7 and T47D (breast cancer), HSC3 (head and neck cancer), and RKO (colorectal cancer) cell lines using MTT assay. Apoptosis and cell cycle analyses were conducted using Annexin V/propidium iodide (PI) double staining for apoptosis assessment and PI staining for cell cycle analysis to investigate the mechanisms underlying the reduced cell viability. In silico molecular docking was accomplished for the synthesized compounds against the Hsp90 and determined pharmacokinetics properties. **Results**: From the screening assay, compounds **6a** and **6i** were identified as potential inhibitors and were further subjected to IC_50_ determination. The compound **6i** showed potent inhibition against HSC3 (IC_50_ = 10.8 µM), T47D (IC_50_ = 11.7 µM), and RKO (IC_50_ = 12.4 µM) cell lines, all of which indicated a broad spectrum of anticancer activity. Notably, **6i** was found to induce G2 phase arrest, thereby inhibiting cell cycle progression. Molecular docking results indicated crucial molecular interactions of the synthesized ligands against the target Hsp90. **Conclusion**: The compound **6i** induced cell death via mechanisms that are different from apoptosis. Thus, the synthesized thieno[2,3-*c*]pyridine derivatives can be suitable lead compounds to be optimized to obtain potent anticancer agents through Hsp90 inhibition.

## 1. Introduction

In 2022, 20 million new cancer cases besides 9.7 million cancer-related deaths were recorded worldwide [1]. In addition to the current mortality rate, a 50% rise in cancer cases is anticipated within 15 years from now [2]. Apart from heart disease, cancer is considered to be the second leading cause of death globally [3]. The lung, breast, and colorectal cancers are the three major types of cancer documented by the World Health Organization (WHO) [4]. For cancer treatment, initially palliative therapy was given to assist the patients in obtaining comfort by overcoming their suffering; however, it does not treat the disease [5]. In particular, radiation therapy, immunotherapy, hormone therapy, surgery, and chemotherapy are commonly deputed individually or together to remove or slow down tumor growth [6]. Radiation therapy or radiotherapy utilizes high doses of radiation to shrink or destroy cancer cells [7]. Though radiotherapy focuses on a particular tissue/area of the body, skin changes and fatigue are common side effects experienced by several patients [8]. Immunotherapy of cancer involves the improvement of the immune system of the body to fight against cancer [9], naturally. Modified immunotherapy antibodies are administered which undergo conjugation with tumor antigens thereby unveiling the cancer cells for the immune system to attack specific cancer cells [9]. Besides causing an irregular heartbeat (arrhythmia) or myocarditis, mild-to-severe immune system-related side effects are possible with cancer immunotherapy [10]. Alternatively, hormone therapy was attempted to treat breast and endometrial cancers [11]. Nevertheless, hormone therapy causes long-term side effects such as blood clots, brittle bones, stroke, heart disease, and vision abnormalities [12]. Surgical interventions are usually employed to eliminate cancer-affected tissues which is also considered as palliative care [13]. Post-surgical complications are observed in patients, with the possibility of obtaining an infection, blood clot, or pain [14]. Though chemotherapy is prescribed for the initial treatment of cancer, it may generate toxicity to normal cells over cancer cells [15]. Chemotherapeutic agents are administered alone or in combination to effectively manage different types of cancer [16]. Chemotherapy can be used as the primary or sole cancer therapy or as an adjuvant/neoadjuvant or for palliative care [17]. At present, 90% of failures in chemotherapy are mainly due to drug resistance developed in cancer cells [18]. Thus, drug resistance appears to be a serious issue, besides other common side effects associated with therapeutic agents [19]. To overcome these drawbacks, it is essential to discover new chemotherapeutic agents and pharmaceuticals for an efficient and safe therapy for cancer patients.

Several small organic molecules target key proteins, enzymes, and receptors that are associated with the cell cycle and exhibit anticancer activity [20]. Inhibiting the functions of an ATP-dependent molecular chaperone such as heat shock protein 90 (Hsp90) by small organic molecules proved to be a promising approach in cancer chemotherapy [21]. Hsp90 is a housekeeping gene, with amplified expression of Hsp and heat shock factor-1 (Hsf-1) in cancers, and is related to poor prognosis, metastasis, and chemotherapy resistance [22]. This Hsp90 gene was an established target, and several organic molecules bound to its N-terminal ATP binding site, subsequently affecting the proteasomal degradation of its client proteins [23], consistently. Hsp90 prevails as a homodimer bearing each protomer consisting of an N-terminal nucleotide-binding domain (NTD) along with the enzyme ATPase [24]. A charged linker offers conformational flexibility, whereas, for dimerization, the middle domain (MD), and a carboxy-terminal domain (CTD) contribute equally [25]. The Hsp90 family constitutes four isoforms and is indirectly correlated with the pathophysiology of cancer [26]. Hsp90 is upregulated due to various cellular stresses and alters the stabilization of different client oncoproteins whose functions (folding and maturation) and stability (prevention of aggregation) result in cancer [27]. Higher levels of Hsp90 were observed in breast cancer, colorectal cancer, and head–neck cancers. During the progression of cancer, several transcription factors encoded by proto-oncogenes were stabilized by Hsp90 [22]. To date, there are more than 400 client oncoproteins of Hsp90 that have been identified and characterized. Though Hsp90 functions were regulated, their overexpression led to poor prognosis in cancer [28]. Hsp90 inhibitors cause disturbances in the Hsp90–client protein complex and their interaction, yielding degradation of such client proteins [29]. Hsp90 inhibitors bind to the ATP binding pocket of the N-terminal domain (NTD) leading to the disruption of ATP binding and hydrolysis, finally depleting Hsp90 oncogenic clients [30]. Around 22 compounds were entered into clinical trials and were studied individually and/or in combination with other anticancer agents [31]. Like other chemotherapeutic agents, Hsp90 inhibitors also suffer from drug resistance and toxicity-related issues [32]. Recently, an Hsp90 inhibitor Pimitespib was approved in Japan for the treatment of gastrointestinal stromal tumors and displayed low toxicity [33]. Moreover, this drug was employed as a monotherapy or with nivolumab (an immune checkpoint inhibitor) as a combination therapy [34]. This particular drug has the core structure of a pyrazolo–pyridine moiety [35] which is quite interesting and motivated us to design and develop novel anticancer compounds.

**Figure 1:**
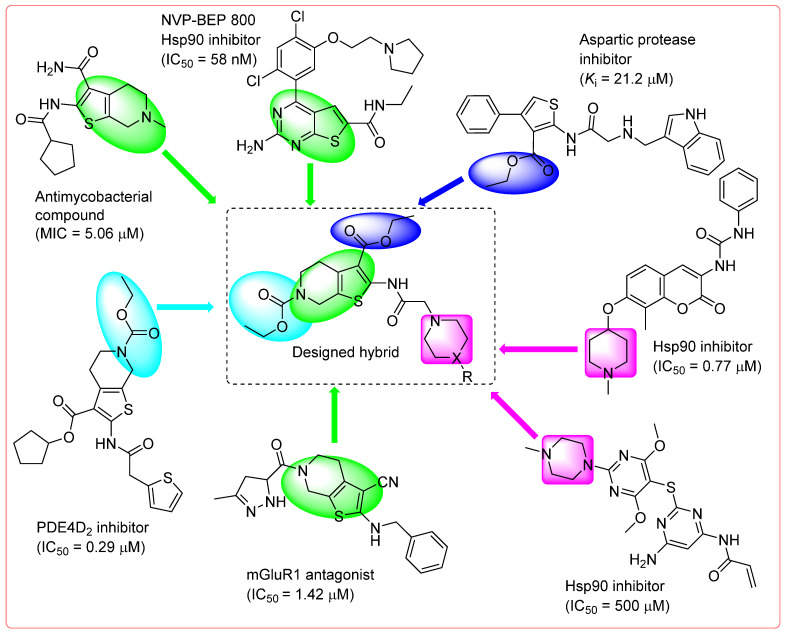
Literature reported pyridines and pyrimidines to design hybrid.

Nitrogen-containing heterocyclic compounds are effective chemotherapeutic agents in treating various cancers [36]. The ring annulation of thiophene on the pyridine scaffold resulted in a thienopyridine moiety which remains one of the versatile scaffolds in several medicinal compounds ascribed to their significant pharmacological activities including anticancer activity [33,37,38], acetylcholine esterase inhibition [39], hepatic gluconeogenesis inhibition [40], antiplatelet activity [41,42], ADP-receptor antagonism [43], antibacterial activity [44], anti-inflammatory activity [45], anthelmintic and insecticidal activity [46], and effectiveness in treating neuropathic pain [47]. Thus, thienopyridine acts as the classical bio-isostere of purines and pyrimidines [48] which is perceived as a significant pharmacophore in medicinal compounds. Specifically, NVP-BEP800 is a thienopyrimidine-based synthetic compound and orally bioavailable inhibitor that binds to the N-terminal ATP-binding pocket. NVP-BEP800 exhibited Hsp90 inhibition at an IC_50_ of 58 nM against breast cancer [49]. This molecule has inspired us to design thienopyridine-based molecules as novel inhibitors of Hsp90, for the first time. The presence of ethyl ester groups at a strategic position on the heterocyclic compounds showed better pharmacological properties due to hydrogen bonding interactions involving the carbonyl groups and the aliphatic hydrophobic interactions involving ethyl groups. Moreover, ester groups can undergo metabolic transformation by the enzyme ‘esterase’ leading to lower toxic effects, besides offering optimal pharmacokinetic properties to medicinal agents [50,51,52]. On the other hand, secondary heterocyclic amines such as piperazines, piperidines, morpholine, and its bio-isosteric thiomorpholine, in the medicinal compounds act on different molecular targets, treating various diseases including cancer, and were crucial fragments in medicinal compounds [53,54,55,56,57,58]. Moreover, the amide bond is an established linker group for synthesizing hybrid molecules [59,60,61]; thus, the heterocyclic moieties designed were tethered through an amide linkage. In the novel design of medicinal compounds, the concept of hybridization is still widely employed in research [62]. Figure 1 illustrates biologically active heterocyclic compounds [52,57,63,64,65,66] towards the design of the target hybrid.

With these exciting reports, and as a continuation of our efforts to find novel heterocyclic compounds, we are interested in synthesizing and evaluating thieno[2,3-*c*]pyridine derivatives as anticancer agents. To better understand the binding of the synthesized compounds toward Hsp90, and to derive a plausible binding mode, an in silico molecular docking analysis was executed using the X-ray-solved structure of Hsp90. Further, in silico predictions of pharmacokinetic ADME and drug-like properties were conducted to establish the drug likeliness of the synthesized compounds.

## 2. Results and Discussion

### 2.1. Chemistry

The synthetic route followed for the titled compounds **6(a–k)** is shown in Scheme 1. The starting compound diethyl 2-amino-4,7-dihydrothieno[2,3-*c*]pyridine-3,6(5*H*)-dicarboxylate **3** was obtained via a well-known Gewald reaction [67] involving ethyl 4-oxo-piperidine-1-carboxylate **1**, ethyl cyanoacetate **2**, and powdered sulfur. A chemical reaction of **3** with chloroacetyl chloride in the presence of basic medium triethylamine yielded an intermediate compound **4** [39]. The nucleophilic displacement of chlorine in intermediate **4** using different heterocyclic secondary amines **5(a–k)** in dry tetrahydrofuran yielded the target compounds **6(a–k)**. The spectral images of FT-IR, ^1^H NMR, ^13^C NMR, and HRMS of all the synthesized compounds are provided in the Supplementary Materials (Figures S1–S48).

### 2.2. Anticancer Evaluation

#### 2.2.1. MTT Assay

An MTT assay [68] was used to evaluate the final compounds’ anticancer activity against MCF7 and T47D (breast cancer cells), HSC3 (head and neck cancer cells), and RKO (colorectal cancer cells) cell lines, using cisplatin as a reference standard. Table 1 presents the percentage growth inhibition of all compounds against the various cancer cell lines.

The growth inhibition was determined at increasing concentrations (1 μM, 10 μM, and 100 μM), and we observed concentration-dependent percentage inhibition by the synthesized compounds (Table 1). In the anticancer evaluation against breast cancer MCF7 cell lines, a thiomorpholine-substituted hybrid compound **6i** showed the highest percentage inhibition of 95.33% which is comparable to the standard drug cisplatin (97.41%) at a concentration of 100 μM. However, at 10 μM, **6i** exhibited moderate inhibition (39.11%) compared to the standard drug (57.32%), and at 1 μM, **6i** demonstrated weak inhibition. Similarly, the piperidine-substituted hybrid **6a** exhibited a potential inhibition of 91.56% comparable to the standard drug cisplatin (97.41%) at a concentration of 100 μM. At 10 μM, **6a** exhibited moderate inhibition (39.45%) compared to the standard drug (57.32%), and at 1 μM, **6a** demonstrated weak inhibition. At a concentration of 100 μM, compounds **6(b–e)**, **6h**, **6j**, and **6k** displayed moderate inhibition ranging from 74.21% to 83.16% compared to cisplatin. Compounds **6f** and **6g** displayed weaker inhibition at the 100 μM concentration. However, these compounds displayed weaker or no inhibitions at 1 μM. Against the other breast cancer cells (T47D cell lines), compounds **6a** and **6i** displayed good inhibition of 88.08% and 83.92%, respectively, and are comparable to cisplatin (98.20%) at the 100 μM concentration. In the case of 10 μM, and 1 μM concentrations, these compounds showed weaker inhibition. A similar pattern of inhibition profiles was observed for other compounds like the inhibitory profiles displayed against MCF7 cell lines.

**Figure 2:**
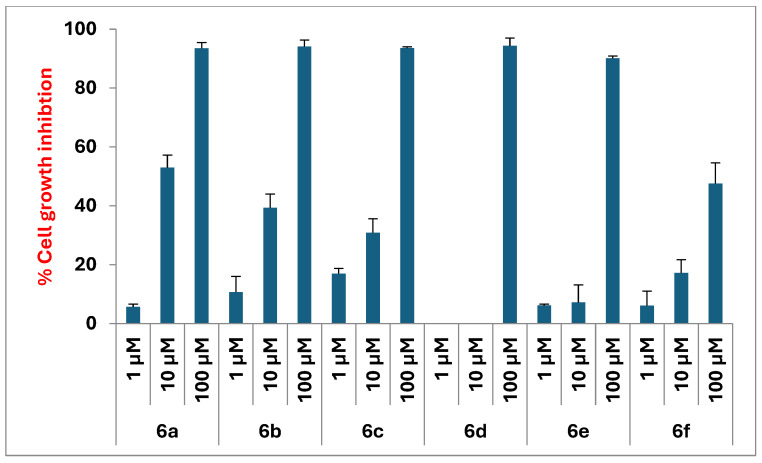
% Cell growth inhibition of the compounds **6(a–f)** against RKO cell line.

**Figure 3:**
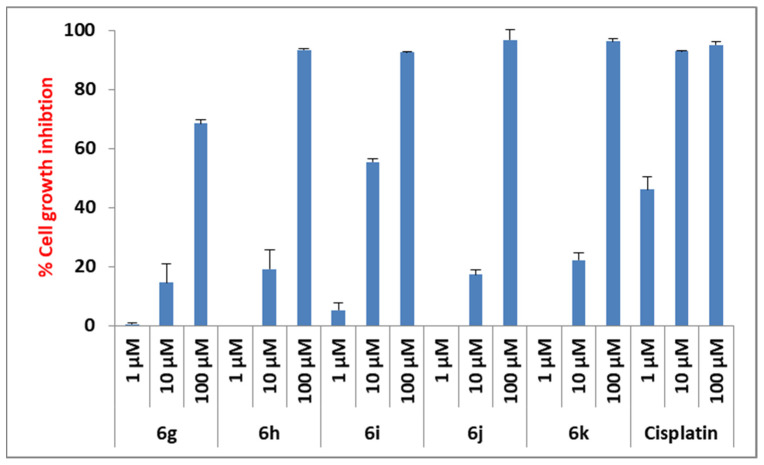
% Cell growth inhibition of the compounds **6(g–k)** and cisplatin against RKO cell line.

In the screening against head and neck cancer cell line HSC3, compounds **6i** and **6a** showed the highest percentage inhibitions of 94.04% and 92.60% in comparison with cisplatin (97.87%) at the 100 μM concentration. However, at the 10 μM concentration level, these compounds demonstrated moderate inhibitions of 62.32% and 59.78%, respectively. At the 1 μM concentration, compound **6i** indicated moderate inhibition (14.84%) compared to cisplatin (29.33%), while most of the other compounds displayed weaker or no inhibition. In the anticancer screening against RKO (colorectal cancer) cell lines, most of the compounds exhibited potent inhibition, with all of them nearly comparable to the standard drug cisplatin at the 100 μM concentration (Figure 2 and Figure 3). Notably, compounds **6a** and **6i** displayed potential inhibition of 93.51% and 92.53%, respectively, at the 100 μM concentration, nearly equal to cisplatin (95.04%). Compounds **6j** and **6k** demonstrated higher inhibition levels of 96.78% and 96.14% compared to cisplatin (95.04%). Nearly equipotent inhibition was shown by other compounds such as **6(b–e)** and **6h** in the range between 90.11% and 94.38%, respectively. Nevertheless, at the 10 μM concentration, compounds **6i** and **6a** showed moderate inhibitions of 55.41% and 53.03% in comparison with cisplatin (92.77%). At the 1 μM concentration level, most of the other compounds showed weaker inhibition. Figure 2 and Figure 3 depict the percentage cell growth inhibition of **6(a–k)** and cisplatin against RKO cell lines, respectively. The percentage growth inhibition against other cell lines is depicted in the Supplementary Materials (Figures S49–S54).

#### 2.2.2. Determination of IC_50_

**Figure 4:**
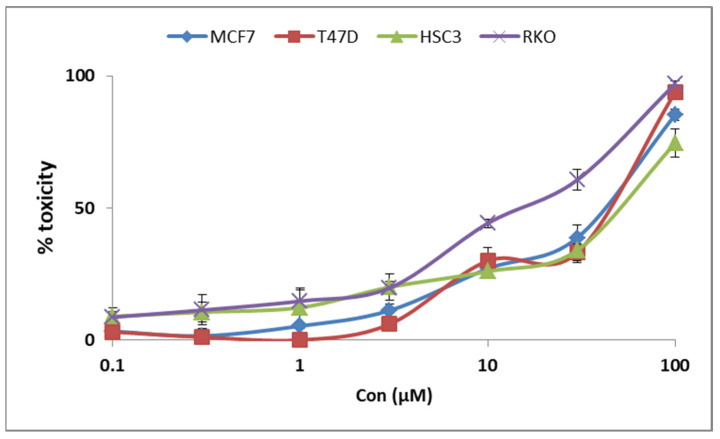
Growth inhibition of **6a** against all the tested cancer cell lines.

**Figure 5:**
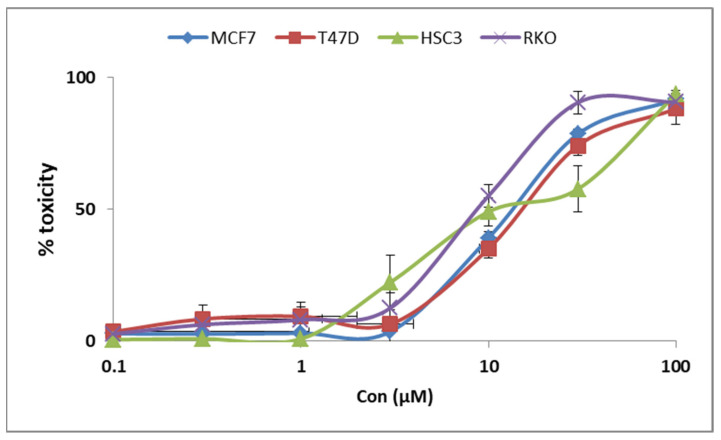
Growth inhibition of **6i** against all the tested cancer cell lines.

**Figure 6:**
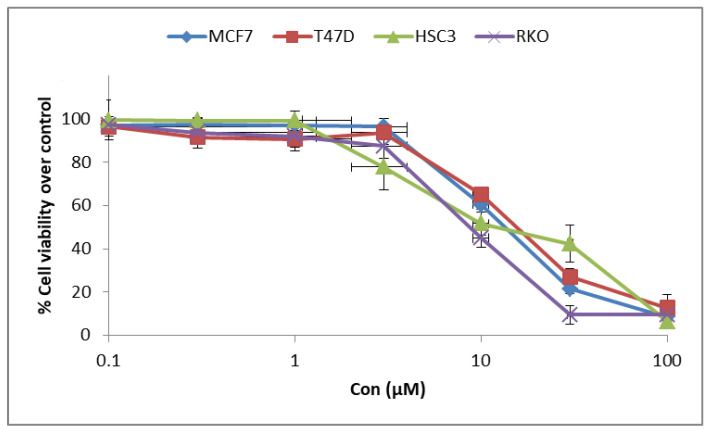
Dose–response curves of **6i** against all the tested cancer cell lines.

A couple of potentially active compounds, **6a** and **6i**, demonstrated good inhibition against all the tested cancer cell lines in the preliminary screening results (Table 1). Hence, these compounds were deputed subsequently to determine IC_50_ values against the studied cancer cell lines. To understand the selectivity of compounds, a normal fibroblast cell line (human periodontal ligament-PDL) is also included in this IC_50_ determination. The results are summarized in Table 2, and the IC_50_ values of the potential compounds **6a** and **6i** are depicted in Figure 4 and Figure 5, respectively. Further, the dose–response curves for compound **6i** are presented in Figure 6.

The results of IC_50_ analysis of compound **6i** displayed potential inhibitions of HSC3 (IC_50_ = 10.8 µM), T47D (IC_50_ = 11.7 µM), RKO (IC_50_ = 12.4 µM), and MCF7 cell lines (IC_50_ = 16.4 µM) indicating a broad spectrum of anticancer activity. On the other hand, compound **6a** showed potent inhibition of head and neck cancer cell line HSC3 at an IC_50_ of 14.5 µM, while it demonstrated moderate inhibition of RKO (colorectal cancer cells) at an IC_50_ of 24.4 µM. Interestingly, both compounds showed no toxicity to normal fibroblast cell lines, PDL, suggesting these compounds can be developed as drug candidates, in the future.

#### 2.2.3. Apoptosis Evaluation

**Figure 7:**
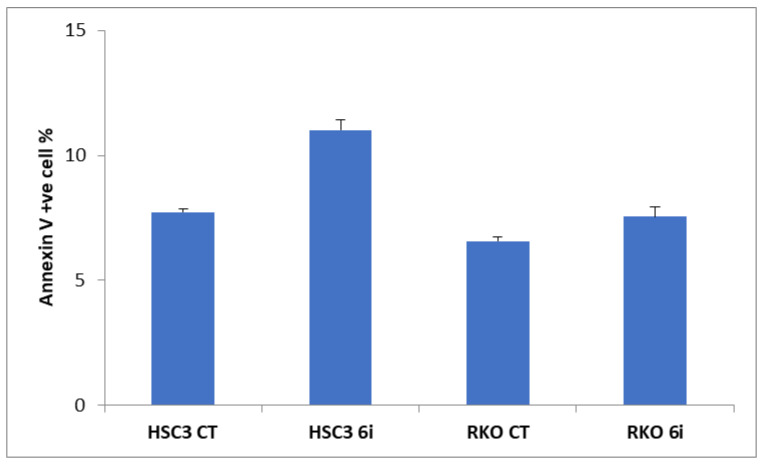
HSC3 and RKO cells exhibit positive Annexin V staining with control and after treatment with compound **6i**.

**Figure 8:**
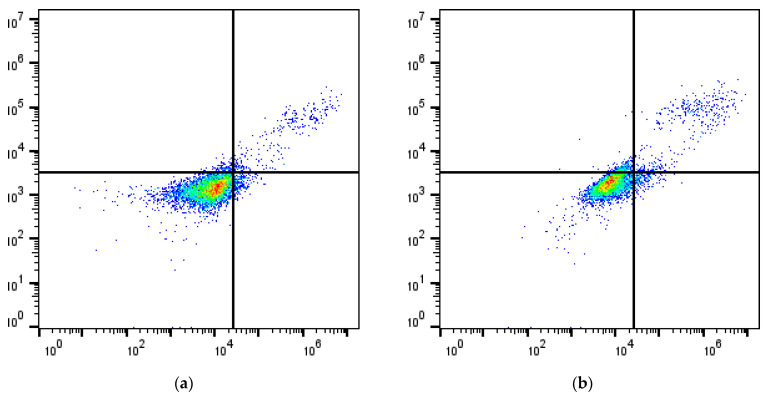

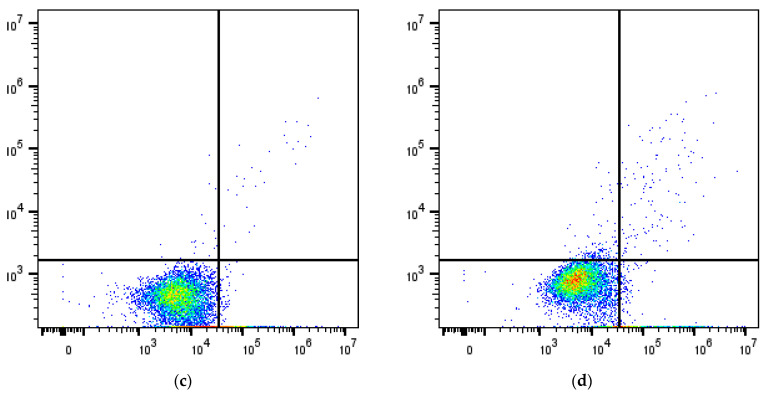
(**a**) HSC3 control, (**b**) HSC3 treated by the compound **6i**, (**c**) RKO control, and (**d**) RKO treated by the compound **6i**. *X*- and *Y*-axis indicate Annexin V and PI fluorescence intensity, respectively. Numbers in the right upper and right lower quadrants denote percentages of late and early apoptotic cells, respectively (Annexin V positive).

To further examine the processes responsible for reduced cell viability, apoptosis was evaluated by Annexin V/Propidium Iodide (PI) double staining, as outlined in the experimental section [69] by considering the best active compound **6i**. The findings illustrated in Figure 7 indicated that the proportions of apoptotic HSC3 and RKO cells exhibiting positive Annexin V staining were significantly lower after treatment with compound **6i**. The representative fluorescence images are provided in Figure 8. Annexin V/PI staining detects very little increase in Annexin-positive cells; **6i**-treated cells were stained with Annexin V and PI for fluorescence-activated cell sorting (FACS) analysis. The results establish that **6i** may promote cell death by processes distinct from apoptosis, deserving further investigation into alternative pathways, such as autophagy or ferroptosis.

#### 2.2.4. Cell Cycle Analysis

**Figure 9:**
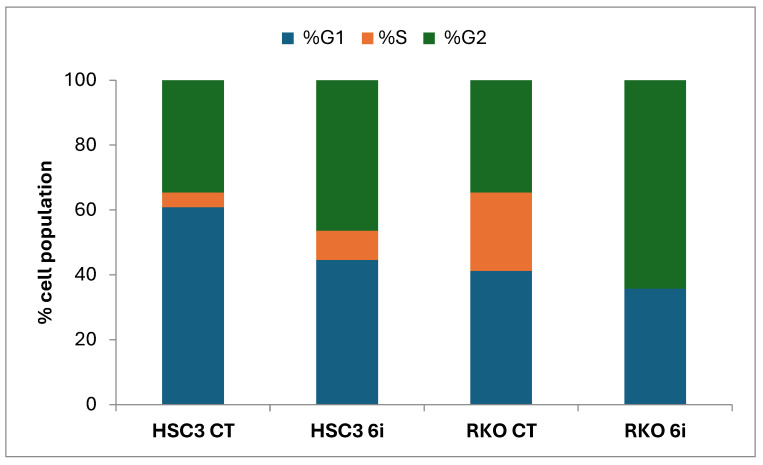
Cell cycle (G_1_, S, and G_2_) analysis of HSC3 and RKO cells under control and compound **6i** treatments, respectively.

In a subsequent set of experiments, we aimed to evaluate the effect of compound **6i** on the cell cycle. Treatment with **6i** led to a significant accumulation of HSC3 and RKO cells in the G2 phase (HSC3: control, 34.6% ± 3.2% vs. **6i**, 46.4% ± 3.6%; RKO: control, 34.5% ± 2.9% vs. **6i**, 64.2% ± 3.5%). Correspondingly, the proportion of cells in the G1 and S phases was markedly reduced, consistent with a G2 phase arrest (Figure 9). The observed accumulation of HSC3 and RKO cells in the G2 phase following treatment suggests that **6i** disrupted the normal progression of the cell cycle. This finding is significant as it indicates that **6i** may exert its anticancer effects by halting cell division at a critical checkpoint, thereby preventing tumor cell proliferation.

### 2.3. Molecular Modeling Studies

#### 2.3.1. Molecular Docking

Molecular docking simulation assists in the design and development of new drugs [70]. To gain more insight into the binding mode of the hybrids, molecular docking (in silico) was conducted against the Hsp90 target. The crystal structure of the Hsp90 (PDB ID: 2WI6) [71] target was selected as a suitable protein in our docking experiments. The literature pointed out that the protein Hsp90 and its chaperone activity are crucial in cancer progression [72] and are involved in drug resistance [73]. Initially, the simulation protocol was verified by redocking the native ligand (ZZ6) into the Hsp90 protein using the Glide XP (extra precision) of Schrodinger suite 2023_2 (accessed on 20 December 2023). In the docked pose of ZZ6, two hydrogen-bonding interactions were noted (i) -NH group of amide in ZZ6 with Gly97, and (ii) amine moiety (-NH_2_) in ZZ6 with Asp93, at a distance of 1.96 Å and 2.04 Å, respectively (Figure S55).

**Figure 10:**
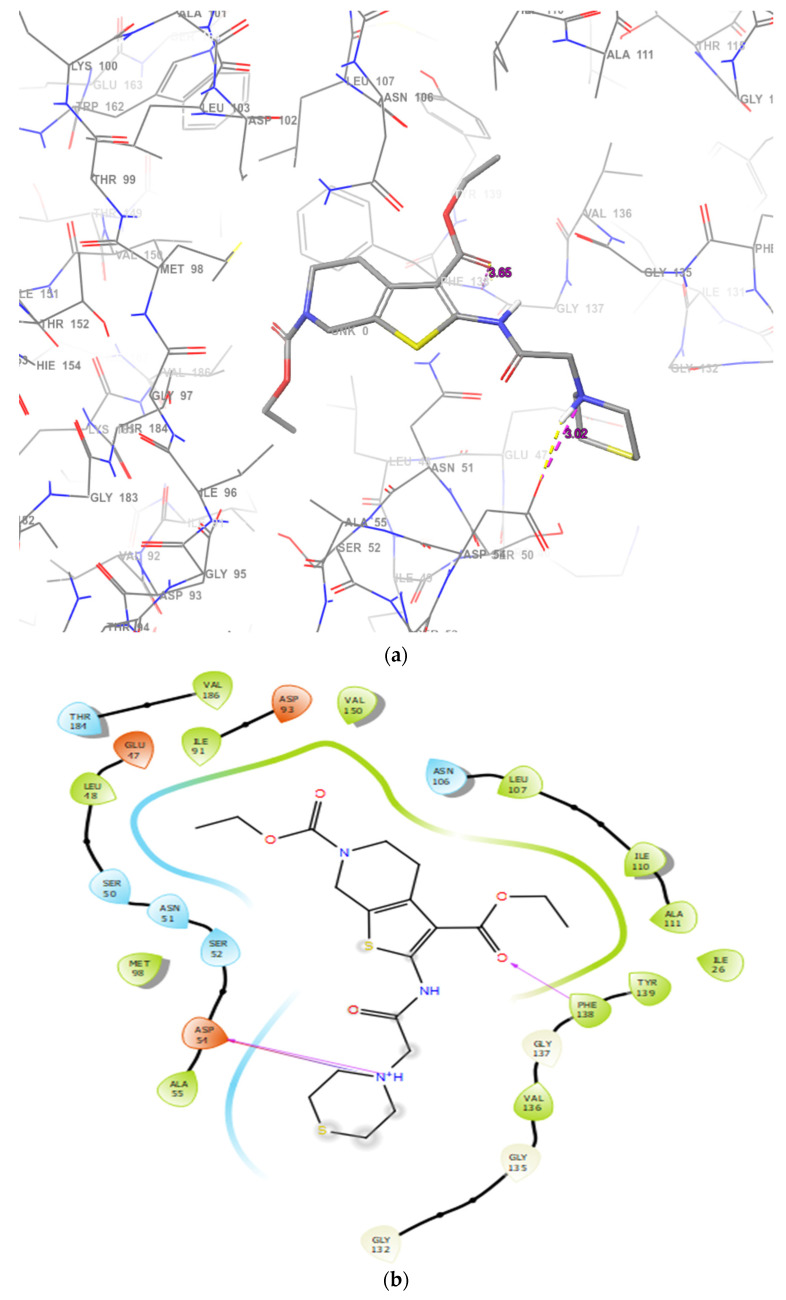
The molecular interactions of **6i** in (**a**) 3D and (**b**) 2D views. Nonpolar hydrogen atoms were hidden for clarity, and pink dashed lines indicate hydrogen bond interactions.

The docking results of the synthesized ligands are organized in Table 3 which demonstrates that most of the compounds interacted well with Hsp90. According to the IC_50_ determination, ligand **6i** was selected to observe the binding mode and molecular interactions (Figure 10a). The protonated nitrogen of the thiomorpholine group in **6i** exhibited a strong hydrogen bond interaction with the carbonyl oxygen of Asp54. Between the thiomorpholine moiety and the amino acid Asp54, a salt bridge type of interaction is also observed. The ethyl ester group is concealed by residues of Ile110, Ala111, and Tyr139. In particular, the carbonyl oxygen of the ethyl ester moiety interacted with the aromatic amino acid residue Phe138 through hydrogen bonding. Similarly, the ester group of the pyridine moiety was enclosed by Asn51, Ser52, Ala55, Ile91, Asp93, and Val150 showing significant hydrophobic interactions. Further, the thiomorpholine moiety was buried with various amino acids such as Asp54, Gly132, Gly135, and Val136. Thus, all the crucial interactions of **6i** with Hsp90 contributed positively to better anticancer activity. Figure 10b shows the 2D interaction plot of ligand **6i**. The docking pose of all other ligands is given in the Supplementary Materials (Figures S56–S65). 

#### 2.3.2. ADME Prediction

Lipinski’s rule of five and ADME pharmacokinetic parameters were determined (in silico) to ensure the drug likeliness of the compounds [74], and the results are collected in Table 4. Most compounds complied with Lipinski’s rule of five except **6f**, **6g**, **6j**, and **6k**. All these four compounds had a calculated molecular weight of 524.631, 530.638, 518.584, and 502.587, correspondingly, which is more than the recommended value of 500 [75]. Similarly, the compounds **6f**, **6j**, and **6k** showed the calculated hydrogen bond acceptors as 10.5, 11.5, and 10.5 against the maximum recommended value of 10. Hence, the three compounds violated two parameters of Lipinski’s rule of five; therefore, these three compounds are not suitable for new drug development. Compound **6g** violated one parameter (molecular weight) according to Lipinski’s rule of five for drug likeliness. Based on the results of drug-likeness predictions, compounds **6a**, **6b**, **6c**, **6d**, **6e**, **6h**, and **6i** possessed drug-like features; therefore, they can be further considered for drug development.

Moreover, the pharmacokinetic parameters of absorption, distribution, metabolism, and excretion (ADME) are crucial in determining drug efficacy and safety [76]. QPlogPo/w (partition coefficient) and QPlogS (water solubility) are generally determined to predict the absorption and distribution of drugs [67]. QPlogPo/w indicates a sophisticated model representing the biomolecular interaction due to solvent partitioning data between *n*-octanol and water [77]. The calculated QPlogPo/w values of all the compounds are observed to be in the range between 1.844 and 4.451, indicating that all the values are within the acceptable range (−2 to 6.5) confirming good absorption potential of the synthesized compounds. Three major parameters for oral bioavailability are QPlogS, QPPCaco, and % human oral absorption [78]. QPlogS is a representative solubility property obtained in the range of −6.392 to −3.08, respectively, for all the compounds indicating that they are within the acceptable range of −6.5 to 0.5. This confirms that all the compounds have good aqueous solubility and can contribute to drug absorption [79]. Caco-2 cell lines are generally used as an intestinal permeability model, wherein the QPPCaco cell permeability prediction model is used to mimic absorption across the human intestinal mucosal membrane. Moreover, the QPPCaco permeability parameter relates to the metabolism and the transportation of drugs via suitable bio-membranes [80]. From the results, it was noteworthy to mention that the QPPCaco values are between 18.448 and 145.669. For good QPPCaco permeability, the prescribed values must be more than 500 nm/s, whereas less than 25 indicates poor permeability. From the results, compound **6c** is predicted to have poor permeability according to QPPCaco, whereas the remaining compounds confer acceptable permeability. The % human oral absorption computed for the compounds ranged between 48.7% and 86.3%. The acceptable value of more than 80% is considered to be suitable for oral administration [81]. The compounds **6a** (84.5%), **6b** (86.3%), and **6i** (84.1%) showed good % human oral absorption and can be taken further for the development of these molecules as drug candidates.

QPlogHERG is a representative cardiotoxicity parameter that principally indicates the predicted IC_50_ value for blockage of HERG K+ channels [82]. The computed values of QPlogHERG for all the compounds ranged between −7.308 and −5.959, which is within the concerned standard value of below −5. Thus, all the compounds were non-cardiotoxic, indicating good safety. The calculated apparent MDCK cell permeability (QPPMDCK) is an indicator of the blood–brain barrier (BBB) penetration and mimics the passive transport of the drug candidates [83]. Most of the compounds demonstrated MDCK cell permeability falling within the prescribed range of 25–500 nm/s and can undergo passive absorption. However, three compounds, namely **6c** (11.595), **6d** (23.224), and **6e** (23.758), showed MDCK cell permeability values below 25 indicating poor BBB penetration. QPlogKhsa is a representative indicator for predicting the binding of drug candidates to human serum albumin. Human serum albumin is the major protein found in the plasma that determines the active concentration of drugs available for pharmacological actions [84]. The recommended value for the QPlogKhsa parameter is between −1.5 and 1.5, and the computed values of all the compounds are obtained within the acceptable range. Thus, the synthesized ligands have a high drug availability within the systemic blood circulation and exhibit high accessibility to the target protein. From all the above observations, the most suitable compound is **6i** which can be developed as an appropriate drug candidate for pre-clinical and clinical studies. This fact is also supported by the encouraging results of anticancer screening assays.

### 2.4. Structure–Activity Relationship (SAR) Study

**Figure 11:**
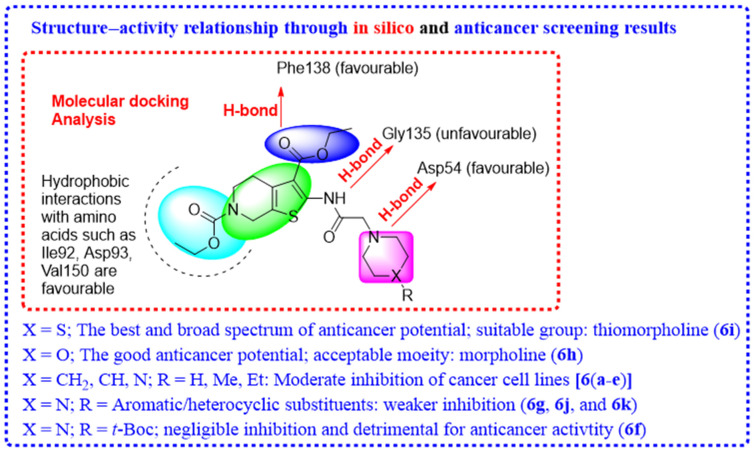
Structure–activity relationship of compounds **6(a–k)** extracted using in silico and in vitro studies.

A structure–activity relationship (SAR) study was conducted using the results extracted from the anticancer screening assays in association with molecular docking interactions. Figure 11 presents the SAR analysis of the target structure and the effects of substituents’ contributions to anticancer activity.

Among the anticancer-screened derivatives, heterocyclic secondary amine (thiomopholine)-substituted compound **6i** provided a significantly higher percentage inhibition against all the tested cancer cell lines. This compound exhibited two hydrogen bonding interactions with active-site amino acids of Hsp90, besides exhibiting drug likeliness and favorable ADME pharmacokinetic properties, and subsequently contributed to its potency. The presence of bioisosteric substituent morpholine to the thienopyridine moiety (**6h**) resulted in a slight decrease in the inhibitory profiles compared to **6i**. Simple piperidine- and *N*-methyl piperidine-substituted compounds (**6a** and **6b**) exhibited relatively good inhibition against all the tested cancer cell lines and exhibited favorable molecular interactions with Hsp90, besides having acceptable drug likeliness. Notably, the carbonyl oxygen of ethyl ester in **6b** showed hydrogen bonding with Phe138, favorably contributing to the anticancer activity. Compound **6(c–e)** bearing piperazine, *N*-methyl piperazine, and *N*-ethyl piperazine displayed moderate inhibition and showed at least one unfavorable molecular interaction with Hsp90. The aromatic (methoxy phenyl) and heterocyclic pyrimidinyl-substituted compounds (**6g** and **6k**) displayed weaker inhibition which is also consistent with molecular docking, wherein these compounds displayed no specific molecular interactions with Hsp90. On the other hand, furoyl-substituted compound **6j** displayed one unfavorable interaction with Gly135 which is detrimental to the anticancer activity. ADME prediction of these three compounds (**6g**, **6j**, and **6k**) indicated that they violated Lipinski’s rule; hence, they will not be considered further as drug candidates. *N*-Boc-substituted piperazine compound **6f** indicated a very weak inhibitory profile which may be attributed to its unfavorable molecular interaction with Hsp90. This substituent is detrimental to the anticancer activity since it is unable to display drug likeliness as predicted by Lipinski’s rule of five.

## 3. Materials and Methods

### 3.1. General Information

All the solvents and chemicals were obtained from Merck (MERCK, Rahway, New Jersey, USA) and were of analytical grade and used without purification. The monitoring of the reaction progress was performed by thin-layer chromatography (TLC) plates, and spots were observed under a UV lamp. Infrared spectra were recorded using the Bruker Invenio S FT-IR spectrometer (Billerica, MA, USA) using the ATR technique. The ^1^H-NMR and ^13^C-NMR spectra were recorded on a Bruker AVANCE 400 and 100 MHz (Bruker, Rheinstetten/Karlsruhe, Germany) spectrometer, respectively. The chemical shifts (*δ*) are specified in parts per million (ppm), and the coupling constants (*J*) are designated in Hertz (Hz) values. TMS was used as an internal standard, and deuterated CDCl_3_ was used as a solvent for NMR experiments. High-resolution mass spectra (HRMS) are recorded using a Bruker Daltonics Apex IV, 7.0 T Ultra Shield Plus (BRUKER, Karlsruhe, Germany) under positive ion mode through electrospray ionization.

### 3.2. Synthesis of Compounds ***3*** and ***4***

Compounds **3** and **4** were synthesized according to the reported protocols [37,39].

### 3.3. General Synthetic Protocol of Diethyl 2-(substituted)acetamido)-4,7-dihydrothieno[2,3-c]pyridine-3,6(5H)-dicarboxylate ***6(a–k)***

To a suspension of the intermediate **4** (0.001 mol; 0.375 g) in 3 mL of dry tetrahydrofuran (THF,) appropriately substituted secondary amines **5(a–k)** (0.0015 mol) were added and stirred for two hours at a temperature range of 55 °C–60 °C. After completion (as monitored by TLC), an excess of THF was evaporated, and the mixture was triturated well with water to obtain a slurry. After filtration, the residue was recrystallized using absolute alcohol to attain pure final compounds **6(a–k)**.

#### 3.3.1. Diethyl 2-(2-piperidin-1-yl)acetamido)-4,7-dihydrothieno[2,3-*c*] pyridine-3,6(5*H*)-dicarboxylate (**6a**)

Golden yellow solid; yield 82%; mp 113–115 °C; FTIR (ATR, *ν_max_*, cm^−1^): 3229 (NH), 1672 (C=O of ester), 1561 (C=O conjugated to piperidine), 1531 (C=O of amide); ^1^H NMR: δ 1.27 (t, *J* = 6.80 Hz, 3H, ethyl), 1.37 (t, *J* = 7.20 Hz, 3H, ethyl), 1.49 (br s, 2H, CH_2_), 1.72 (br s, 4H, CH_2_), 2.57 (br s, 4H, CH_2_), 2.89 (br s, 2H, CH_2_), 3.22 (br s, 2H, CH_2_ of acetamido group), 3.69 (t, 2H, CH_2_), 4.17 (q, *J* = 7.20 Hz, 2H, ethyl), 4.35 (q, *J* = 7.20 Hz, 2H, ethyl), 4.55 (br s, 2H, CH_2_), 12.17 (br s, 1H, NH) ppm; ^13^C NMR: δ 14.57 (CH_3_ of ester), 14.87 (CH_3_ of ester), 23.92 (C-4 of fused ring), 26.04 (4′C of piperidine), 26.56 (2CH_2_; 3′,5′-piperidine), 41.41 (C-5 of fused ring), 42.84 (C-7 of fused ring), 55.23 (2CH_2_; 2′,6′-piperidine), 60.62 (CH_2_ of ester), 61.75 (CH_2_ of ester), 62.13 (CH_2_ of amide), 111.94 (C-3 of thiophene), 122.79 (C-8 of fused ring), 130.25 (C-9 of fused ring), 147.52 (C=O of ester), 155.68 (C=O of ester), 165.24 (C=O of amide), 169.39 (C-2 of thiophene) ppm; HRMS (ESI-TOF) calcd. for C_20_H_30_N_3_O_5_S [M + H]^+^ 424.1828, found 424.1900.

#### 3.3.2. Diethyl 2-(2-(4-methylpiperidin-1-yl)acetamido)-4,7-dihydrothieno[2,3-*c*]pyridine-3,6-(5*H*)-dicarboxylate (**6b**)

Pale yellow solid; yield 83%; mp 105–107 °C; FTIR (ATR, *ν_max_*, cm^−1^): 3253 (NH), 1694 (C=O of ester), 1671 (C=O conjugated to piperidine), 1524 (C=O of amide); ^1^H NMR: δ 1.27 (t, *J* = 7.20 Hz, 3H, ethyl), 1.37 (t, *J* = 6.80 Hz, 3H, ethyl), 1.43 (br s, 3H, -CH_3_), 1.63 (br s, 2H, CH_2_, & 1H, CH), 1.65 (br s, 2H, CH_2_), 2.25 (br s, 4H, CH_2_), 2.89 (br s, 2H, CH_2_), 3.21 (br s, 2H, CH_2_ of acetamido group), 3.69 (t, 2H, CH_2_), 4.17 (q, *J* = 6.80 Hz, 2H, ethyl), 4.35 (q, *J* = 6.80 Hz, 2H, ethyl), 4.54 (br s, 2H, CH_2_), 12.18 (br s, 1H, NH) ppm; ^13^C NMR: δ 14.58 (CH_3_ of ester), 14.88 (CH_3_ of ester), 21.98 (C-4 of fused ring), 26.57 (CH_3_-piperidine), 30.31 (4′C of piperidine), 34.28 (2CH_2_; 3′,5′-piperidine), 41.35 (C-5 of fused ring), 42.84 (C-7 of fused ring), 54.58 (2CH_2_; 2′,6′-piperidine), 60.61 (CH_2_ of ester), 61.75 (CH_2_ of ester), 62.13 (CH_2_ of amide), 111.96 (C-3 of thiophene), 122.87 (C-8 of fused ring), 130.24 (C-9 of fused ring), 147.47 (C=O of ester), 155.69 (C=O of ester), 165.19 (C=O of amide), 169.33 (C-2 of thiophene) ppm; HRMS (ESI-TOF) calcd. for C_21_H_32_N_3_O_5_S [M + H]^+^ 438.1984, found 438.2057.

#### 3.3.3. Diethyl 2-(2-(piperazin-1-yl)acetamido)-4,7-dihydrothieno[2,3-*c*]pyridine-3,6(5*H*)-dicarboxylate (**6c**)

Cream colored solid; yield 85%; mp 92–94 °C; FTIR (ATR, *ν_max_*, cm^−1^): 3240 (NH), 1694 (C=O of ester), 1667 (C=O conjugated to piperidine), 1520 (C=O of amide); ^1^H NMR: δ 1.27 (t, *J* = 7.20 Hz, 3H, ethyl), 1.37 (t, *J* = 7.20 Hz, 3H, ethyl), 2.76 (br s, 2H, CH_2_), 2.82 (br s, 2H, CH_2_), 2.89 (br s, 4H, CH_2_), 3.26-3.31 (m, 4H, CH_2_), 3.69 (br s, 2H, CH_2_ of acetamido group), 4.ν17 (q, *J* = 6.80 Hz, 2H, ethyl), 4.36 (q, *J* = 6.80 Hz, 2H, ethyl), 4.54 (br s, 2H, CH_2_), 12.28 (br s, 1H, NH) ppm; ^13^C NMR: δ 14.53 (CH_3_ of ester), 14.86 (CH_3_ of ester), 26.61 (C-4 of fused ring), 41.33 (C-5 of fused ring), 42.83 (2CH_2_; 3′,5′-piperazine), 45.07 (C-7 of fused ring), 53.50 (2CH_2_; 2′,6′-piperazine), 60.74 (CH_2_ of ester), 61.15 (CH_2_ of ester), 61.79 (CH_2_ of amide), 112.09 (C-3 of thiophene), 123.02 (C-9 of fused ring), 129.18 (C-8 of fused ring), 147.48 (C=O of ester), 155.68 (C=O of ester), 165.47 (C=O of amide), 168.59 (C-2 of thiophene) ppm; HRMS (ESI-TOF) calcd. for C_19_H_29_N_4_O_5_S [M + H]^+^ 425.1780, found 425.1853.

#### 3.3.4. Diethyl 2-(2-(4-methylpiperazin-1-yl)acetamido)-4,7-dihydrothieno[2,3-*c*]pyridine-3,6-(5*H*)-dicarboxylate (**6d**)

Golden yellow solid; yield 80%; mp 95–97 °C; FTIR (ATR, *ν_max_*, cm^−1^): 3247 (NH), 1695 (C=O of ester), 1667 (C=O conjugated to piperidine), 1520 (C=O of amide); ^1^H NMR: δ 1.27 (t, *J* = 7.20 Hz, 3H, ethyl), 1.38 (t, *J* = 7.20 Hz, 3H, ethyl), 2.45 (s, 3H, N-CH_3_), 2.75 (br s, 8H, 4CH_2_), 2.89 (br s, 2H, CH_2_), 3.28 (br s, 2H, CH_2_ of acetamido group), 3.68 (br s, 2H, CH_2_), 4.17 (q, *J* = 7.20 Hz, 2H, ethyl), 4.35 (q, *J* = 7.20 Hz, 2H, ethyl), 4.55 (br s, 2H, CH_2_), 12.24 (br s, 1H, NH) ppm; ^13^C NMR: δ 14.56 (CH_3_ of ester), 14.86 (CH_3_ of ester), 26.57 (C-4 of fused ring), 41.33 (C-5 of fused ring), 42.82 (C-7 of fused ring), 46.10 (CH_3_ of piperazine), 53.57 (2CH_2_; 2′,6′-piperazine), 55.01 (2CH_2_; 3′,5′-piperazine), 60.63 (CH_2_ of ester), 61.18 (CH_2_ of ester), 61.76 (CH_2_ of amide), 111.98 (C-3 of thiophene), 122.99 (C-9 of fused ring), 130.14 (C-8 of fused ring), 147.50 (C=O of ester), 155.66 (C=O of ester), 165.33 (C=O of amide), 168.61 (C-2 of thiophene) ppm; HRMS (ESI-TOF) calcd. for C_20_H_31_N_4_O_5_S [M + H]^+^ 439.1937, found 439.2009.

#### 3.3.5. Diethyl 2-(2-(4-ethylpiperazin-1-yl)acetamido)-4,7-dihydrothieno[2,3-*c*]pyridine-3,6(5*H*)-dicarboxylate (**6e**)

Pale yellow solid; yield 79%; mp 106–108 °C; FTIR (ATR, *ν_max_*, cm^−1^): 3205 (NH), 1672 (C=O of ester), 1523 (C=O of amide); ^1^H NMR: δ 1.16 (t, *J* = 7.20 Hz, 3H, ethyl), 1.27 (t, *J* = 7.20 Hz, 3H, ethyl), 1.37 (t, *J* = 6.80 Hz, 3H, ethyl), 2.58 (q, *J* = 7.20 Hz, 2H, ethyl), 2.72 (br s, 8H, 4CH_2_), 2.89 (br s, 2H, CH_2_), 3.26 (br s, 2H, CH_2_ of acetamido group), 3.69 (t, 2H, CH_2_), 4.17 (q, *J* = 7.20 Hz, 2H, ethyl), 4.35 (q, *J* = 7.20 Hz, 2H, ethyl), 4.54 (br s, 2H, CH_2_), 12.23 (br s, 1H, NH) ppm; ^13^C NMR: δ 11.47 (CH_3_ of ester), 14.53 (CH_3_ of ester), 14.86 (CH_3_ of ethyl), 26.58 (C-4 of fused ring), 41.34 (C-5 of fused ring), 42.82 (C-7 of fused ring), 52.35 (CH_2_ of ethyl), 52.41 (2CH_2_; 2′,6′-piperazine), 52.83 (2CH_2_; 3′,5′-piperazine), 60.66 (CH_2_ of ester), 60.94 (CH_2_ of ester), 61.78 (CH_2_ of amide), 111.96 (C-3 of thiophene), 123.15 (C-9 of fused ring), 130.18 (C-8 of fused ring), 147.53 (C=O of ester), 155.67 (C=O of ester), 165.49 (C=O of amide), 168.36 (C-2 of thiophene) ppm; HRMS (ESI-TOF) calcd. for C_21_H_33_N_4_O_5_S [M + H]^+^ 453.2093, found 453.2166.

#### 3.3.6. Diethyl 2-(2-(4-(tert-butoxycarbonyl)piperazin-1-yl)acetamido)-4,7-dihydrothieno[2,3-*c*]-pyridine-3,6(5*H*)-dicarboxylate (**6f**)

Cream colored solid; yield 68%; mp 90–92 °C; FTIR (ATR, *ν_max_*, cm^−1^): 3185 (NH), 1691 (C=O of ester), 1674 (C=O conjugated to piperidine), 1555 (butoxy-C=O), 1519 (C=O of amide); ^1^H NMR: δ 1.27 (t, *J* = 7.20 Hz, 3H, ethyl), 1.37 (t, *J* = 7.20 Hz, 3H, ethyl), 1.74 (br s, 3H, C-CH_3_), 2.64 (s, 3H, C-CH_3_), 2.89 (s, 3H, C-CH_3_), 3.23 (br s, 2H, CH_2_ of acetamido group), 3.69 (br s, 4H, CH_2_), 3.70 (s, 2H, CH_2_), 3.85 (br s, 2H, CH_2_), 3.99 (br s, 4H, CH_2_), 4.17 (q, *J* = 7.20 Hz, 2H, ethyl), 4.35 (q, *J* = 7.20 Hz, 2H, ethyl), 4.54 (br s, 2H, CH_2_), 12.31 (br s, 1H, NH) ppm; ^13^C NMR: δ 14.44 (CH_3_ of ester), 14.77 (CH_3_ of ester), 25.67 (C-4 of fused ring), 26.48 (3CH_3_-N-Boc), 41.23 (C-5 of fused ring), 42.72 (2CH_2_; 3′,5′-piperazine), 43.73 (C-7 of fused ring), 53.87 (2CH_2_; 2′,6′-piperazine), 60.61 (CH_2_ of ester), 61.67 (CH_2_ of ester), 63.82 (CH_2_ of amide), 66.90 (C-Boc), 111.94 (C-3 of thiophene), 122.98 (C-9 of fused ring), 130.13 (C-8 of fused ring), 147.38 (C=O of ester), 155.55 (C=O of Boc), 162.10 (C=O of ester), 165.40 (C=O of amide), 168.20 (C-2 of thiophene) ppm; HRMS (ESI-TOF) calcd. for C_24_H_37_N_4_O_7_S [M + H]^+^ 525.2305, found 525.2534.

#### 3.3.7. Diethyl 2-(2-(4-(2-methoxyphenyl)piperazin-1-yl)acetamido)-4,7-dihydrothieno[2,3-*c*]-pyridine-3,6(5*H*)-dicarboxylate (**6g**)

Grey solid; yield 71%; mp 118–120 °C; FTIR (ATR, *ν_max_*, cm^−1^): 3273 (NH), 1699 (C=O of ester), 1675 (C=O conjugated to piperidine), 1521 (C=O of amide); ^1^H NMR: δ 1.28 (t, *J* = 7.20 Hz, 3H, ethyl), 1.36 (t, *J* = 7.20 Hz, 3H, ethyl), 2.90 (br s, 8H, CH_2_), 3.27 (br s, 3H, O-CH_3_), 3.34 (br s, 2H, CH_2_ of acetamido group), 3.69 (br s, 2H, CH_2_), 3.88 (br s, 2H, CH_2_), 4.17 (q, *J* = 7.20 Hz, 2H, ethyl), 4.33 (q, *J* = 7.20 Hz, 2H, ethyl), 4.55 (br s, 2H, CH_2_), 6.88-7.04 (m, 4H, Ar-H), 12.28 (br s, 1H, NH) ppm; ^13^C NMR: δ 14.51 (CH_3_ of ester), 14.82 (CH_3_ of ester), 26.55 (C-4 of fused ring), 41.32 (C-5 of fused ring), 42.78 (C-7 of fused ring), 50.59 (2CH_2_; 3′,5′-piperazine), 53.92 (2CH_2_; 2′,6′-piperazine), 55.53 (CH_3_ of methoxy), 60.62 (CH_2_ of ester), 61.33 (CH_2_ of ester), 61.70 (CH_2_ of amide), 111.45 (C-3 of thiophene), 118.41 (CH of 3′-phenyl), 121.08 (CH of 5′-phenyl), 122.12 (CH of 4′-phenyl), 123.13 (C-9 of fused ring & CH of 6′-phenyl), 130.20 (C-8 of fused ring), 141.25 (C-2-methoxy), 147.46 (C=O of ester), 152.40 (N-C-phenyl), 155.61 (C=O of ester), 165.28 (C=O of amide), 168.60 (C-2 of thiophene) ppm; HRMS (ESI-TOF) calcd. for C_26_H_35_N_4_O_6_S [M + H]^+^ 531.2199, found 531.2271.

#### 3.3.8. Diethyl 2-(2-morpholinoacetamido)-4,7-dihydrothieno[2,3-*c*]pyridine-3,6(5*H*)-di-carboxylate (**6h**)

White solid; yield 82%; mp 100–102 °C; FTIR (ATR, *ν_max_*, cm^−1^): 3240 (NH), 1695 (C=O of ester), 1660 (C=O conjugated to piperidine), 1519 (C=O of amide); ^1^H NMR: δ 1.19 (t, *J* = 7.20 Hz, 3H, ethyl), 1.31 (t, *J* = 6.80 Hz, 3H, ethyl), 2.79 (br s, 2H, CH_2_), 3.30-3.41 (br s, 4H, CH_2_), 3.59-3.62 (br s, 4H, CH_2_), 3.67 (s, 2H, CH_2_), 3.88 (br s, 2H, CH_2_ of acetamido group), 4.07 (q, *J* = 7.20 Hz, 2H, ethyl), 4.29 (q, *J* = 7.20 Hz, 2H, ethyl), 4.49 (br s, 2H, CH_2_), 12.11 (br s, 1H, NH) ppm; ^13^C NMR: δ 14.47 (CH_3_ of ester), 14.82 (CH_3_ of ester), 28.53 (C-4 of fused ring), 41.26 (C-5 of fused ring), 42.77 (C-7 of fused ring), 53.41 (2CH_2_; 2′,6′-morpholine), 60.69 (CH_2_ of ester), 61.12 (CH_2_ of ester), 61.71 (CH_2_ of amide), 79.93 (2CH_2_; 3′,5′-morpholine), 112.03 (C-3 of thiophene), 123.04 (C-9 of fused ring), 130.14 (C-8 of fused ring), 147.42 (C=O of ester), 155.60 (C=O of ester), 165.49 (C=O of amide), 168.14 (C-2 of thiophene) ppm; HRMS (ESI-TOF) calcd. for C_19_H_28_N_3_O_6_S [M + H]^+^ 426.1621, found 426.1793.

#### 3.3.9. Diethyl 2-(2-thiomorpholinoacetamido)-4,7-dihydrothieno[2,3-*c*]pyridine-3,6(5*H*)-dicarboxylate (**6i**)

Yellow solid; yield 81%; mp 114–116 °C; FTIR (ATR, *ν_max_*, cm^−1^): 3237 (NH), 1669 (C=O of ester), 1530 (C=O of amide); ^1^H NMR: δ 1.27 (t, *J* = 6.80 Hz, 3H, ethyl), 1.37 (t, *J* = 6.80 Hz, 3H, ethyl), 1.62 (br s, 2H, CH_2_), 2.84-2.89 (m, 8H, CH_2_), 3.27 (br s, 2H, CH_2_ of acetamido group), 3.69 (br s, 2H, CH_2_), 4.17 (q, *J* = 6.80 Hz, 2H, ethyl), 4.35 (q, *J* = 7.20 Hz, 2H, ethyl), 4.55 (br s, 2H, CH_2_), 12.25 (br s, 1H, NH) ppm; ^13^C NMR: δ 14.55 (CH_3_ of ester), 14.88 (CH_3_ of ester), 28.05 (C-4 of fused ring), 41.38 (2CH_2_; 3′,5′-thiomorpholine), 42.84 (C-5 of fused ring), 55.52 (C-7 of fused ring), 60.82 (2CH_2_; 2′,6′-thiomorpholine), 61.79 (CH_2_ of ester), 61.98 (CH_2_ of ester), 62.02 (CH_2_ of amide), 112.13 (C-3 of thiophene), 123.29 (C-9 of thiophene), 130.23 (C-8 of thiophene), 147.44 (C=O of ester), 155.67 (C=O of ester), 165.54 (C=O of amide), 168.24 (C-2 of thiophene) ppm; HRMS (ESI-TOF) calcd. for C_19_H_28_N_3_O_5_S_2_ [M + H]^+^ 442.1392, found 442.1464.

#### 3.3.10. Diethyl 2-(2-(4-(furan-2-carbonyl)piperazin-1-yl)acetamido)-4,7-dihydrothieno[2,3-*c*]-pyridine-3,6(5*H*)-dicarboxylate (**6j**)

White solid; yield 77%; mp 97–99 °C; FTIR (ATR, *ν_max_*, cm^−1^): 3248 (NH), 2980 (Ar-C-H strech), 1695 (C=O of ester), 1670 (C=O conjugated to piperidine), 1519 (C=O of amide), 1220 (Furyl-C-H); ^1^H NMR: δ 1.26 (t, *J* = 6.80 Hz, 3H, ethyl), 1.35 (t, *J* = 7.20 Hz, 3H, ethyl), 2.68 (br s, 4H, CH_2_), 2.84 (m, 2H, CH_2_), 3.29 (br s, 2H, CH_2_ of acetamido group), 3.64 (br s, 4H, CH_2_), 3.93 (br s, 2H, CH_2_), 4.15 (q, *J* = 6.80 Hz, 2H, ethyl), 4.32 (q, *J* = 6.80 Hz, 2H, ethyl), 4.50 (br s, 2H, CH_2_), 6.43 (t, 1H, Ar-H), 6.97 (d, 1H, Ar-H), 7.43 (d, 1H, Ar-H), 12.33 (br s, 1H, NH) ppm; ^13^C NMR: δ 14.43 (CH_3_ of ester), 14.78 (CH_3_ of ester), 26.52 (C-4 of fused ring), 41.23 (C-5 of fused ring), 42.26 (C-7 of fused ring), 42.74 (2CH_2_; 3′,5′-piperazine), 53.57 (2CH_2_; 2′,6′-piperazine), 60.72 (CH_2_ of ester), 60.82 (CH_2_ of ester), 61.70 (CH_2_ of amide), 111.42 (C-3 of thiophene), 112.04 (CH of 4′-furoyl), 116.68 (CH of 3′-furoyl), 123.03 (C-9 of fused ring), 130.08 (C-8 of fused ring), 143.84 (CH of 5′-furoyl), 147.40 (CH of 2′-furoyl), 147.92 (C=O of ester), 155.56 (C=O of furoyl), 159.24 (C=O of ester), 165.58 (C=O of amide), 167.89 (C-2 of thiophene) ppm; HRMS (ESI-TOF) calcd. for C_24_H_31_N_4_O_7_S [M + H]^+^ 519.1835, found 519.1908.

#### 3.3.11. Diethyl 2-(2-(4-(pyrimidin-2-yl)piperazin-1-yl)acetamido)-4,7-dihydrothieno[2,3-*c*]-pyridine-3,6(5*H*)-dicarboxylate (**6k**)

Pale yellow solid; yield 81%; mp 128–130 °C; FTIR (ATR, *ν_max_*, cm^−1^): 3251 (NH), 2980 (Ar-C-H strech), 1697 (C=O of ester), 1670 (C=O conjugated to piperidine), 1525 (C=O of amide); ^1^H NMR: δ 1.27 (t, *J* = 7.20 Hz, 3H, ethyl), 1.35 (t, *J* = 7.20 Hz, 3H, ethyl), 2.73 (br s, 4H, CH_2_), 2.89 (br s, 2H, CH_2_), 3.34 (br s, 2H, CH_2_ of acetamido group), 3.68 (br s, 2H, CH_2_), 4.03 (br s, 4H, CH_2_), 4.17 (q, *J* = 7.20 Hz, 2H, ethyl), 4.32 (q, *J* = 7.20 Hz, 2H, ethyl), 4.55 (br s, 2H, CH_2_), 6.53-6.54 (m, 1H, Ar-H), 8.32-8.34 (m, 2H, Ar-H), 12.36 (br s, 1H, NH) ppm; ^13^C NMR: δ 14.51 (CH_3_ of ester), 14.85 (CH_3_ of ester), 26.61 (C-4 of fused ring), 41.31 (C-5 of fused ring), 42.82 (C-7 of fused ring), 43.75 (2CH_2_; 3′,5′-piperazine), 53.51 (2CH_2_; 2′,6′-piperazine), 60.71 (CH_2_ of ester), 61.26 (CH_2_ of ester), 61.75 (CH_2_ of amide), 110.11 (C-3 of thiophene), 112.08 (CH of 4′-pyrimidinyl), 123.01 (C-9 of fused ring), 130.20 (C-8 of fused ring), 147.49 (C=O of ester), 155.64 (2CH; 3′,5′-pyrimidinyl), 157.88 (N-C-pyrimidinyl), 161.65 (C=O of ester), 165.48 (C=O of amide), 168.34 (C-2 of thiophene) ppm; HRMS (ESI-TOF) calcd. for C_23_H_31_N_6_O_5_S [M + H]^+^ 503.1998, found 503.2071.

### 3.4. Anticancer Evaluation

#### 3.4.1. Cell Culture

The cancer cell lines MCF7 and T47D (breast cancer cells), HSC3 (head and neck cancer cells), and RKO (colorectal cancer cells) were kindly provided by Prof. Stephan Feller, Institute of Molecular Medicine, Martin Luther University, Halle, Germany. The human fibroblast (PDL) cell line was obtained as a kind gift from Prof. Khaled Al-Qaoud (Yarmouk University, Irbid, Jordan). All cell lines were cultured in Dulbecco’s Modified Eagle’s Medium (DMEM) supplemented with 10% fetal bovine serum, 100 µg/mL streptomycin, 100 U/mL penicillin, and 1% non-essential amino acid at 37 °C with 5% CO_2_ in a humidified CO_2_ incubator. The cell cultures were grown to confluence, and the medium was changed two times weekly.

#### 3.4.2. MTT Assay

The MTT (3-[4,5-dimethylthiazol-2-yl]-2,5-diphenyl tetrazolium bromide) assay [68] was used to investigate cell viability and to calculate the half-maximal inhibitory concentration (IC_50_) values of the compounds **6(a–k)** including standard reference cisplatin. Furthermore, 20 µM stock solutions of the compounds were prepared in DMSO where all compounds were completely soluble. Cells were seeded in 96-well tissue culture plates at a density of 6,000 cells/well for MCF7, T47D, and PDL cells and at a density of 4000 cells/well for RKO and HSC3 cells. After 24 h of seeding, investigated cells were treated with three concentrations (1, 10, and 100 µm) of the compounds for 72 h. At the end of the treatment time, the supernatant medium was aspirated, and 50 µL of fresh DMEM containing MTT (0.5 mg/mL) was added to each well. Cells were then incubated at 37 °C for 2–4 h. After that, the medium was completely removed, and 50 µL of DMSO was added to each well to dissolve the precipitated formazan crystals formed by viable cells. Cell viability, represented by the absorbance of the formazan solution, was measured at 570 nm using the Synergy HTX Multimode Reader (BioTek, Vermont, USA), and cell viability was determined as described in the literature [85]. For each concentration, the relative cell viability was calculated as follows: relative cell viability = (mean treatment absorbance/mean control absorbance) × 100%. Assays were performed in triplicate on three independent experiments. Compounds **6a** and **6i** showed considerable cytotoxic activity in the investigated cell lines and were subject to further analysis for IC_50_ determination. Cancer and normal fibroblast cells were seeded in 96-well plates as mentioned previously and treated with serial concentrations of **6a** and **6i** for 72 h. IC_50_ values were then calculated using the four-parameter logistic function in the SigmaPlot software (version 12.5) and presented as the mean ± SD from three independent experiments.

#### 3.4.3. Apoptosis Evaluation by Annexin V/PI Staining

HSC3 and RKO cells were seeded in 6-well plates for 24 h (1 × 10^5^ cells/well) and treated with the IC_50_ concentrations of compound **6i** or DMSO as a vehicle control for 72 h. Floating dead cells and the attached cells were collected, washed with PBS, and resuspended in 100 μL of Annexin V solution for 30 min according to the manufacturer’s instruction (Annexin V-FITC Kit, Miltenyi Biotec, Gaithersburg, MD, USA, Catalog Number 130-092-052) [69]. After Annexin V staining, cells were stained shortly with PI to exclude the dead cells. The quantitative analysis for apoptosis was performed by FACS Accuri C6 Plus (BD BIOSCIENCES, Franklin Lakes, NJ, USA) flow cytometer. About 10,000 events were analyzed using the FlowJo software (version 10).

#### 3.4.4. Cell Cycle Analysis

HSC3 and RKO cells were seeded in 6-well plates for 24 h (1 × 10^5^ cells/well) and treated with the IC_50_ concentrations of compound **6i** or DMSO as a vehicle control for 72 h. The supernatant with the floating dead cells and the attached cells from each well were collected and fixed in cold ethanol at −20 °C for 30 min [69]. Cells were stained with a Propidium iodide (PI) working solution (1 µg/mL PI and 10 µg/mL RNase A in PBS) for 30 min at 37 °C in the dark and then washed with PBS. Cell fluorescence was measured using the FACS Accuri C6 Plus (BD BIOSCIENCES, Franklin Lakes, NJ, USA) flow cytometer. Histograms of cell counts versus blue fluorescence were generated, and the distribution of cells throughout several cell cycle stages was assessed based on the mean fluorescence intensity values. About 10,000 events were examined utilizing FlowJo software (version 10).

### 3.5. Molecular Modeling Studies

#### 3.5.1. Molecular Docking Protocol

Molecular docking was achieved with the help of the Glide program in Schrodinger Suite (2023_2) (Schrödinger, Inc., New York, NY, USA) running on an Intel Xeon w3 2425-based HP Z4 G5 workstation with Microsoft Windows 11 Pro. During the docking simulations, the structure of the target protein is kept rigid, while providing flexibility to ligands. The docking simulation tasks involve five steps including (i) protein preparation, (ii) grid generation, (iii) ligand preparation, (iv) docking simulation, and (v) binding mode/post-docking analysis.

##### Protein Preparation

The structure of Hsp90 complexed with a ligand ZZ6 (ID: 2WI6) was downloaded from the protein data bank (PDB) [71] and utilized as the target protein for the docking of synthesized compounds. The protein was prepared using the ‘Protein Preparation Wizard’ [86] of Glide using the Optimized Potentials for Liquid Simulations 4 (OPLS4) forcefield [87]. This step involved three major events: (i) preprocessing, (ii) optimization, and (iii) minimization. Initially, the target protein was downloaded to the workspace, missing hydrogen atoms were added to the protein structure, and the heterocyclic state of ligands was verified, accordingly. Then, water molecules in the protein, unwanted co-factors, and metal ions were deleted. If any defects in the amino acids such as missing amino acids or incomplete atoms in amino acids were identified and consequently rectified using the Prime module of Glide. After the preprocessing event, the protein was subjected to refinement through the optimization of the sample–water orientation using the interactive optimizer of the Glide program. Finally, the crucial event of restrained minimization using OPLS4 was carried out to fix the orientation of the amino acid side chains to avoid any steric clashes. The convergence of heavy atoms to an RMSD of 0.3 Å was performed on the entire protein for minimization.

##### Grid Generation

The grid box was created on the prepared protein covering a volume of 20 Å around the ligand (ZZ6) using the receptor-grid generation protocol available in the software in order to define a suitable binding site for the docking. To soften the potential for nonpolar sections of the protein, the van der Waals radii scaling factor was set to 1 with a partial charge cutoff of 0.25. Without applying any constraint, the default setting was used for the generation of the grid, and the resulting grid file was saved for docking simulation.

##### Ligand Preparation

The chemical structures were drawn using a 2D sketcher and subsequently saved into the workspace of the software. The LigPrep [88] protocol was deputed to prepare all the ligands for the docking. LigPrep integrates facilities to generate the possible ionization states at pH 7, tautomers, retaining chirality, creation of possible stereoisomers, adjustment of geometry, and finally conducts the energy minimization for the input ligands. The output file was saved and utilized for docking simulations.

##### Docking Simulation

By specifying the ligands against the receptor grid, the molecular docking simulation was performed using the ligand docking module under Glide extra precision (XP) mode. For docking validation, the native ligand (ZZ6) was removed from the complex and redocked using the Glide (XP) mode. In the output pose viewer file, the receptor option was selected and allowed for the post-docking minimized poses to a maximum of five poses per ligand.

##### Binding Mode Analysis

The generated pose viewer file was imported to the XP visualizer module of the Maestro interface, which is available in the Glide program. Then, each protein–ligand complex was analyzed for the presence of intermolecular interactions such as hydrogen bonding, halogen bonding, hydrophobic, π-π stacking, and salt bridges. Further, the docking pose of the complex was investigated for the binding orientation of the ligands in the binding site of the protein, and the best docking scores were collected for each ligand.

#### 3.5.2. Drug-Likeliness and ADME Prediction

The QikProp (QP) program of Schrodinger was employed for the determination of the drug likeliness and pharmacokinetics of the ligand’s 3D molecular structure, computationally. About 40% of drug candidates were not successfully developed as therapeutic agents due to their poor ADME properties. Hence, an early prediction of such properties can significantly save time and economy. The drug likeliness was majorly decided based on Lipinski’s rule of five which refers to any drug-like compounds and oral availability for which the compounds should not have more than five hydrogen bond donors, not more than 10 hydrogen bond acceptors, and a molecular weight of more than 500 daltons. Moreover, the computed octanol–water partition coefficient (log P) must not exceed 5. On the other hand, the ADME properties such as QPlogKhsa, QPlogHERG, QPPMDCK, QPlogS, % human oral absorption, and QPPCaco [89] are crucial to determine the compound’s pharmacokinetics. QPlogS is an aqueous solubility prediction parameter [90] that determines the drug solubility in blood components which is crucial in drug distribution to the site of action. Cardiac toxicity is one of the serious side effects observed in most of the anti-cancer drugs [91]. Hence, the USFDA (US Food and Drug Administration) recommended that all new compounds be evaluated for their cardiac toxicity which could occur due to blocking of voltage-gated potassium channels called hERG channels [92]. QPlogHERG is a general descriptor indicating cardiac toxicity. Caco-2 cell permeability (QPPCaco), a measure of the gut–blood barrier, is a permeability assay to compute the oral absorption of drugs and represents mechanistic studies of absorption [93]. QPPCaco is an important screening tool in drug discovery to predict intestinal drug permeability [94]. QPPMDCK is an in vitro standard for evaluating drug uptake efficiency through determining permeabilities related to intestinal absorption [95]. The prediction of such properties is crucial for optimal pharmacokinetics for drug candidates, and Madin-Darby canine kidney (MDCK) cells are good candidates for modeling epithelia or determining rapid membrane permeability [96]. QPlogKhsa is a standard parameter for evaluating human serum albumin binding affinity which is crucial for the determination of drug distribution to the target protein [97]. The highly lipophilic structures will generally have a higher affinity to plasma protein, altering the drug distribution properties [98]. Oral bioavailability is a basic descriptor that must be predicted to confer better absorption to new compounds, which depends on their permeability across the cell membranes [99]. The major computed molecular descriptor used to evaluate oral absorption is % human oral absorption which is to be determined before developing the synthesized compounds into new drug candidates [100].

## 4. Conclusions

A simplified route was established for the synthesis of thieno[2,3-*c*]pyridine-based hybrid compounds **6(a–k)** and their characterization, spectroscopically. In vitro anticancer screening was performed against a panel of cancer cell lines comprising MCF7 and T47D (breast cancer cells), HSC3 (head and neck cancer cells), and RKO (colorectal cancer cells) cell lines. The compounds **6a** and **6i** were identified as the most active hybrids showing a percentage of inhibition of 92.6% and 94%, respectively, against HSC3 cell lines. These were further evaluated to determine IC_50_ values against all the studied cancer cell lines. Specifically, compound **6i** demonstrated the highest inhibition (IC_50_ = 10.8 µM) on HSC3 cell lines with no toxicity against normal PDL cell lines (IC_50_ >100 µM). Compound **6i** induced cell death through non-apoptotic mechanisms and halted tumor cell proliferation by arresting the cell cycle at the G2 phase in HSC3 and RKO cell lines. The screening results showed that an increase in activity was noticed for compound **6i** bearing thiomorpholine substitution. The results of molecular docking assisted in understanding the crucial ligand–target molecular interactions. The drug-likeliness and ADME determinations ensured the drug-like properties for most of the compounds. While this study investigated the anticancer properties of compounds against four different cancer cell lines, this number remains limited given the heterogeneity of cancer types. Screening the target compounds across a broader panel of cell lines, including additional subtypes of the cancers, would help to confirm the generalizability of the research findings and account for diversity as well. Such studies will aid in determining the broader spectrum of anticancer activity of compounds **6a** and **6i**. In the present study, a normal human cell line was also included to assess cytotoxicity and selectivity.

This research relies solely on the use of in vitro cell cultures which do not fully replicate the complex tumor microenvironment such as interactions with the immune system, extracellular matrix, or blood supply. Conducting further in vivo studies using appropriate animal models is necessary for the future to assess toxicity, bioavailability, and tumor growth inhibition in models such as xenografts or patient-derived xenografts (PDXs). Moreover, in vivo evaluation, pre-clinical testing, and drug metabolic–pharmacokinetic (DMPK) studies are required before considering these compounds for practical use in cancer therapy. The lead structure optimization, generation of a library of compounds by replacing ester on pyridine ring with methyl and ethyl groups, and evaluation using suitable in vivo models are the future directions of the current study to achieve more potent drug candidates.

## Figures and Tables

**Scheme 1:**
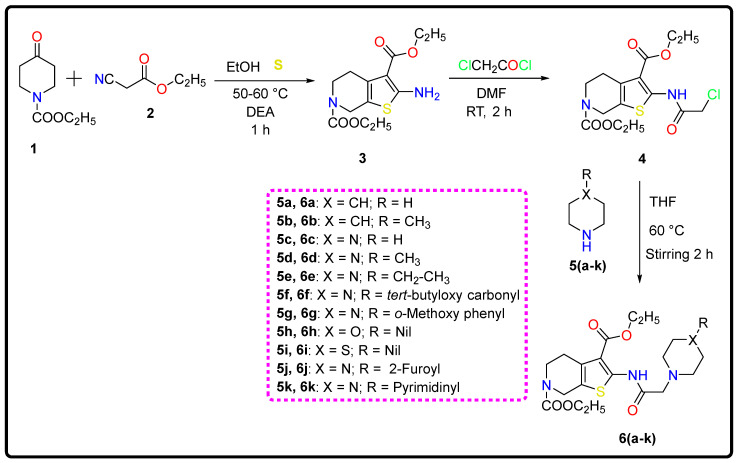
Synthetic route for the target compounds **6(a–k)**.

**Table 1:** Anticancer (% cell growth inhibition) activity of the final compounds **6(a–k)** against a panel of cell lines (MCF7, T47D, HSC3, and RKO).

	MCF7			T47D			HSC3			RKO		
Compd	1 µM	10 µM	100 µM	1 µM	10 µM	100 µM	1 µM	10 µM	100 µM	1 µM	10 µM	100 µM
**6a**	2.96	39.45	91.56	9.31	38.58	88.08	4.26	59.78	92.60	5.71	53.03	93.51
**6b**	2.74	29.71	83.16	7.79	24.36	81.96	4.06	37.96	79.44	10.71	39.34	94.10
**6c**	0	22.09	81.00	3.70	28.11	80.79	6.71	30.03	83.44	17.10	30.87	93.59
**6d**	1.42	3.7	79.32	0	2.54	82.08	0	0	83.89	0	0	94.38
**6e**	4.67	11.71	74.21	5.23	3.12	79.44	3.26	5.51	75.78	6.17	7.24	90.11
**6f**	7.34	10.02	39.02	7.62	16.21	34.09	0	16.78	45.78	6.11	17.21	47.58
**6g**	3.4	6.36	57.33	0	14.24	59.89	0	15.93	56.87	0.53	14.61	68.42
**6h**	0	20.30	77.18	0	17.78	78.17	0	9.88	77.89	8.76	19.07	93.32
**6i**	1.53	39.11	95.33	1.90	35.29	83.92	14.84	62.32	94.04	5.17	55.41	92.53
**6j**	0	16.19	76.48	0	1.19	75.96	0	29.87	86.38	0	17.28	96.78
**6k**	0	12.18	80.04	0	10.09	76.46	0	11.34	86.17	0	22.17	96.14
Cisplatin	11.23	57.32	97.41	29.09	89.65	98.20	29.33	84.74	97.87	46.06	92.77	95.04

Footnote: ‘0’ indicates no growth inhibition.

**Table 2:** IC_50_ values of compounds **6a** and **6i** against cancer cell lines and fibroblasts.

^a^IC_50_ (µM) ± SE					
Compd	MCF7	T47D	HSC3	RKO	PDL
**6a**	73.7 ± 4.3	97.2 ± 4.4	14.5 ± 4.3	24.4 ± 7.2	>100
**6i**	16.4 ± 0.2	11.7 ± 1.1	10.8 ± 3.9	12.4 ± 1.9	>100
**Cisplatin**	5.8 ± 0.9	2.6 ± 0.6	2.7 ± 0.2	1.7 ± 0.4	15.5 ± 2.1

^a^IC_50_: Compound concentration needed to inhibit tumor cell proliferation by 50%.

**Table 3:** Molecular docking results of **6(a–k)**.

Compd	Docking Score	Glide evdW	Glide Ecoul	Glide Energy	Glide Einternal	Glide Emodel
**6a**	−5.133	−40.891	−10.09	−50.981	16.653	−56.89
**6b**	−3.724	−42.288	−5.152	−47.44	6.655	−55.943
**6c**	−5.092	−38.387	−10.613	−49.0	6.527	−54.486
**6d**	−5.398	−42.21	−9.038	−51.248	2.874	−68.58
**6e**	−5.189	−45.566	−9.931	−55.497	9.044	−66.233
**6f**	−4.009	−43.921	−7.194	−51.114	8.275	−59.976
**6g**	−6.292	−36.7	−11.626	−48.325	14.758	−61.394
**6h**	−4.047	−48.142	−2.372	−50.514	10.241	−65.055
**6i**	−4.69	−42.366	−4.124	−46.489	1.121	−64.456
**6j**	−6.273	−46.694	−8.691	−55.385	5.184	−71.452
**6k**	−5.238	−49.733	−8.657	−58.39	6.355	−71.405

**Table 4:** The drug likeliness and in silico ADME properties of **6(a–k)** were calculated using QikProp (QP).

Compd	Drug Likeliness (Lipinski’s Rule of Five)					In Silico ADME Properties					
	Molecular Weight	QPlogP O/W ^a^	H-Bond Donor	H-Bond Acceptor	Violation of Lipinski’s Rule	QPlogS ^b^	QPlogHERG ^c^	QPPCaco ^d^	QPPMDCK ^e^	QPlogKhsa ^f^	% Human Oral Absorption ^g^
**6a**	423.526	3.205	0	8.0	0	−4.96	−6.19	137.743	94.068	0.271	84.1
**6b**	437.553	3.563	0	8.0	0	−5.469	−6.252	142.802	97.685	0.426	83.3
**6c**	424.514	1.844	1	9.5	0	−3.569	−6.905	18.448	11.595	0.16	60.2
**6d**	438.541	1.913	0	10.0	0	−3.08	−6.965	33.921	23.224	0.147	65.8
**6e**	452.568	2.234	0	10.0	0	−3.203	−7.154	34.69	23.758	0.069	67.6
**6f**	524.631	3.693	0	10.5	2	−6.246	−6.69	74.061	48.807	0.41	56.1
**6g**	530.638	4.451	0	9.75	1	−6.392	−7.231	131.915	89.786	0.574	77.2
**6h**	425.499	2.067	0	9.7	0	−3.459	−5.959	145.669	101.112	0.325	77.8
**6i**	441.559	3.184	0	8.5	0	−4.963	−7.294	144.377	183.654	0.1	84.5
**6j**	518.584	2.796	0	11.5	2	−5.082	−7.308	66.205	35.572	0.075	48.7
**6k**	502.587	3/259	0	10.5	2	−5.411	−7.183	94.288	63.348	0.163	55.6

^a^ Predicted log p (octanol/water partition coefficient; acceptable range is −2.0 to 6.5). ^b^ Predicted aqueous solubility (mol/L; acceptable range is −6.5 to 0.5). ^c^ Predicted IC_50_ value for blockage of HERG K+ channels (below −5.0). ^d^ Predicted Caco-2 cell permeability (nm/s; acceptable range: <25 is poor and >500 is good). ^e^ Predicted apparent MDCK cell permeability (nm/s; acceptable range: <25 is poor and >500 is good). ^f^ Predicted binding to human serum albumin (acceptable range is −1.5 to 1.5). ^g^ Percentage of human oral absorption (<25% is poor and >80% is high).

## Data Availability

All of the data generated or analyzed during this study are included within this article and Supplementary Material file; further inquiries can be directed to the corresponding author.

## Supplementary Materials

The following supporting information can be downloaded at https://www.mdpi.com/article/10.3390/ph18020153/s1: FT-IR, ^1^H NMR, ^13^C NMR, and HRMS spectral images (Figures S1–S48), anticancer screening results (Figures S49–S54), docking pose of native ligand (Figure S55), and docking pose of the synthesized ligands (Figures S56–S65).
