# Supplementary material for: Synthesis, Anticancer Screening, and In Silico Evaluations of Thieno[2,3-c]pyridine Derivatives as Hsp90 Inhibitors
Source: Pharmaceuticals (Basel). 2025 Jan 24;18(2):153. doi: 10.3390/ph18020153 (PMC11858597; doi:10.3390/ph18020153)
Supplement: Supplementary file 1 [file pharmaceuticals-18-00153-s001.zip › pharmaceuticals-3441615-supplementary.pdf]

Supplementary Materials

Spectral Images

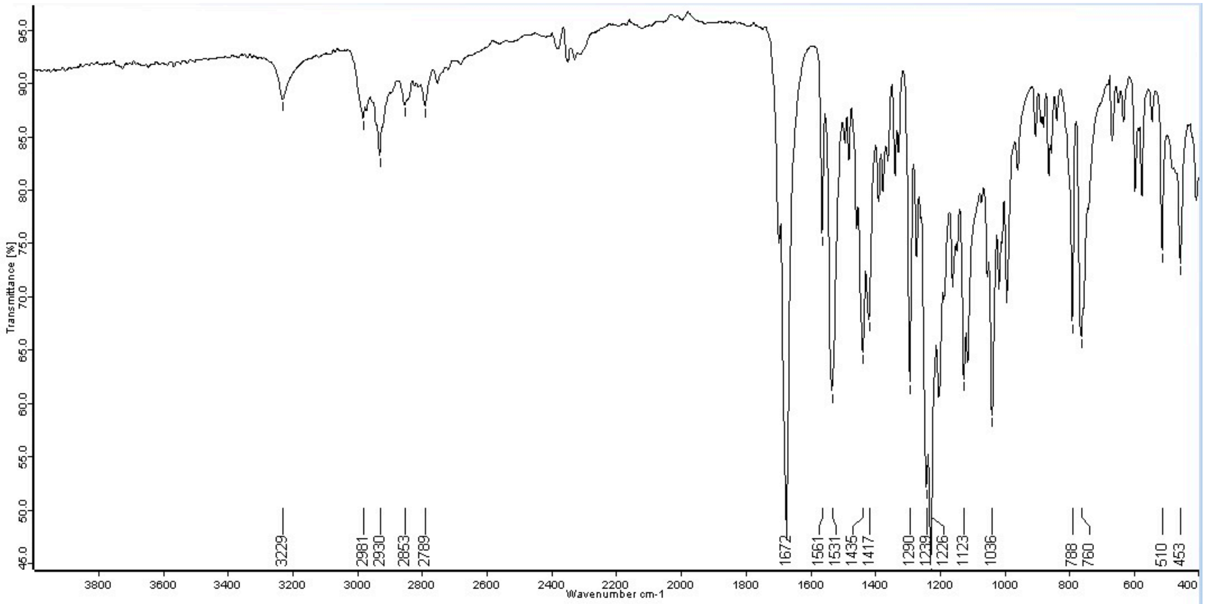

Figure **S1**. FT-IR of **6a**

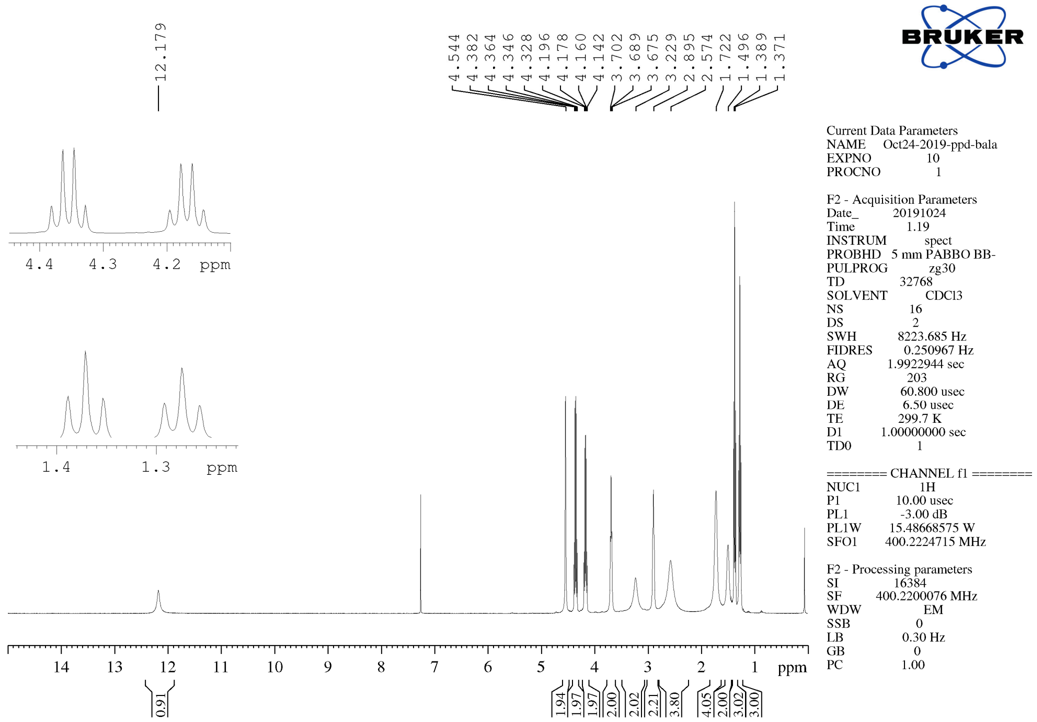

Figure **S2**. ^{1}H NMR of **6a**

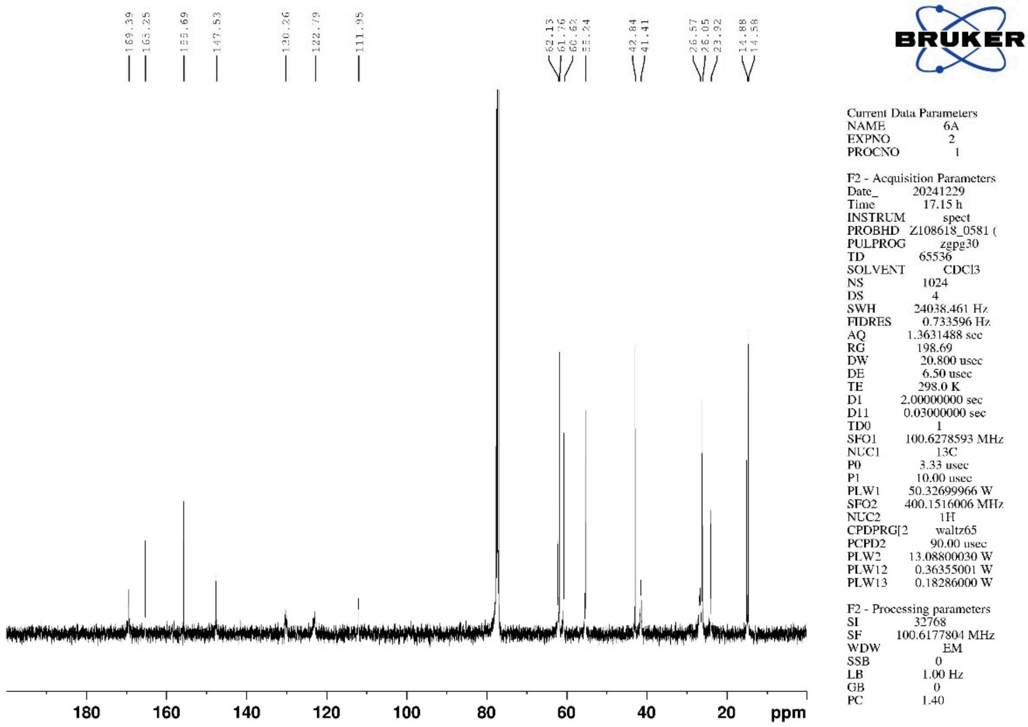

Figure **S3**. ^{13}C NMR of **6a**

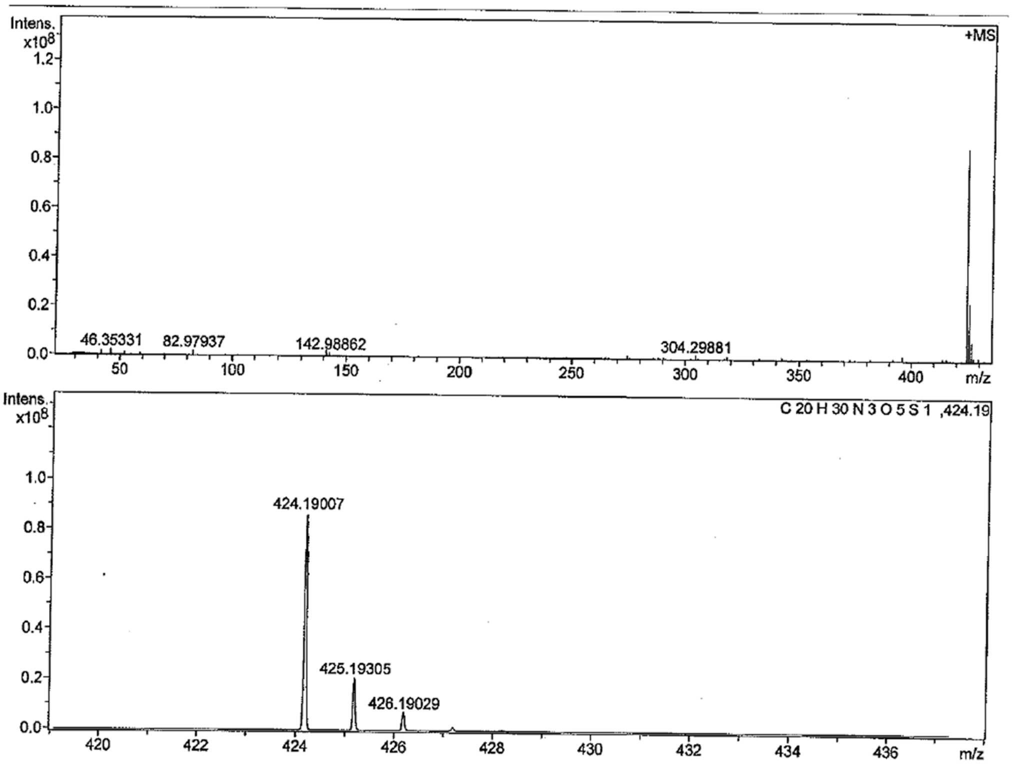

Sum Formula	Sigma	m/z	Err [ppm]	Mean Err [ppm]	Err [mDa]	rdb	N Rule	e⁻
C 20 H 30 N 3 O 5 S 1	0.017	424.19007	4.50	5.05	1.91	7.50	ok	even

Figure **S4**. HRMS of **6a**

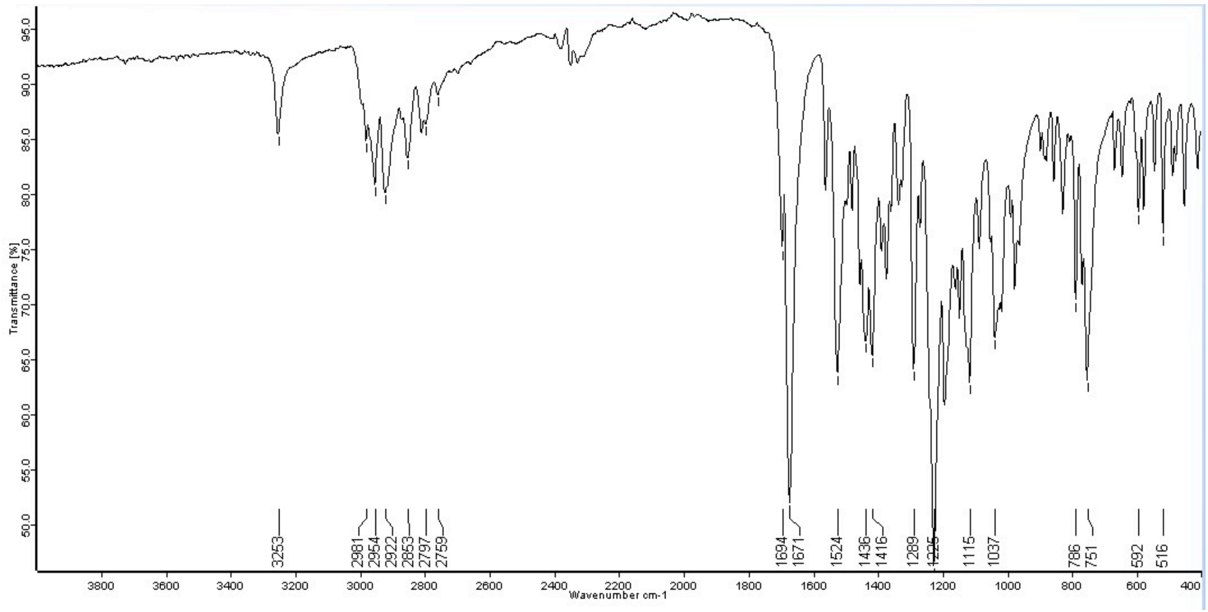

Figure **S5**. FT-IR of **6b**

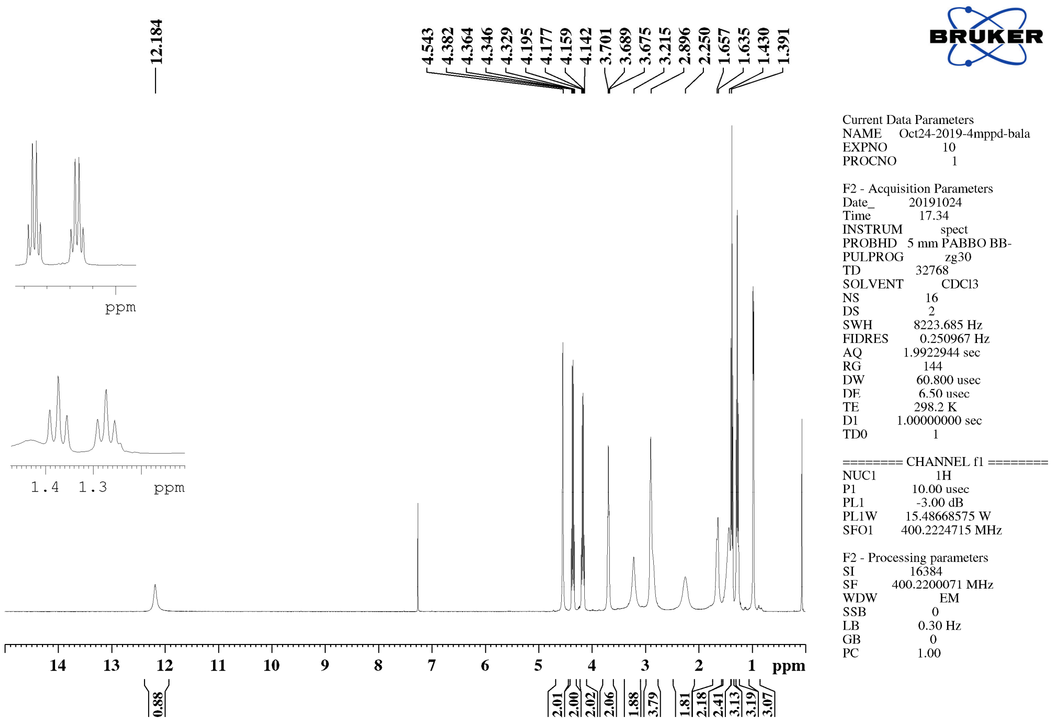

Figure **S6**. ^{1}H NMR of **6b**

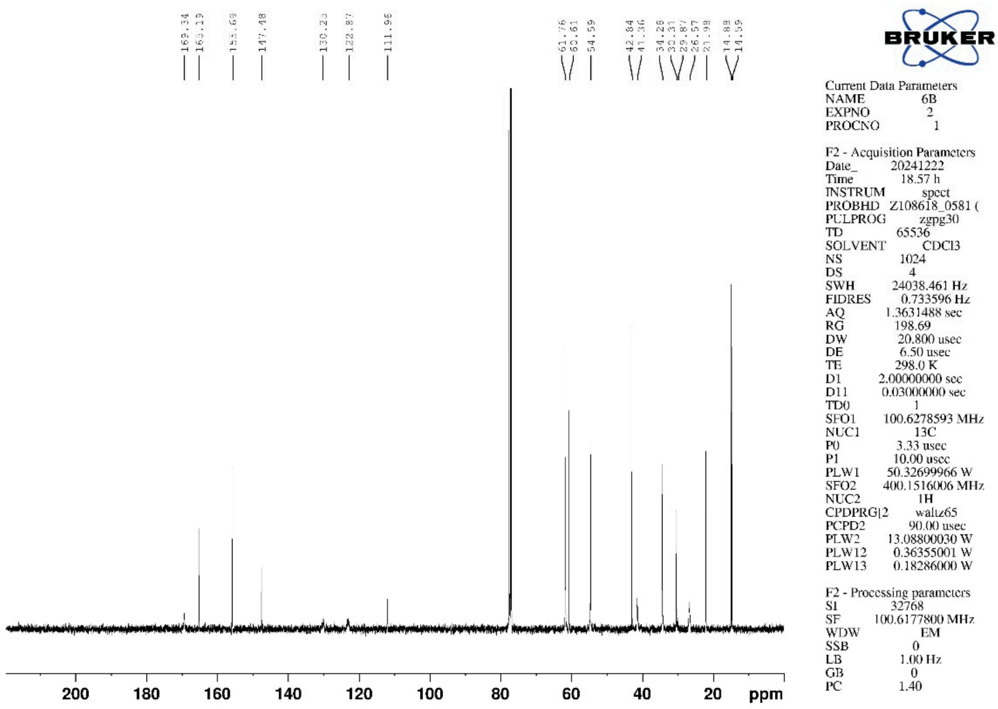

Figure **S7**. ^{13}C NMR of **6b**

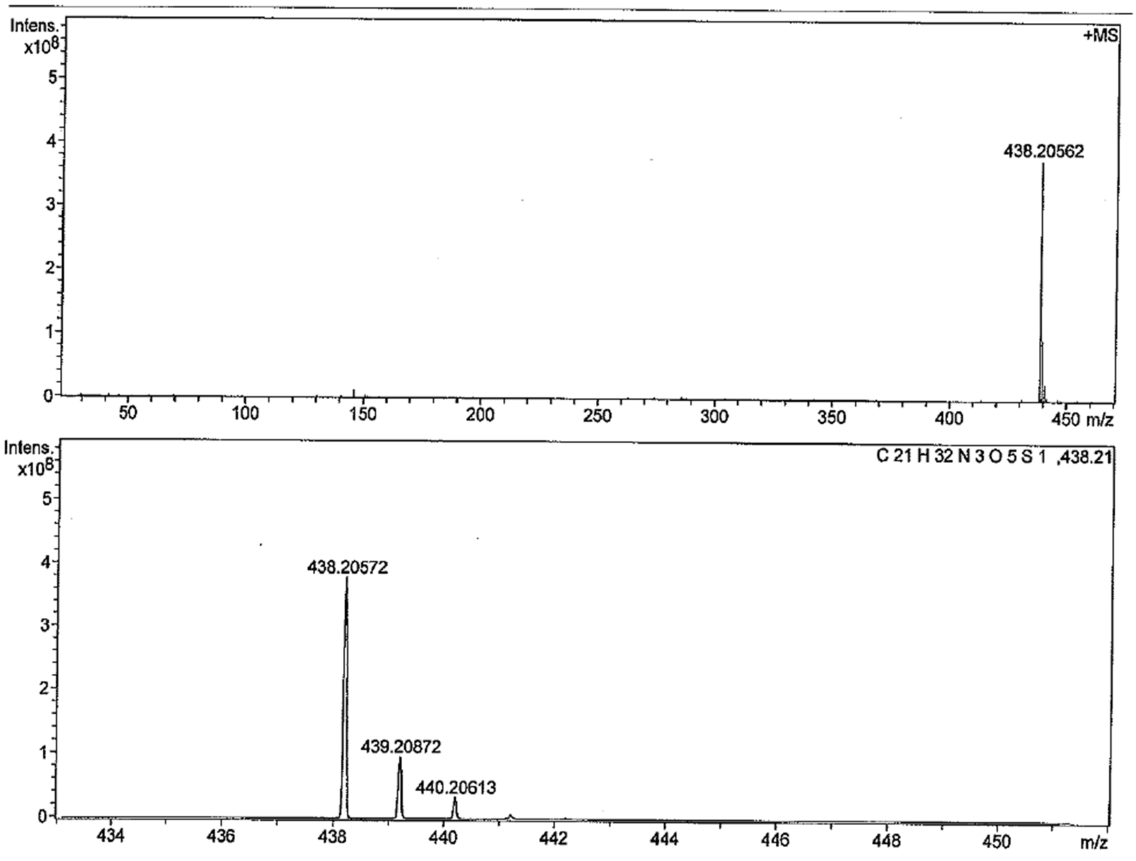

Sum Formula	Sigma	m/z	Err [ppm]	Mean Err [ppm]	Err [mDa]	rdb	N Rule	e⁻
C 21 H 32 N 3 O 5 S 1	0.009	438.20572	0.22	0.25	0.10	7.50	ok	even

Figure **S8**. HRMS of **6b**

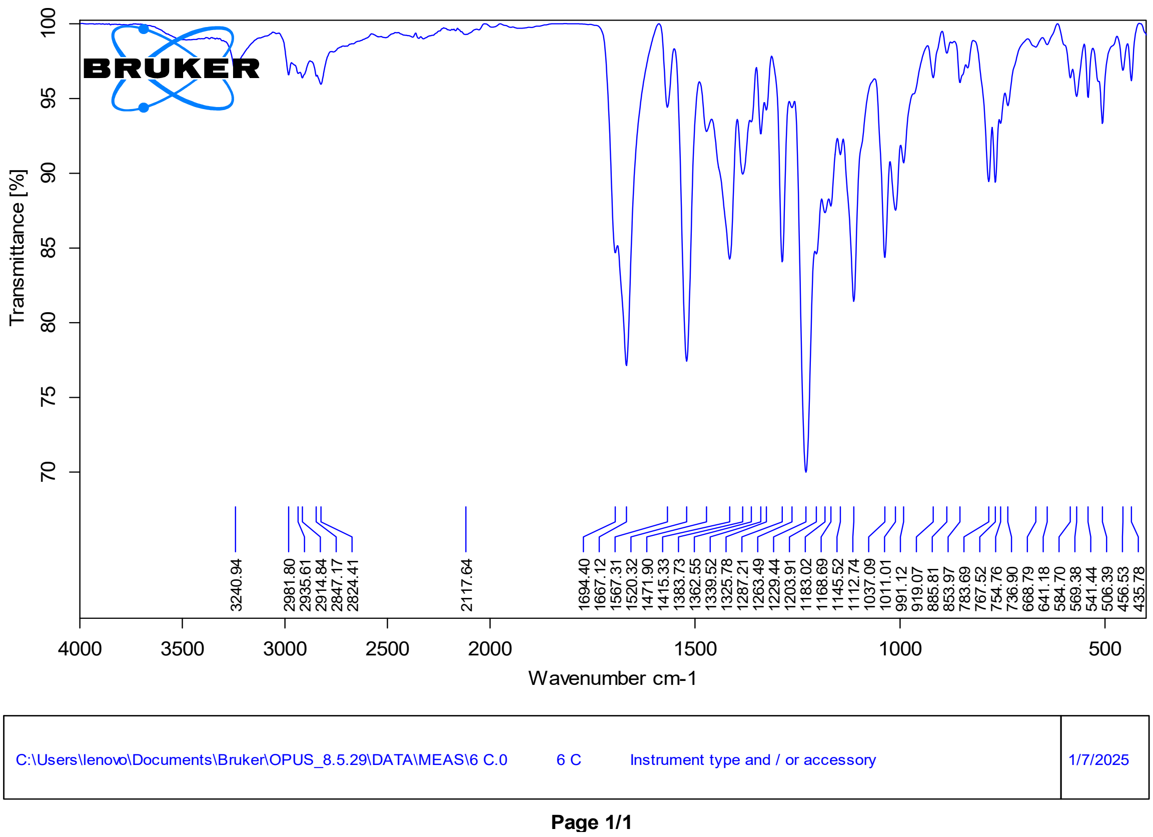

Figure **S9**. FT-IR of **6c**

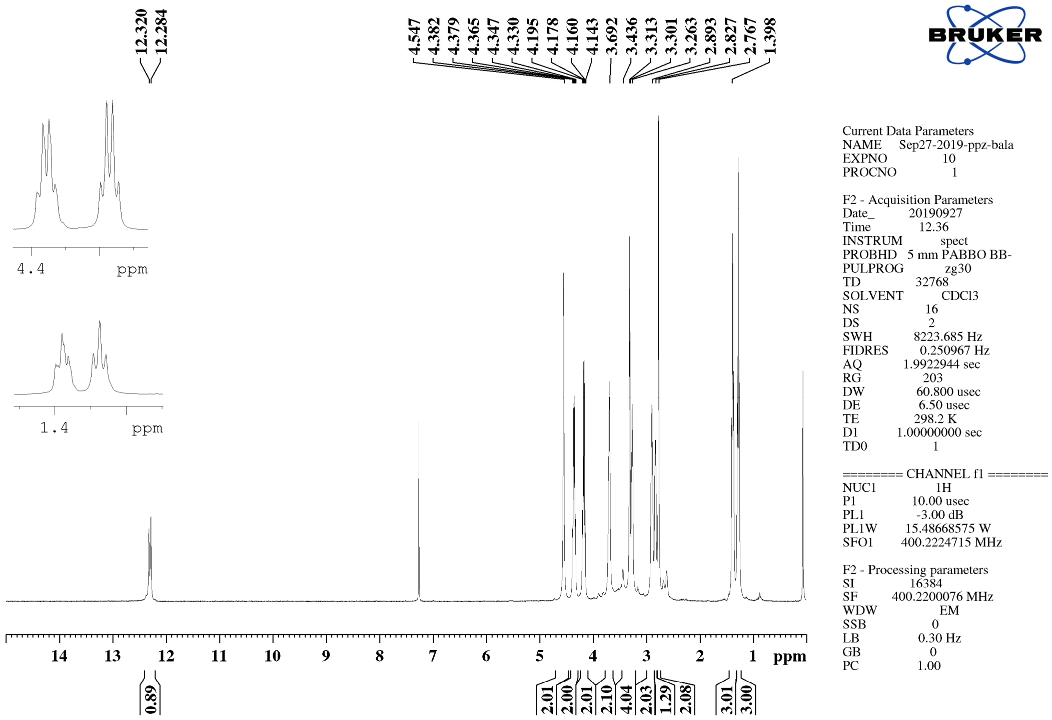

Figure **S10**. ^{1}H NMR of **6c**

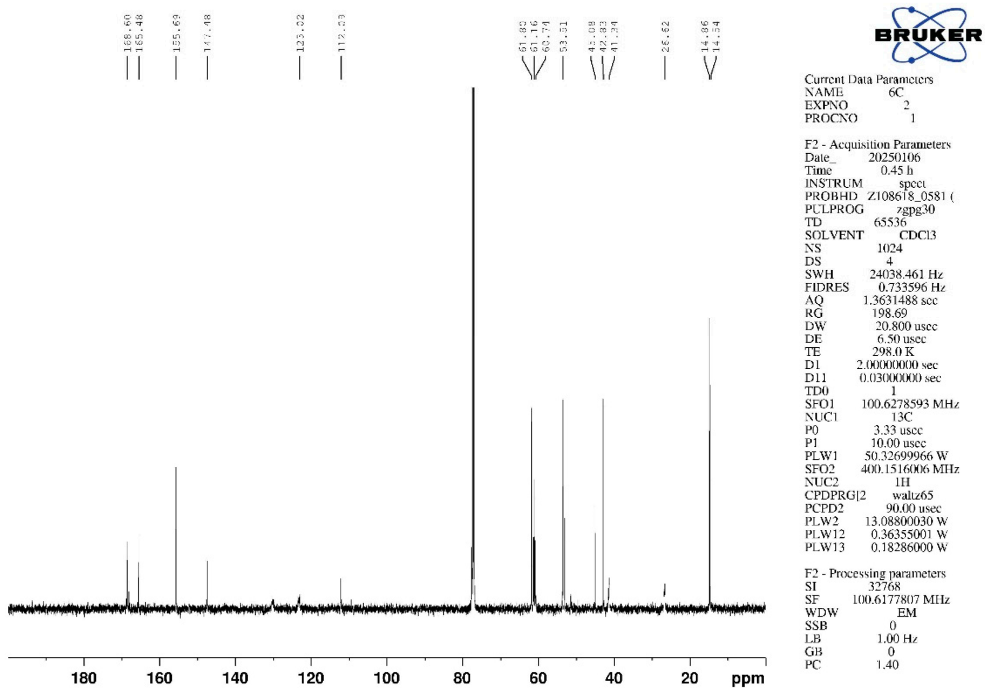

Figure **S11**. ^{13}C NMR of **6c**

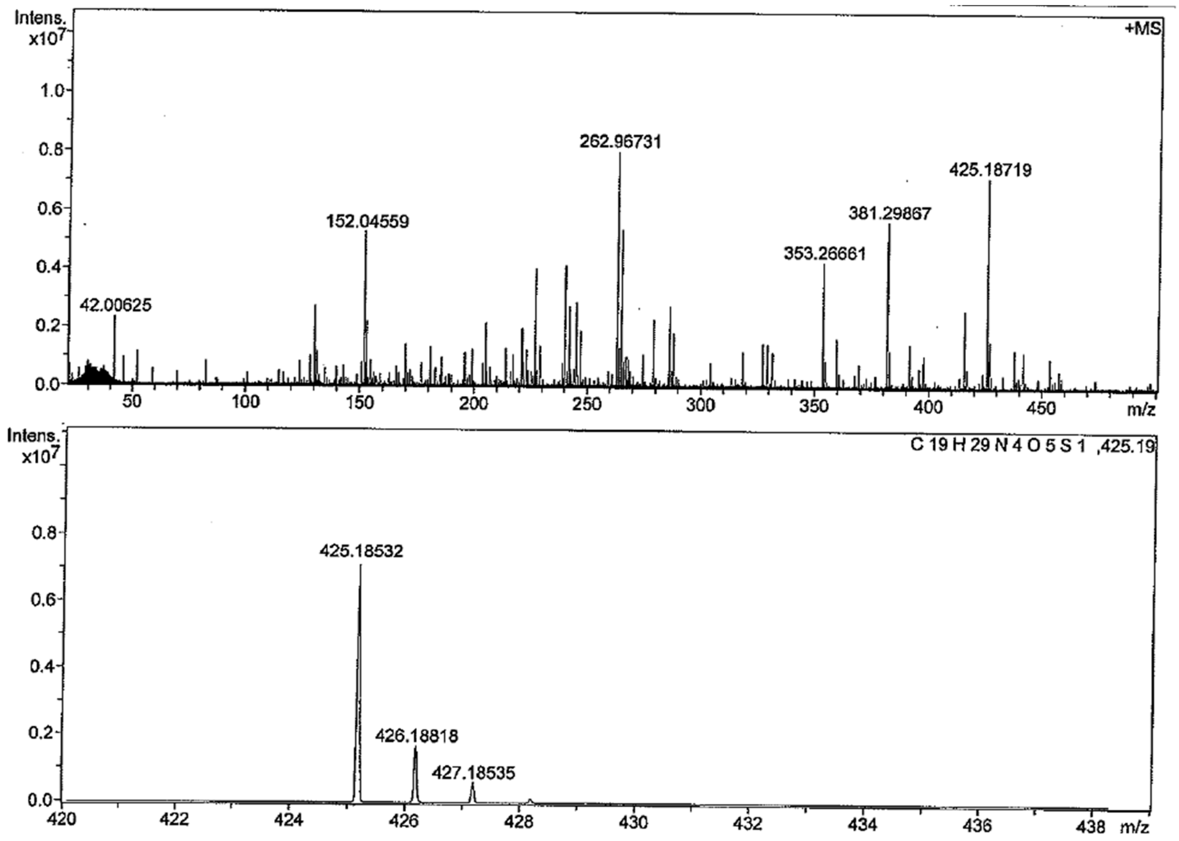

Sum Formula	Sigma	m/z	Err [ppm]	Mean Err [ppm]	Err [mDa]	rdb	N Rule	e⁻
C 19 H 29 N 4 O 5 S 1	0.012	425.18532	-4.40	-3.78	-1.87	7.50	ok	even

Figure **S12**. HRMS of **6c**

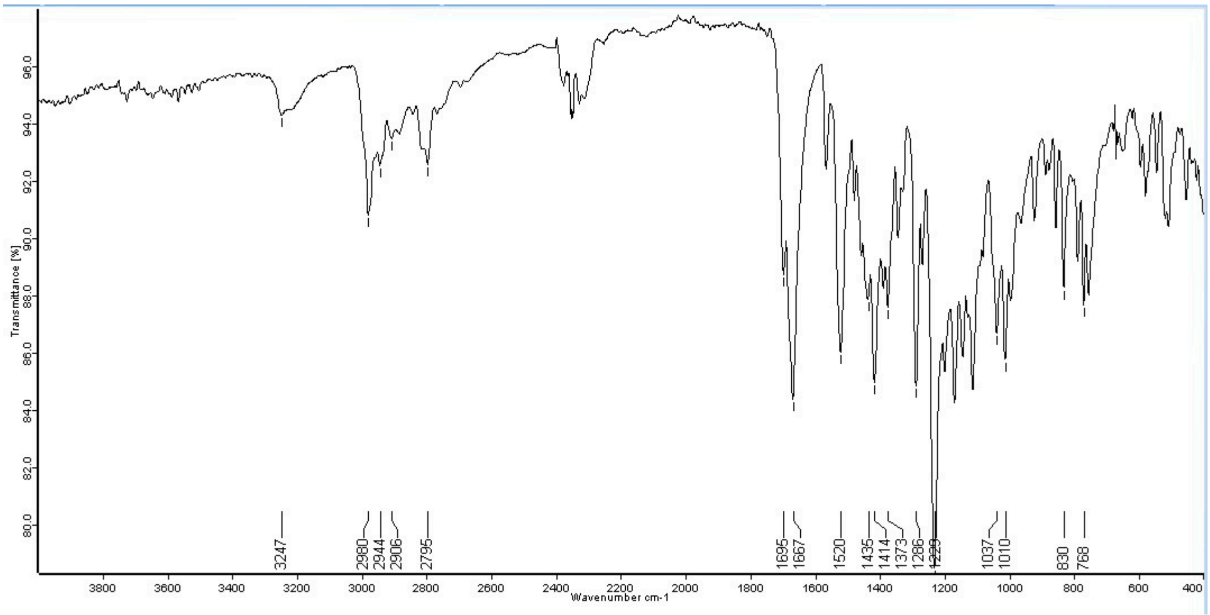

Figure **S13**. FT-IR of **6d**

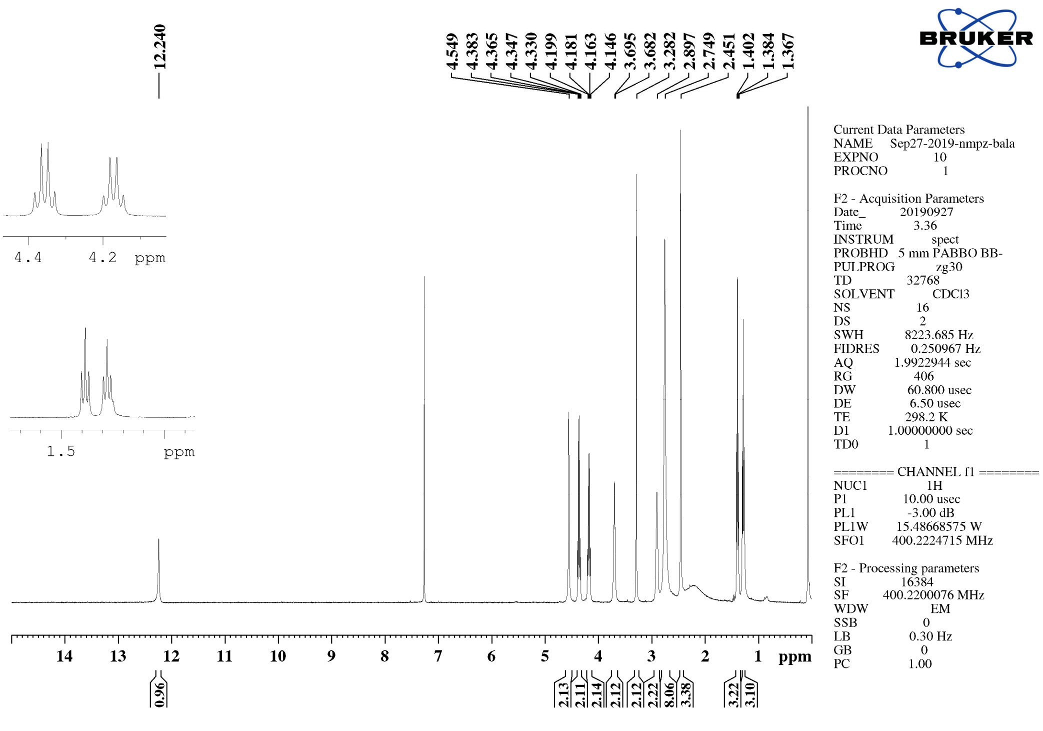

Figure **S14**. ^{1}H NMR of **6d**

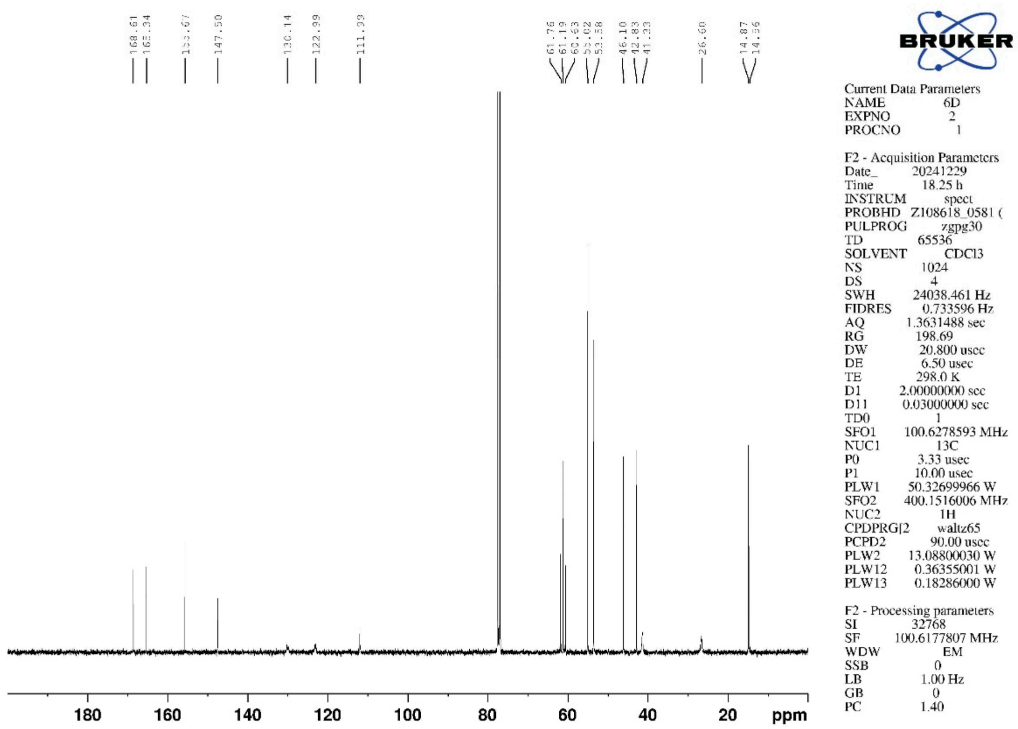

Figure **S15**. ^{13}C NMR of **6d**

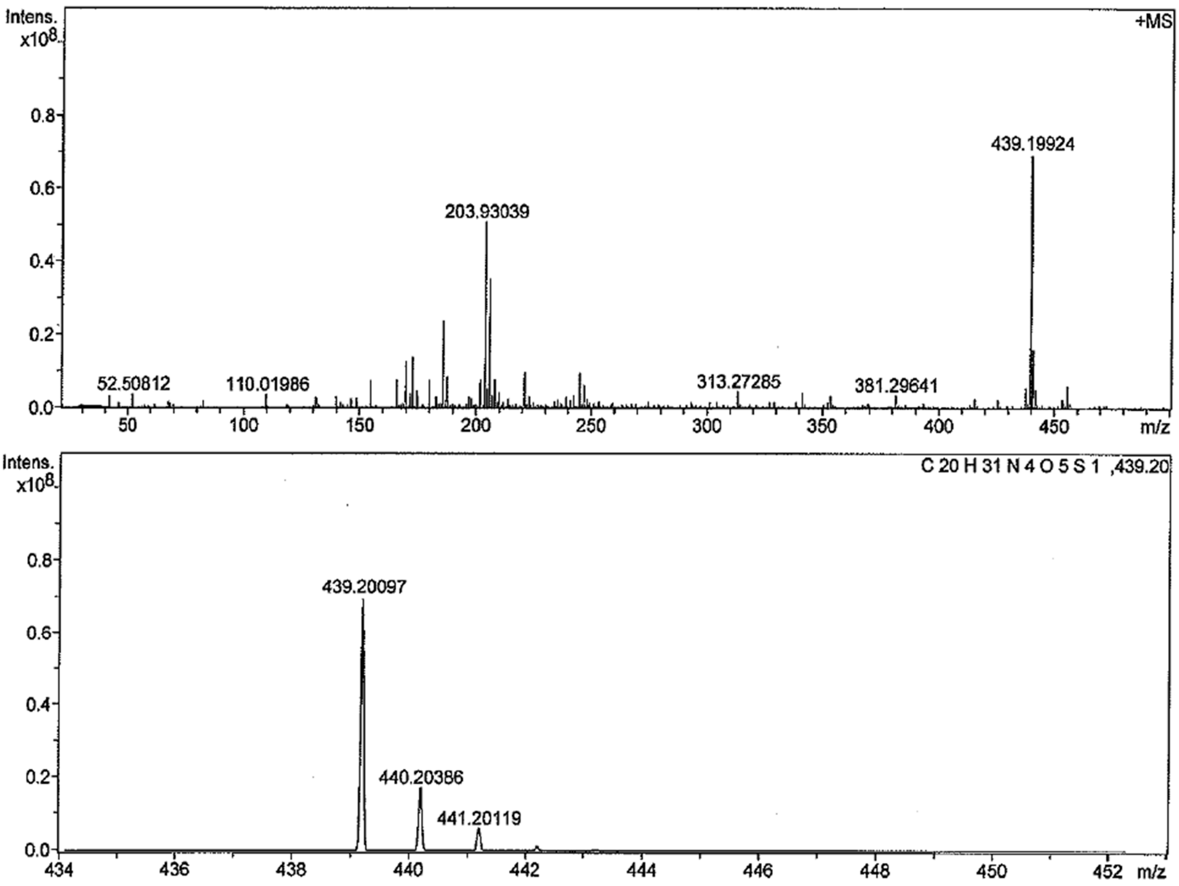

Sum Formula	Sigma	m/z	Err [ppm]	Mean Err [ppm]	Err [mDa]	rdb	N Rule	e⁻
C 20 H 31 N 4 O 5 S 1	0.009	439.20097	3.94	4.04	1.73	7.50	ok	even

Figure **S16**. HRMS of **6d**

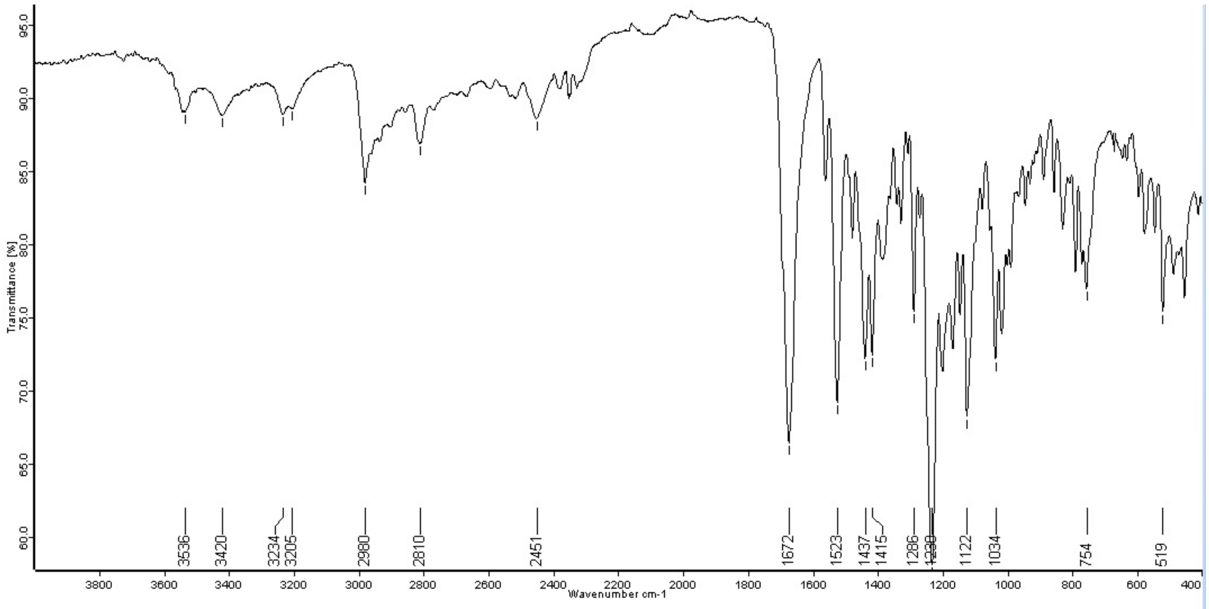

Figure **S17**. FT-IR of **6e**

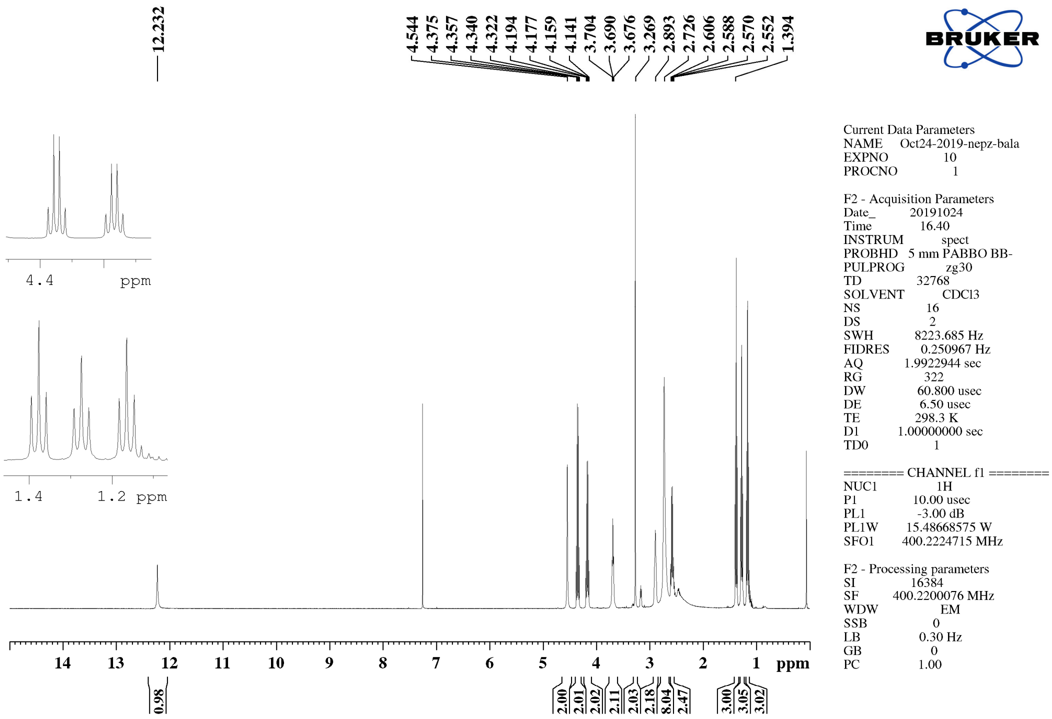

Figure **S18**. ^{1}H NMR of **6e**

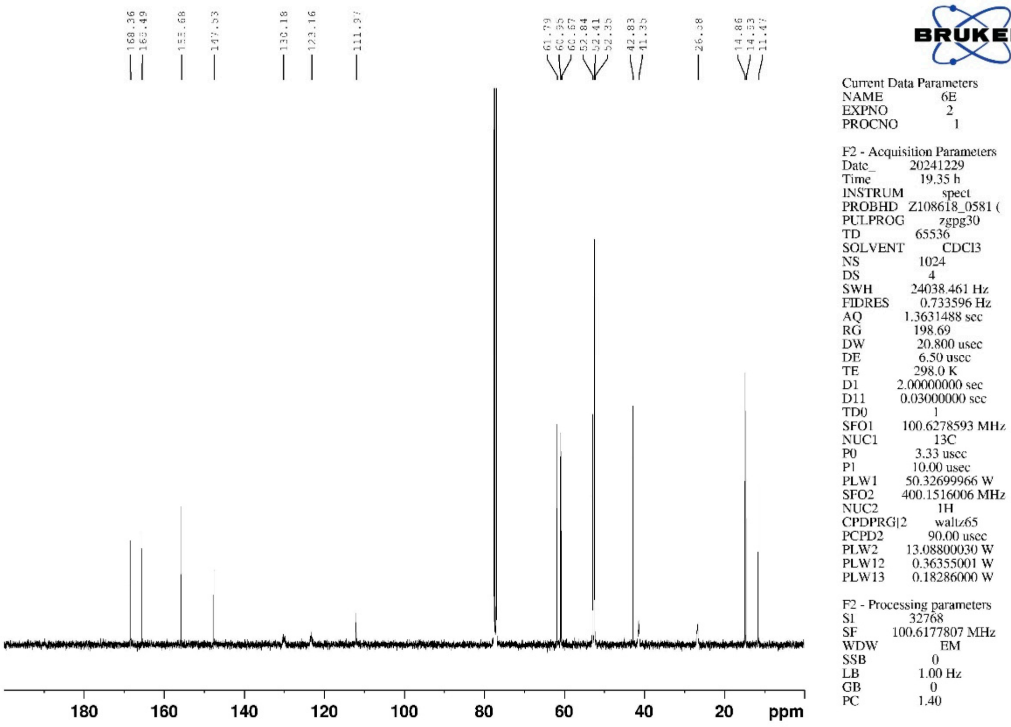

Figure **S19**. ^{13}C NMR of **6e**

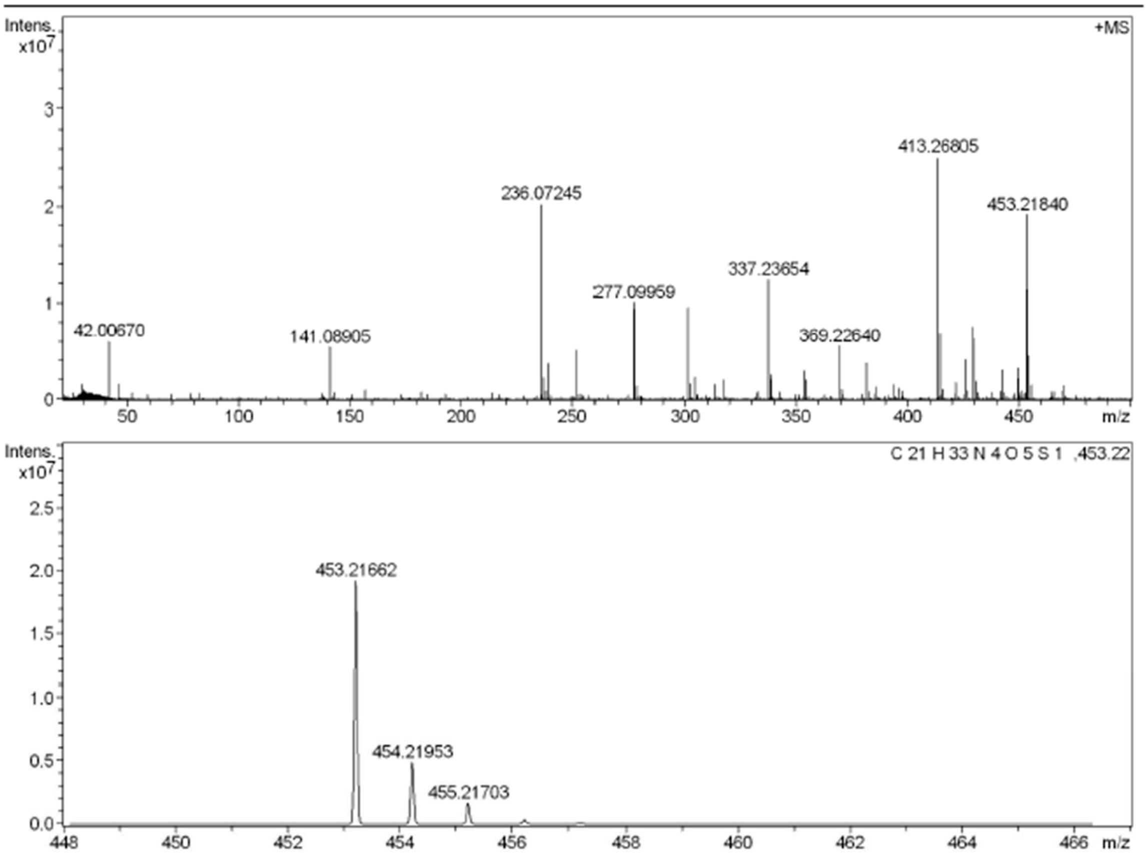

Sum Formula	Sigma	m/z	Err [ppm]	Mean Err [ppm]	Err [mDa]	rdb	N Rule	e⁻
C 21 H 33 N 4 O 5 S 1	0.008	453.21662	-3.92	-3.81	-1.78	7.50	ok	even

Bruker Daltonics DataAnalysis 3.4 printed: 11/25/2024 10:25:48 AM Page 1 of 1

Figure **S20**. HRMS of **6e**

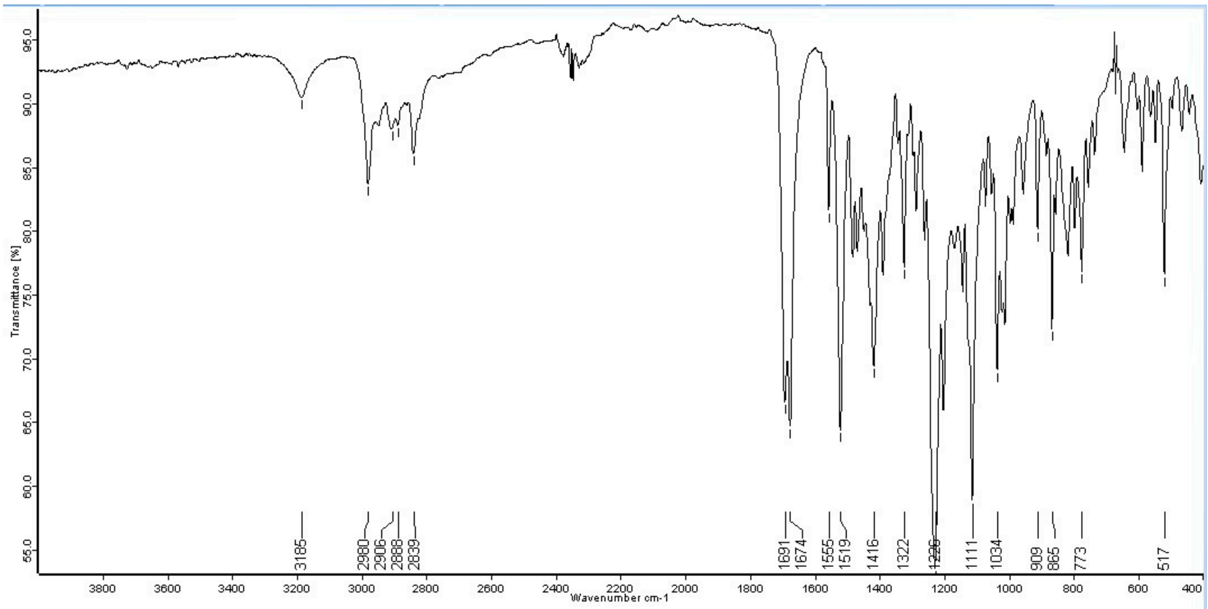

Figure **S21**. FT-IR of **6f**

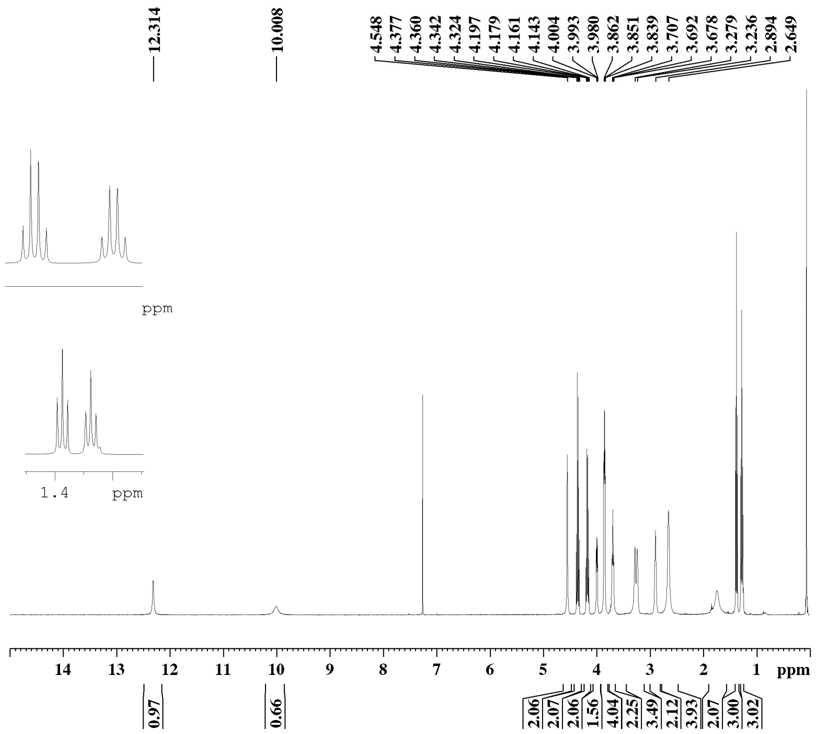

Figure **S22**. ^{1}H NMR of **6f**

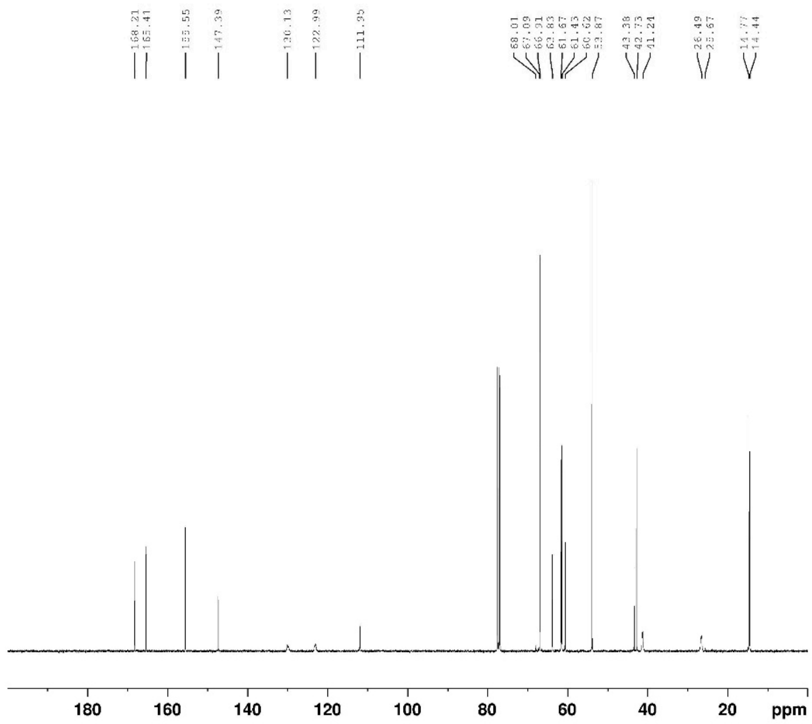

Figure **S23**. ^{13}C NMR of **6f**

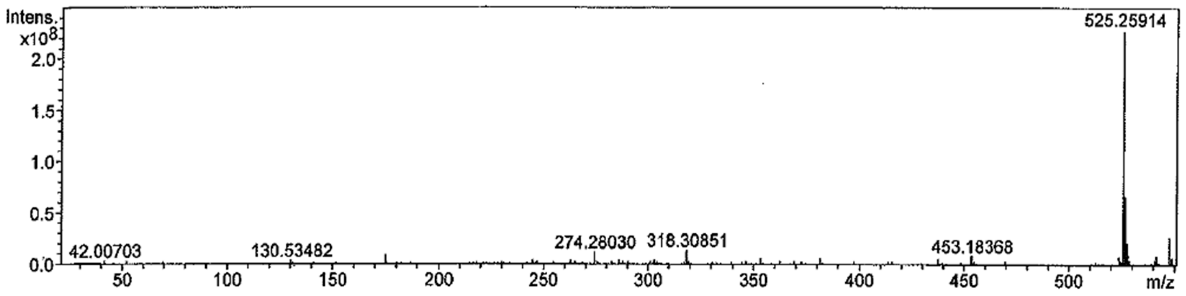

Figure **S24**. HRMS of **6f**

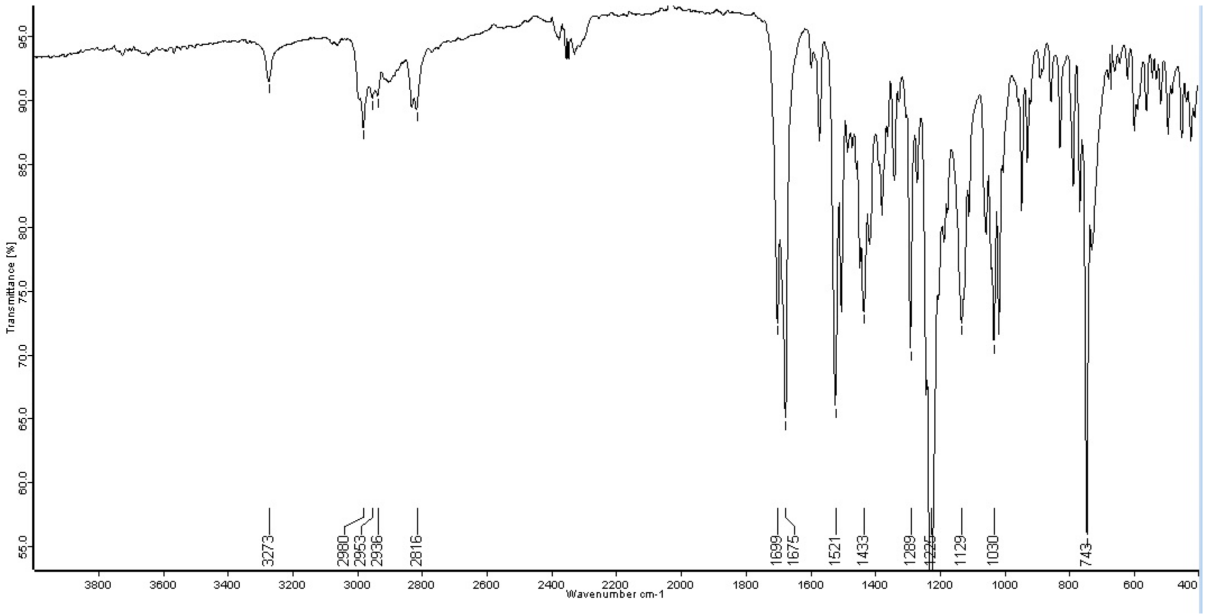

Figure **S25**. FT-IR of **6g**

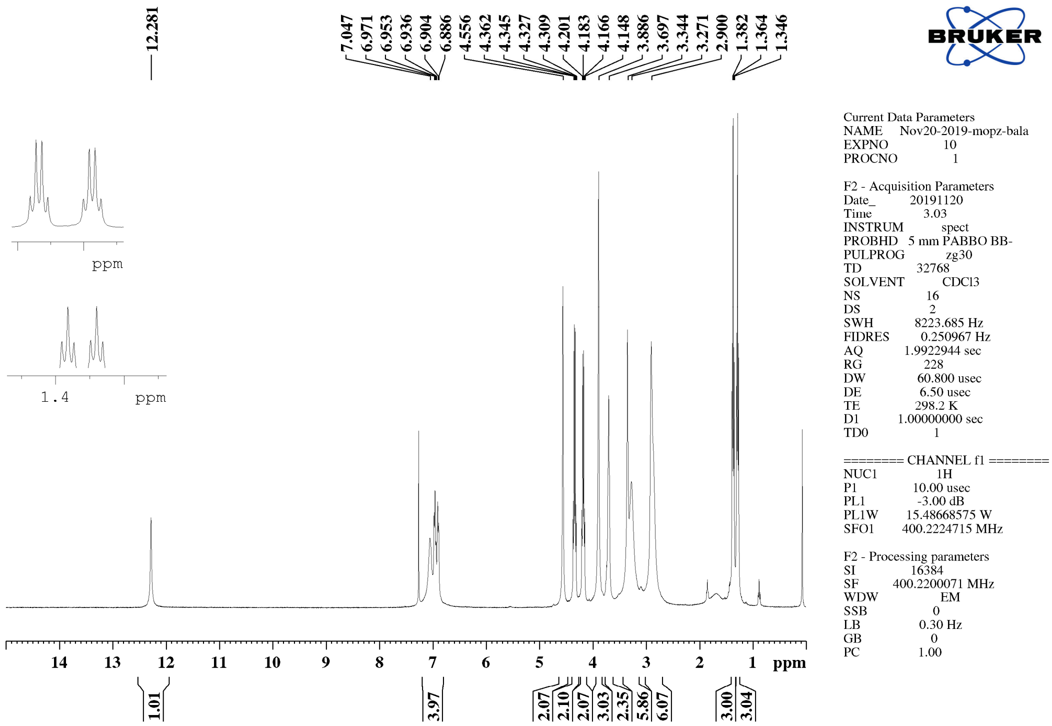

Figure **S26**. ^{1}H NMR of **6g**

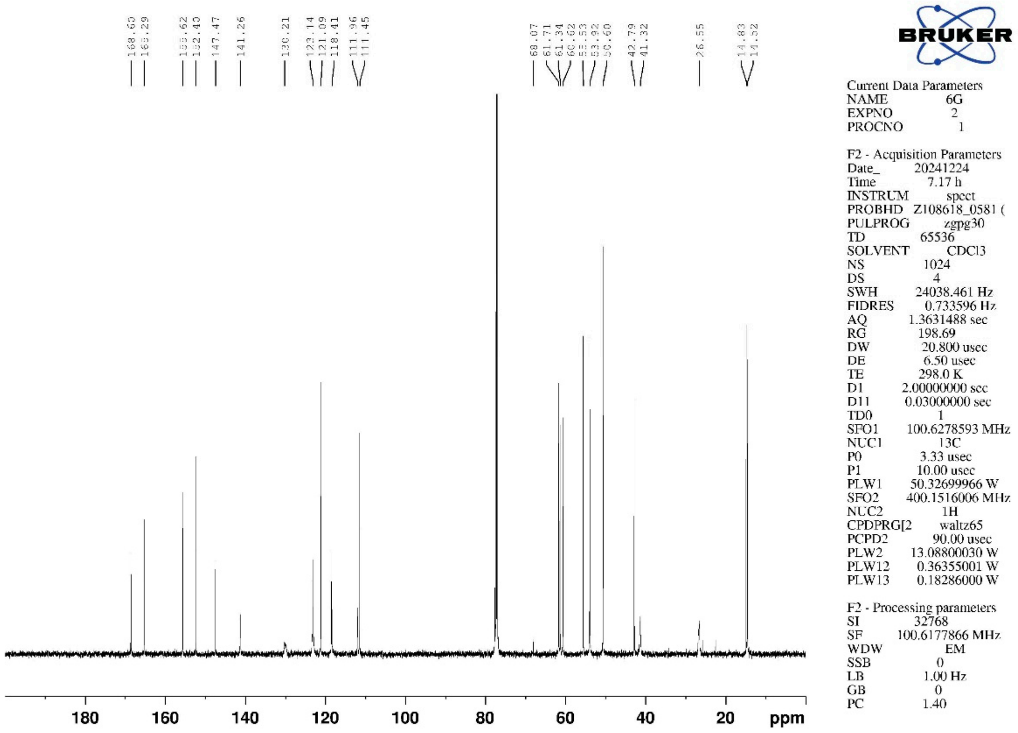

Figure **S27**. ^{13}C NMR of **6g**

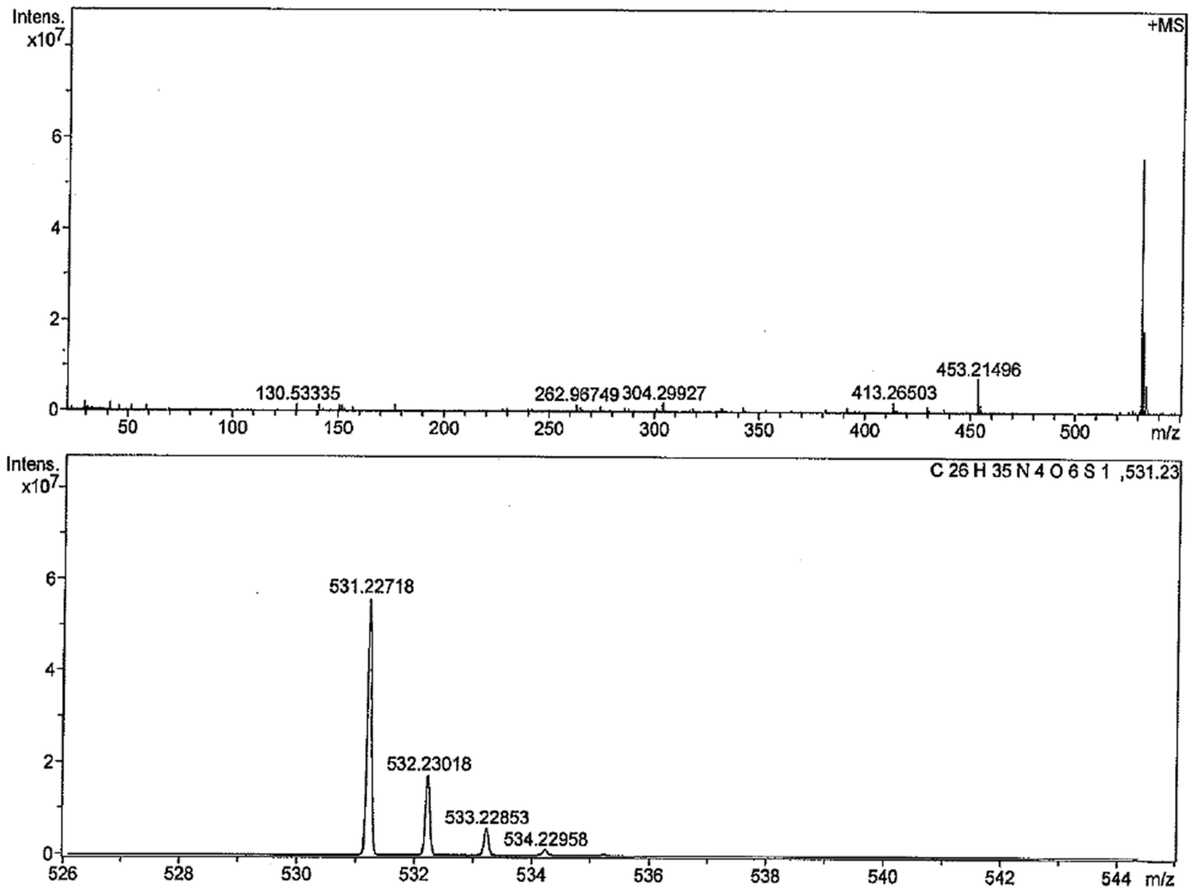

Sum Formula	Sigma	m/z	Err [ppm]	Mean Err [ppm]	Err [mDa]	rdb	N Rule	e⁻
C 26 H 35 N 4 O 6 S 1	0.008	531.22718	2.28	2.57	1.21	11.50	ok	even

Figure **S28**. HRMS of **6g**

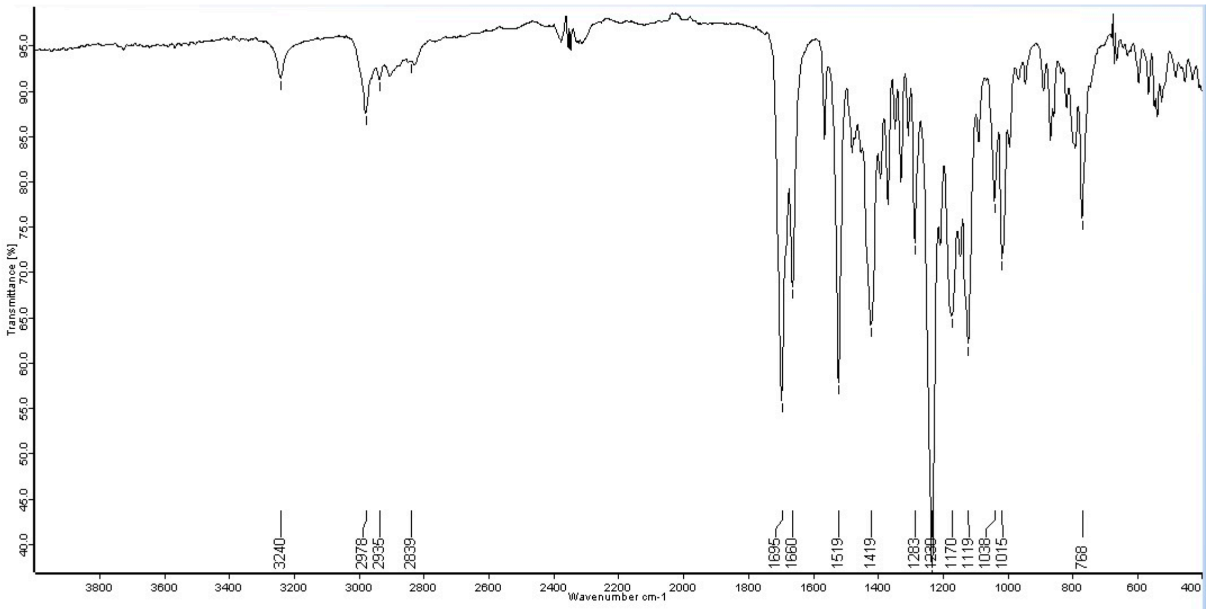

Figure **S29**. FT-IR of **6h**

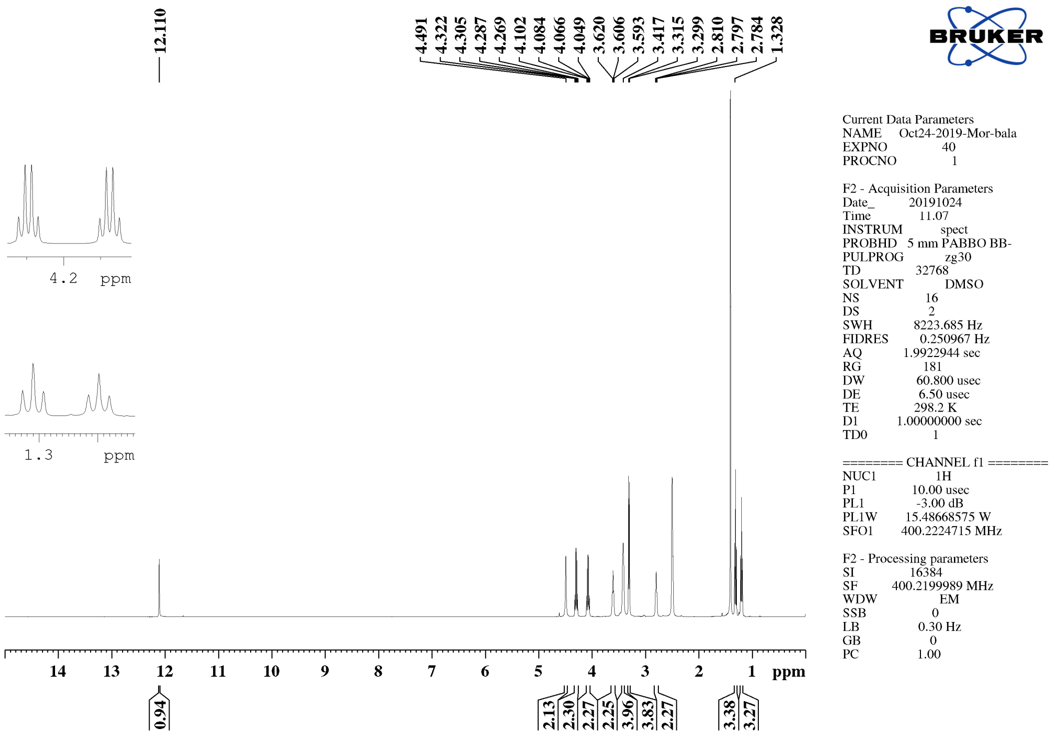

Figure **S30**. ^{1}H NMR of **6h**

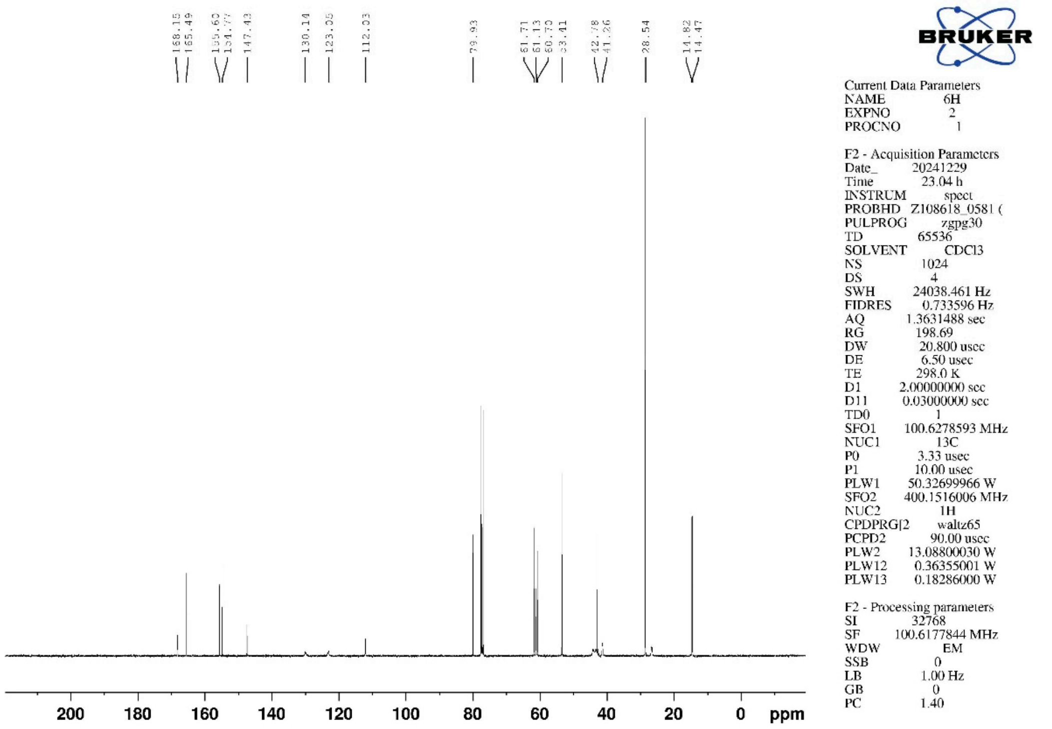

Figure **S31**. ^{13}C NMR of **6h**

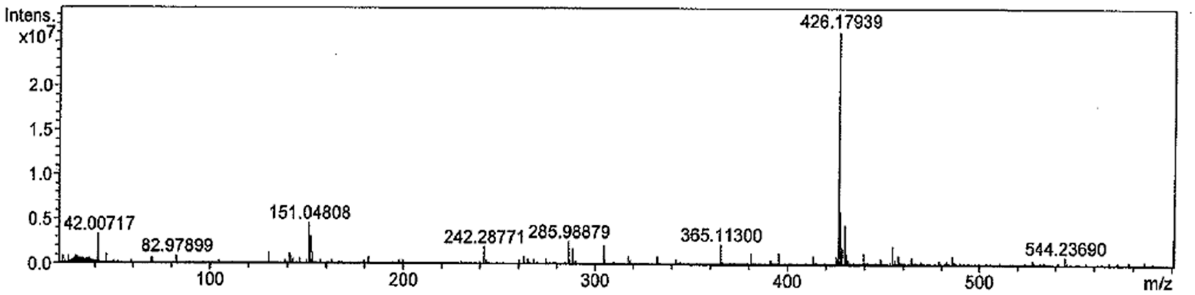

Figure **S32**. HRMS of **6h**

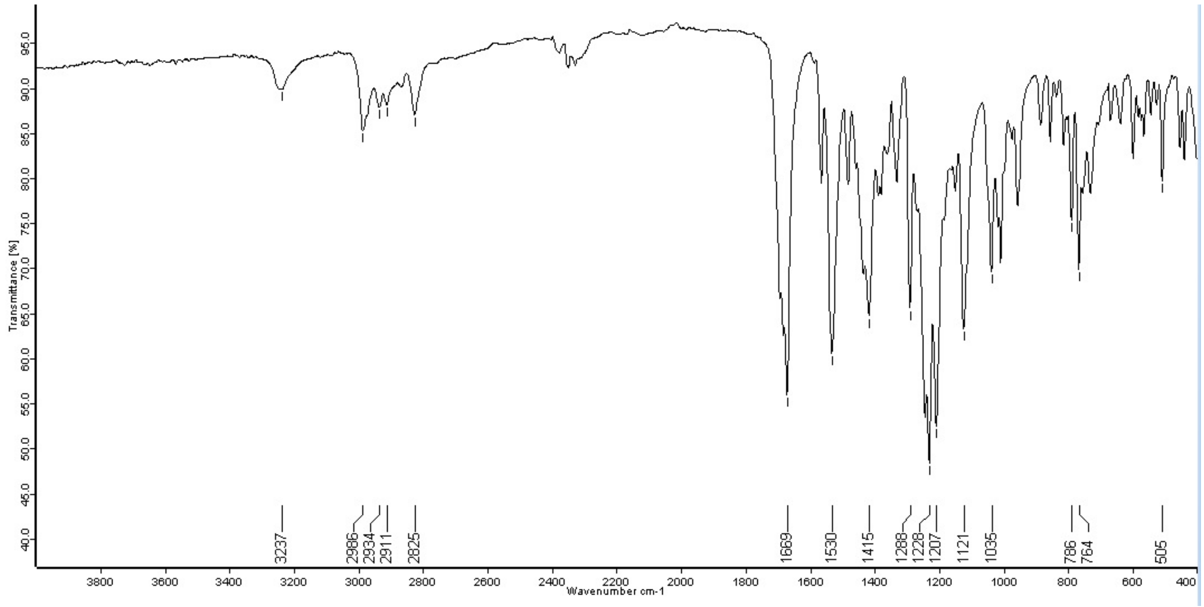

Figure **S33**. FT-IR of **6i**

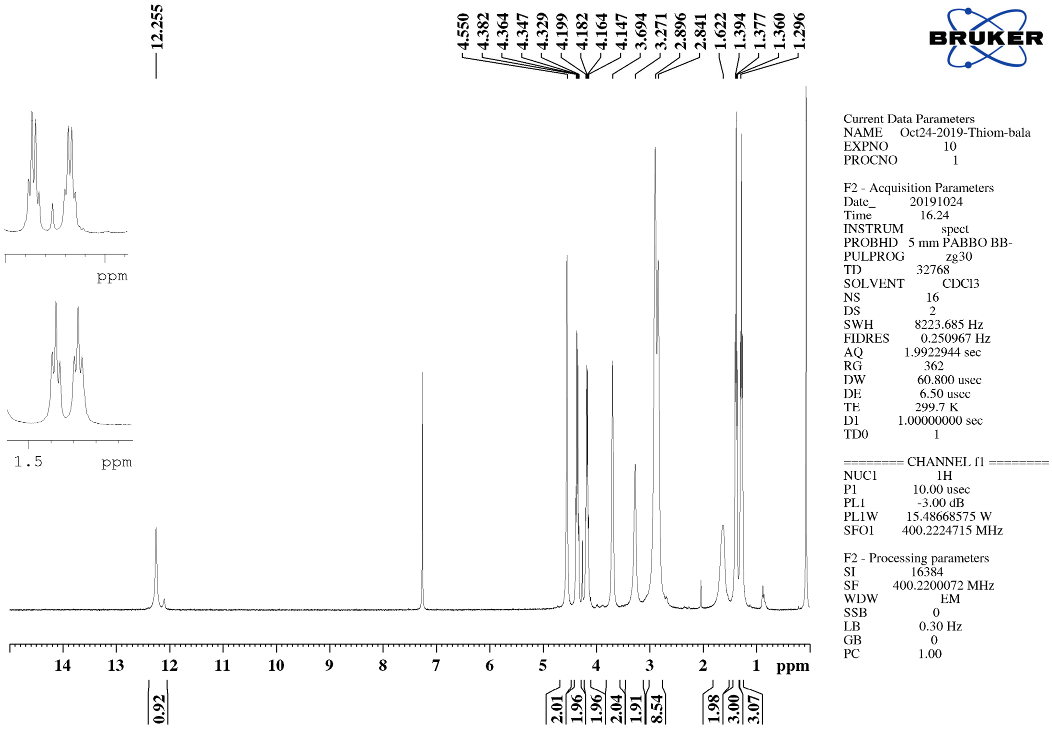

Figure **S34**. ^{1}H NMR of **6i**

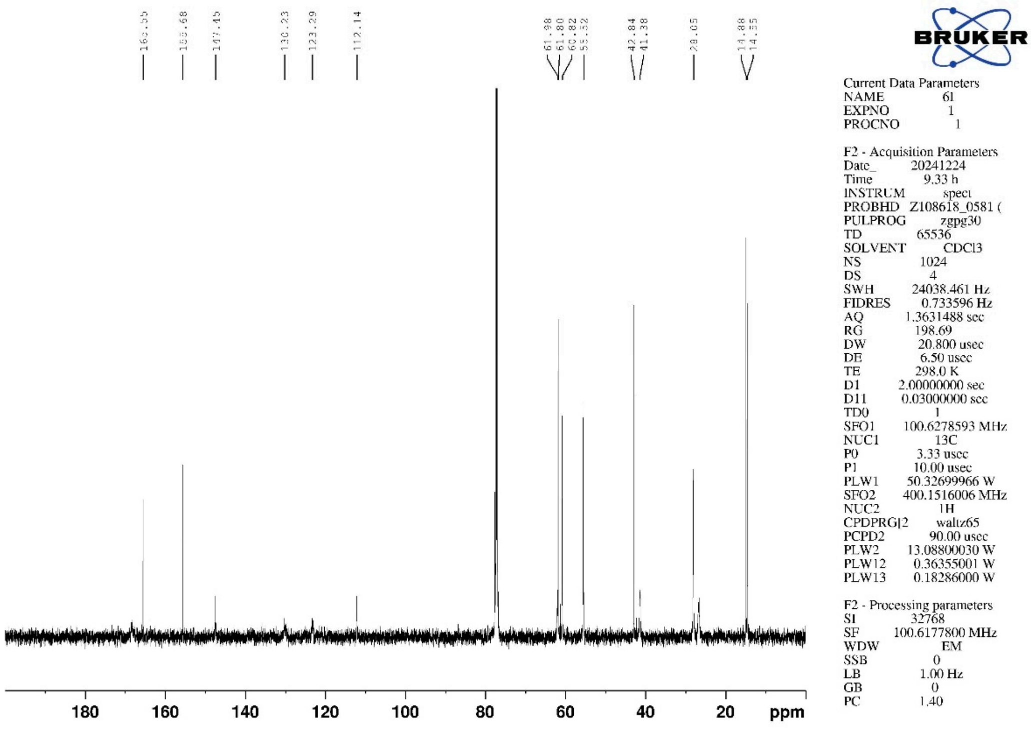

Figure **S35**. ^{13}C NMR of **6i**

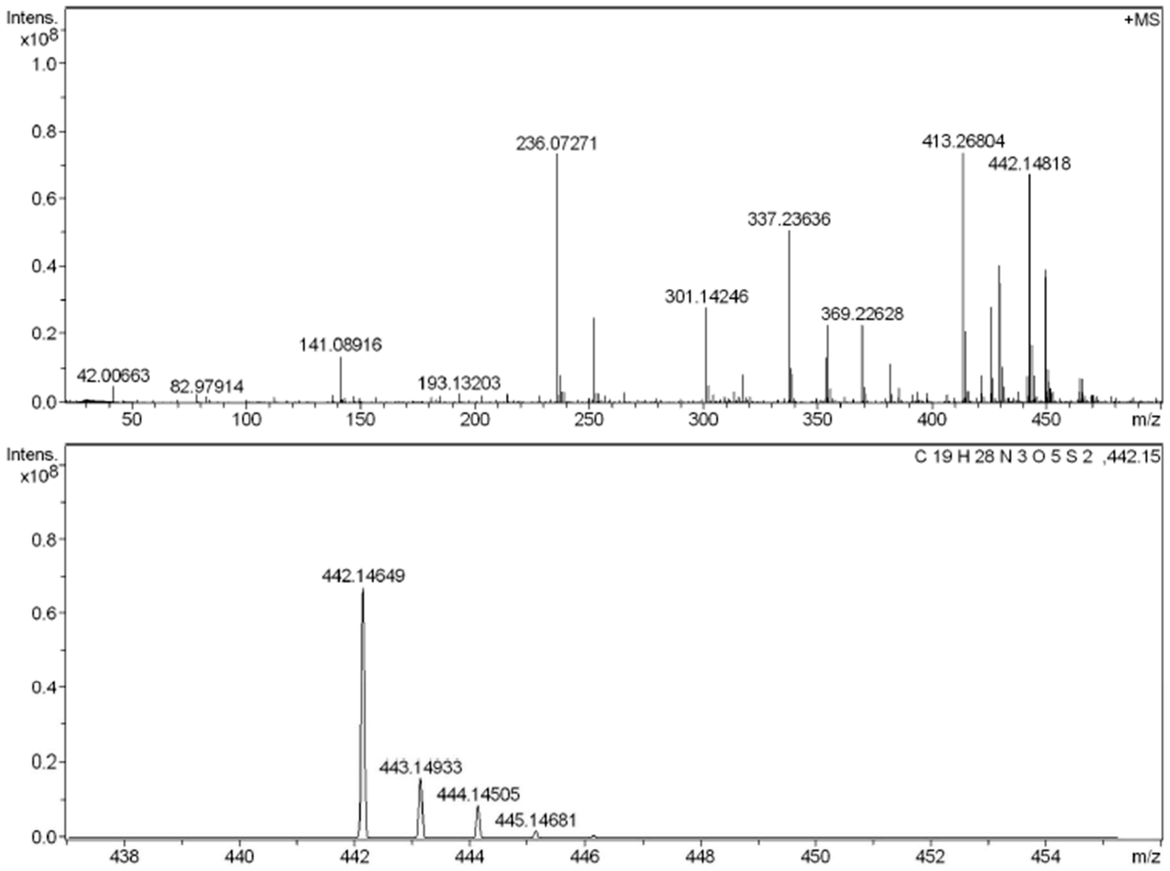

Sum Formula	Sigma	m/z	Err [ppm]	Mean Err [ppm]	Err [mDa]	rdb	N Rule	e⁻
C 19 H 28 N 3 O 5 S 2	0.008	442.14649	-3.84	-4.24	-1.70	7.50	ok	even

Bruker Daltonics DataAnalysis 3.4 printed: 11/25/2024 10:22:22 AM Page 1 of 1

Figure **S36**. HRMS of **6i**

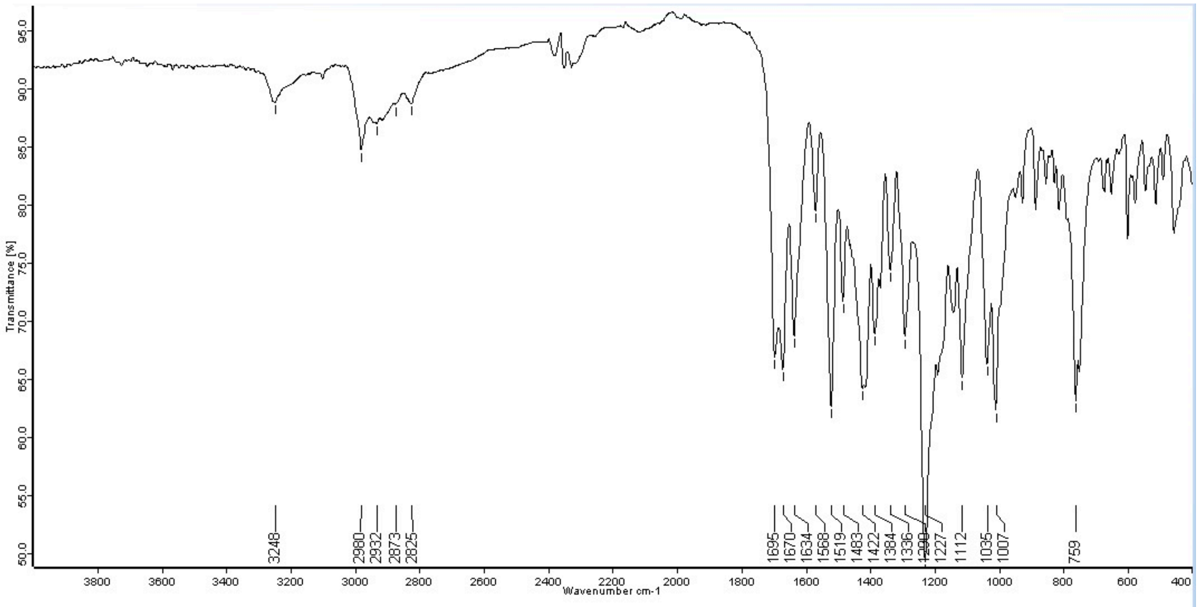

Figure **S37**. FT-IR of **6j**

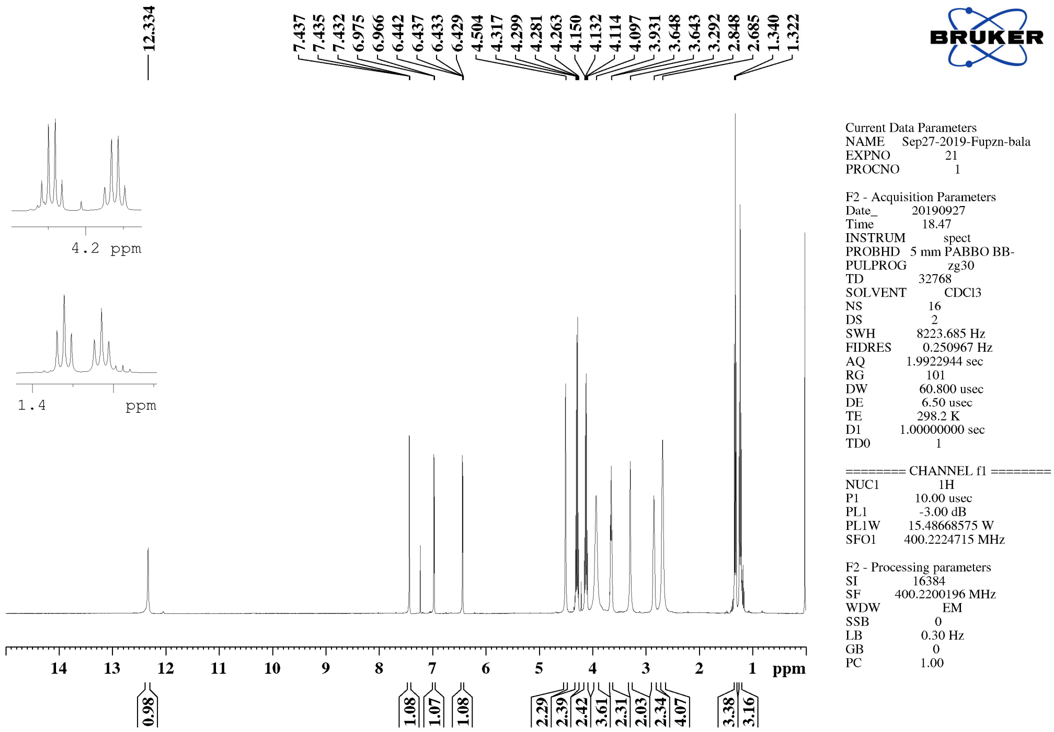

Figure **S38**. ^{1}H NMR of **6j**

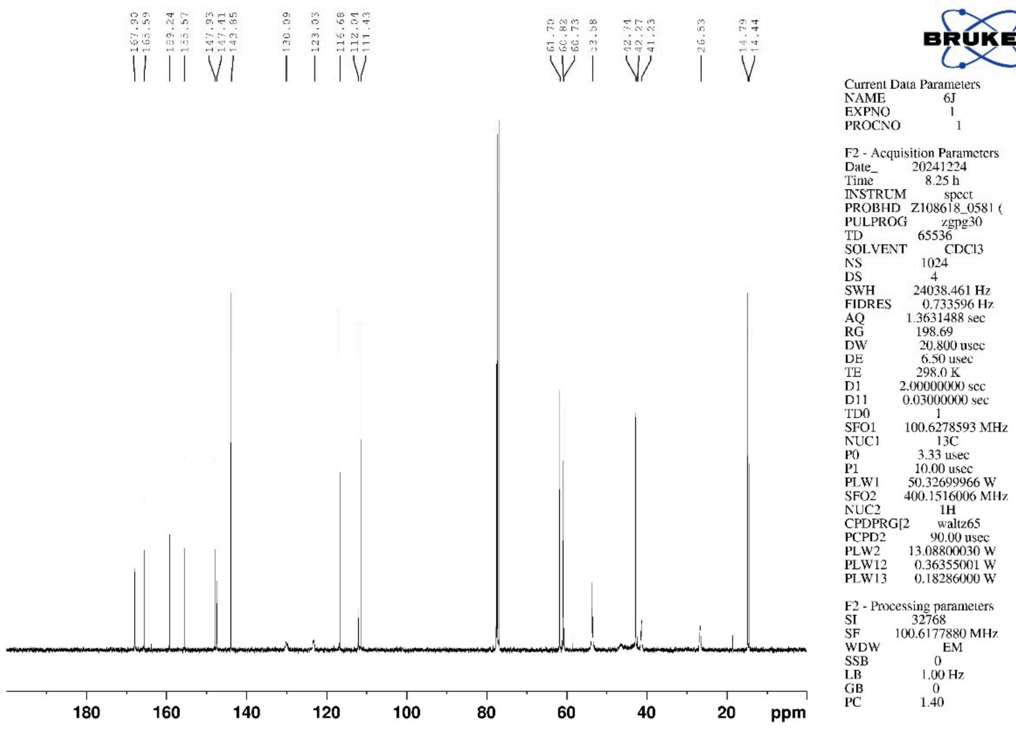

Figure **S39**. ^{13}C NMR of **6j**

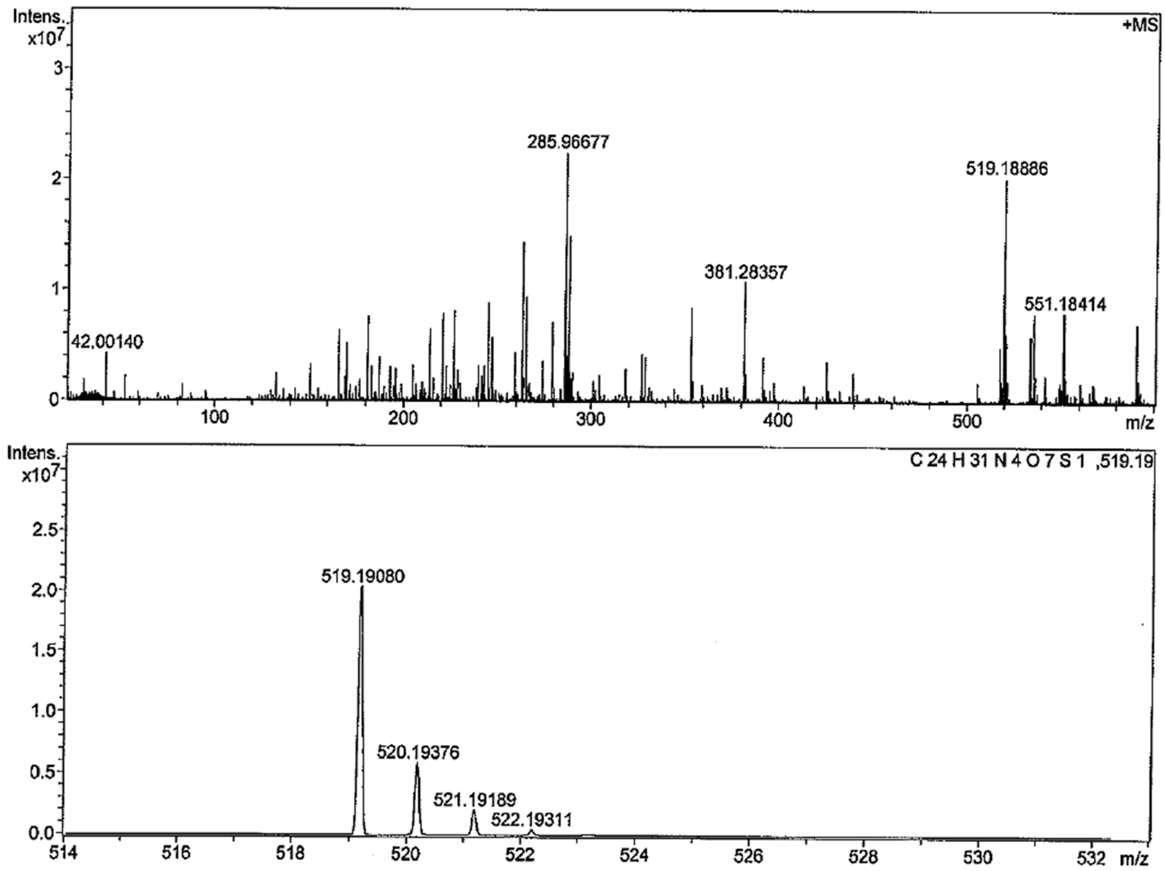

Sum Formula	Sigma	m/z	Err [ppm]	Mean Err [ppm]	Err [mDa]	rdb	N Rule	e⁻
C 24 H 31 N 4 O 7 S 1	0.010	519.19080	3.73	3.09	1.94	11.50	ok	even

Figure **S40**. HRMS of **6j**

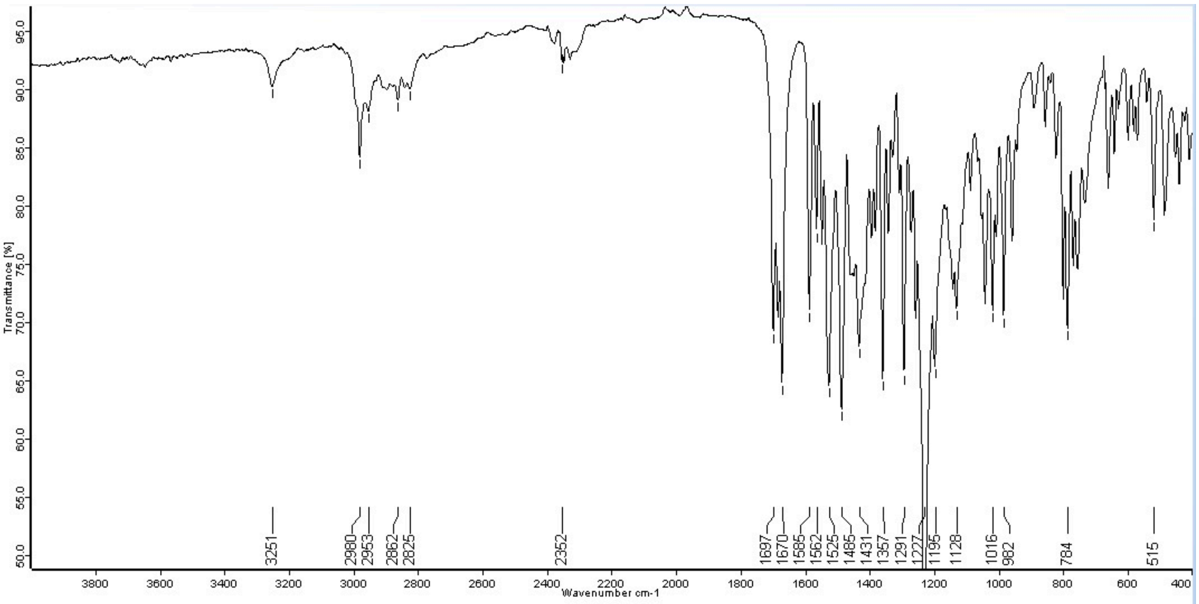

Figure **S41**. FT-IR of **6k**

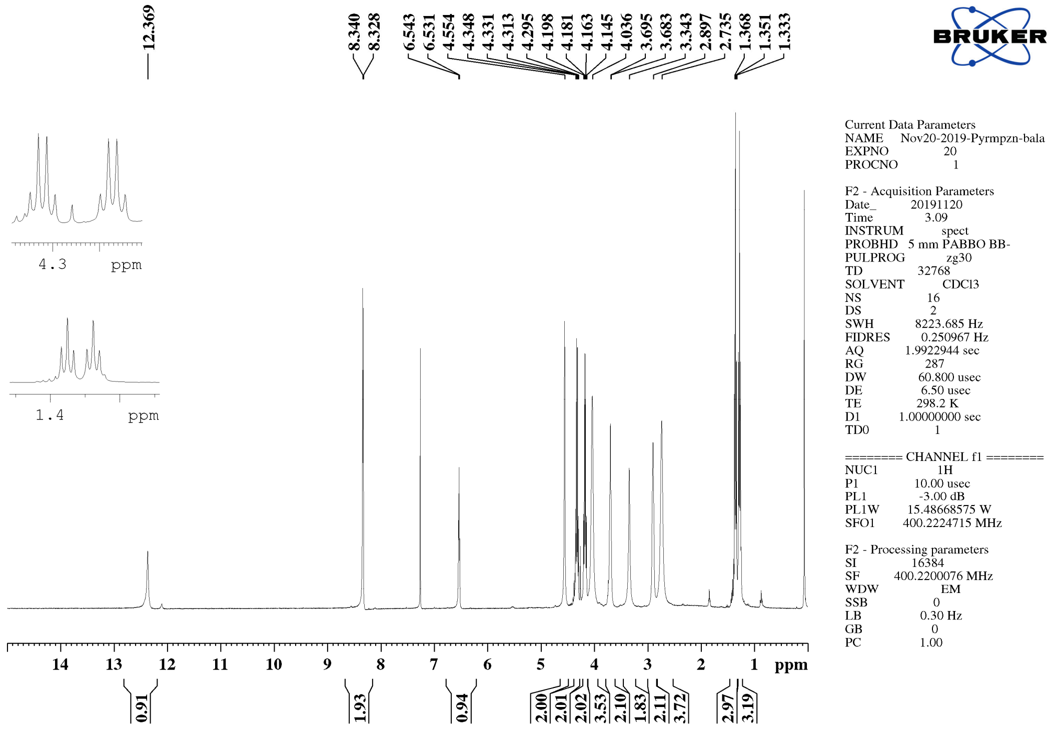

Figure **S42**. ^{1}H NMR of **6k**

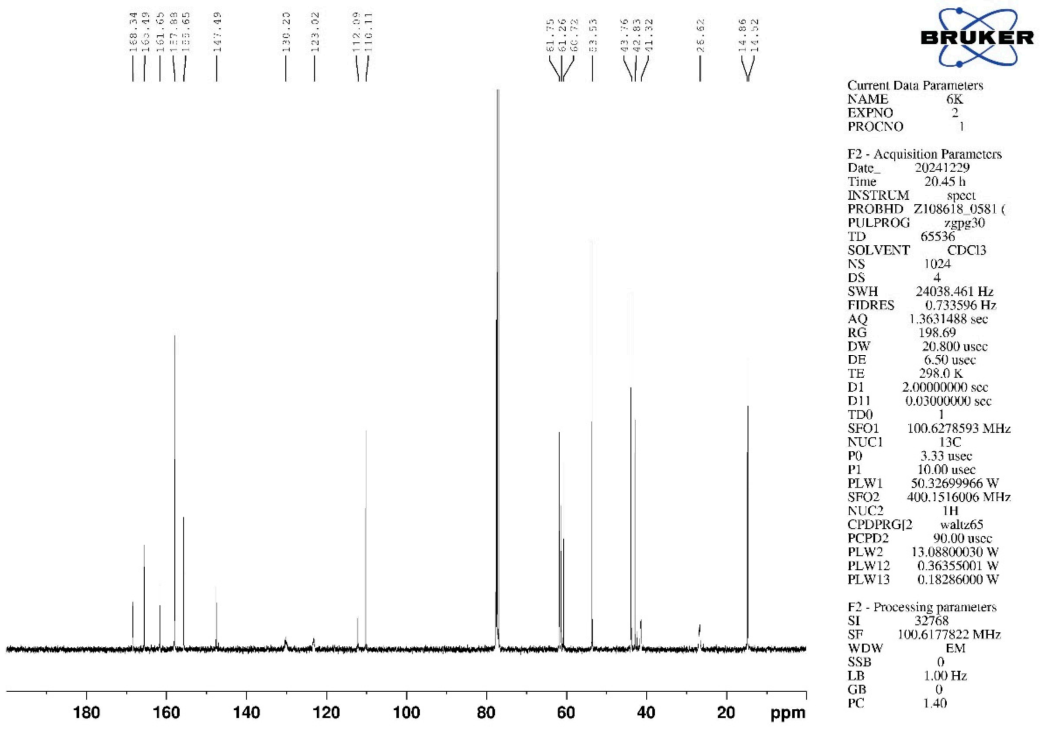

Figure **S43**. ^{13}C NMR of **6k**

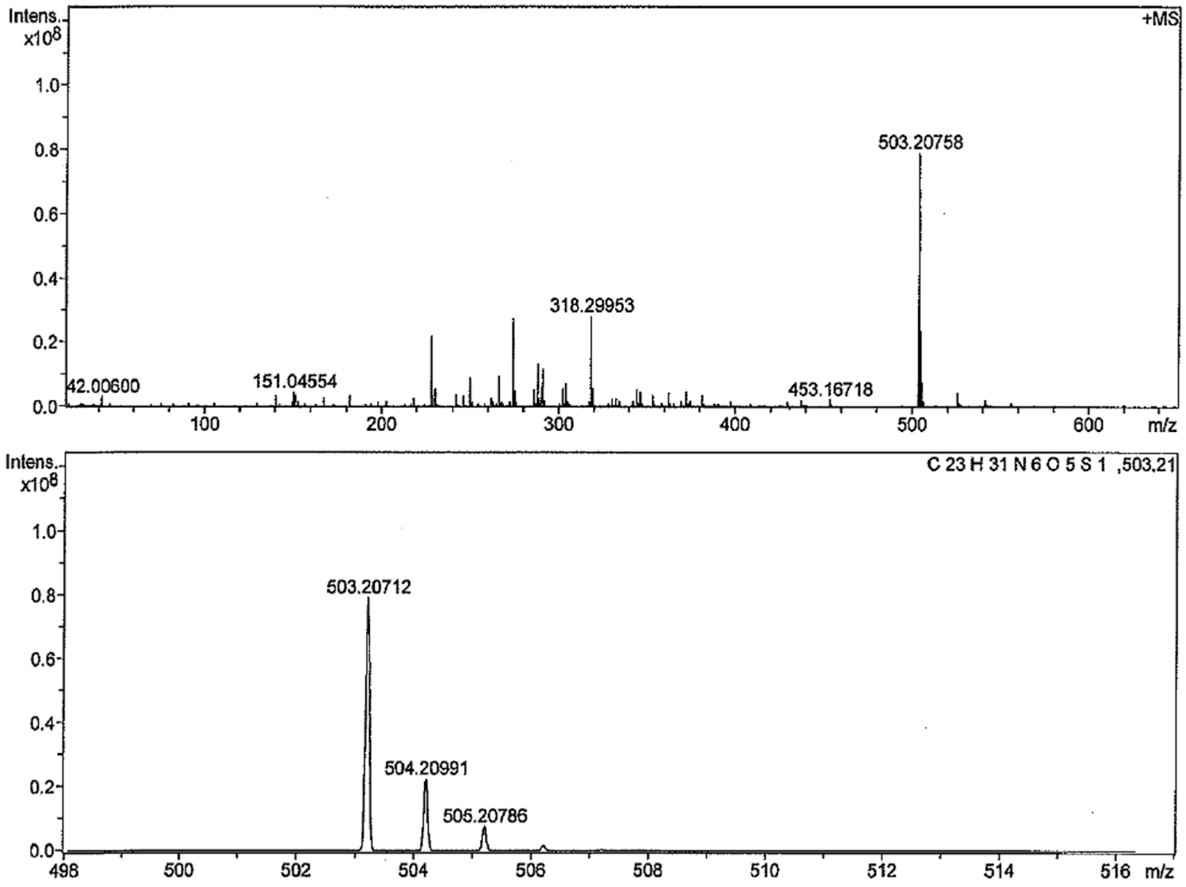

Sum Formula	Sigma	m/z	Err [ppm]	Mean Err [ppm]	Err [mDa]	rdb	N Rule	e⁻
C 23 H 31 N 6 O 5 S 1	0.008	503.20712	-0.91	-1.16	-0.46	11.50	ok	even

Figure **S44**. HRMS of **6k**

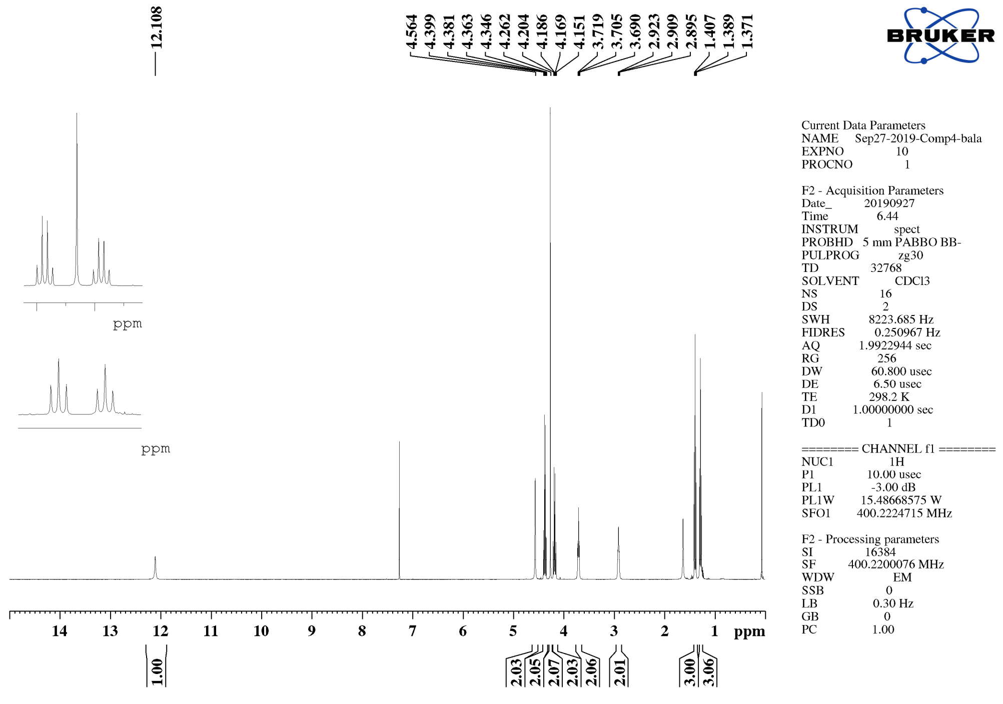

Figure **S45**. ^{1}H NMR of **4**

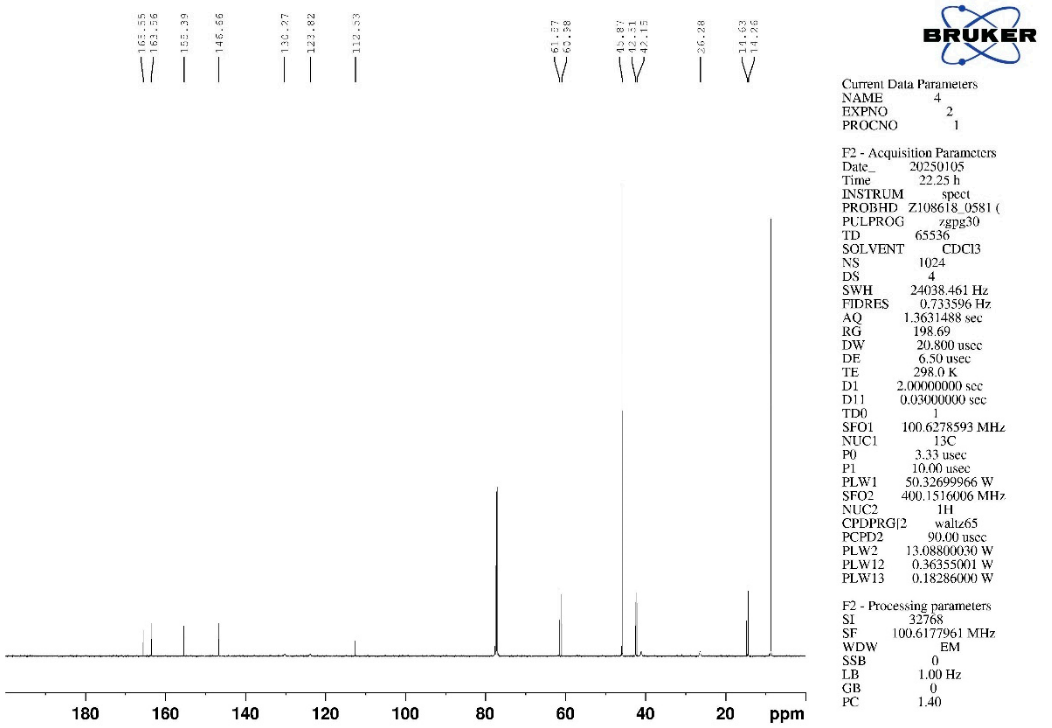

Figure **S46**. ^{13}C NMR of **4**

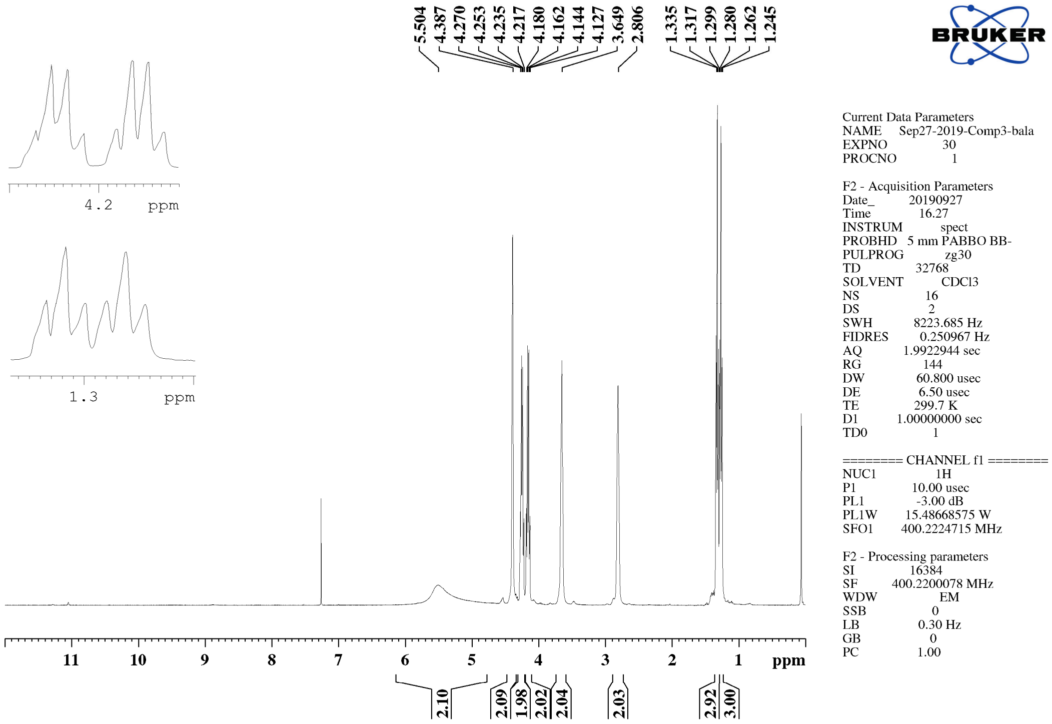

Figure **S47**. ^{1}H NMR of **3**

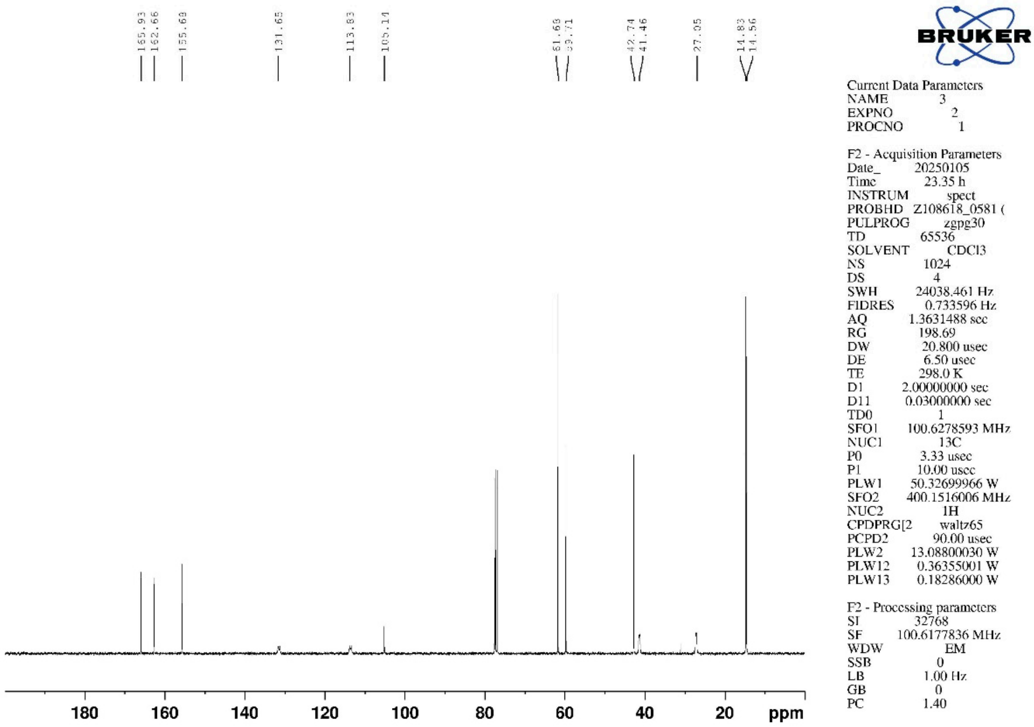

Figure **S48**. ^{13}C NMR of **3**

MTT assay results

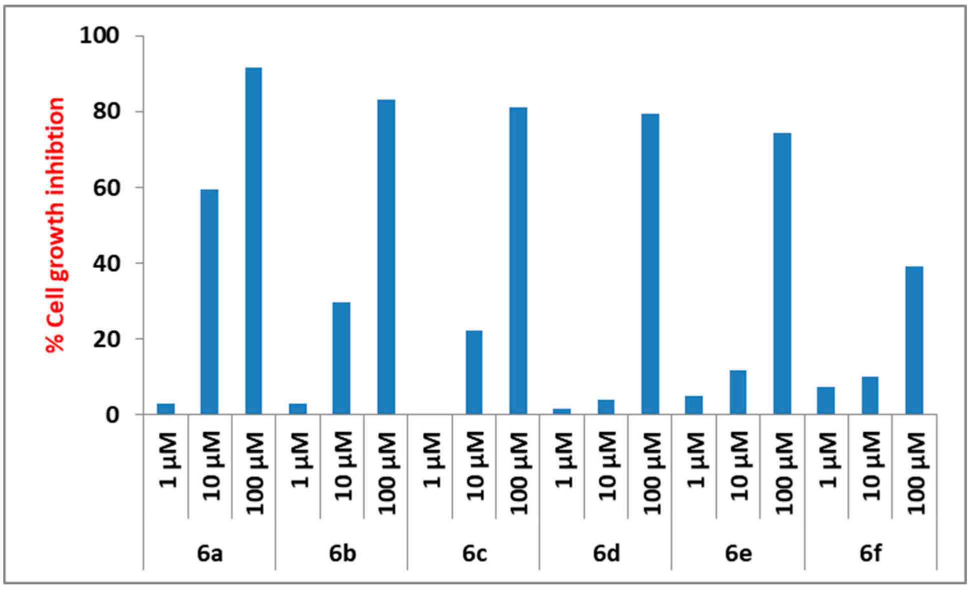

Figure **S49**. % Cell growth inhibition of the compounds **6(a-f)** against MCF7 cell line

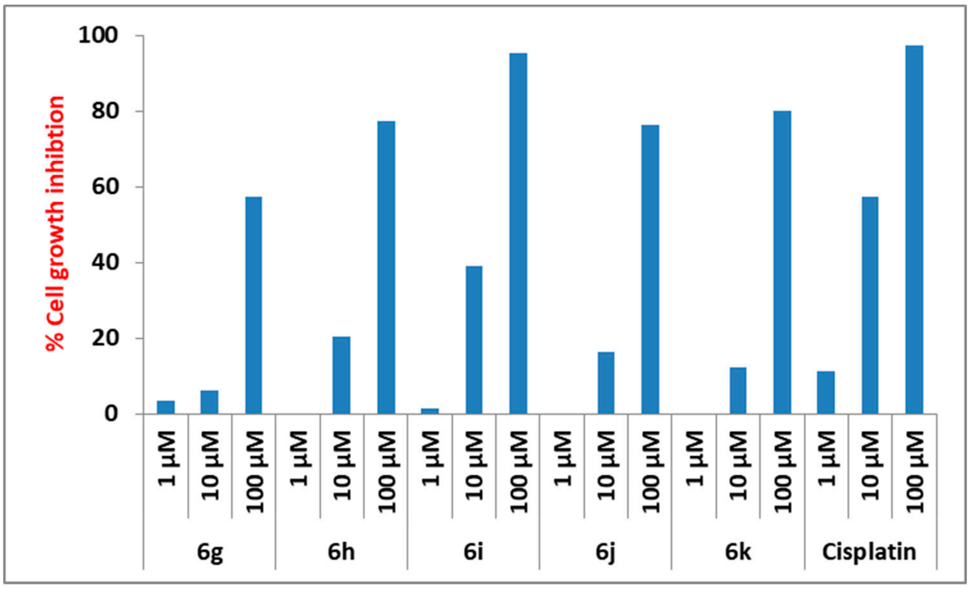

Figure **S50**. % Cell growth inhibition of the compounds **6(g-k)** and cisplatin against MCF7 cell line

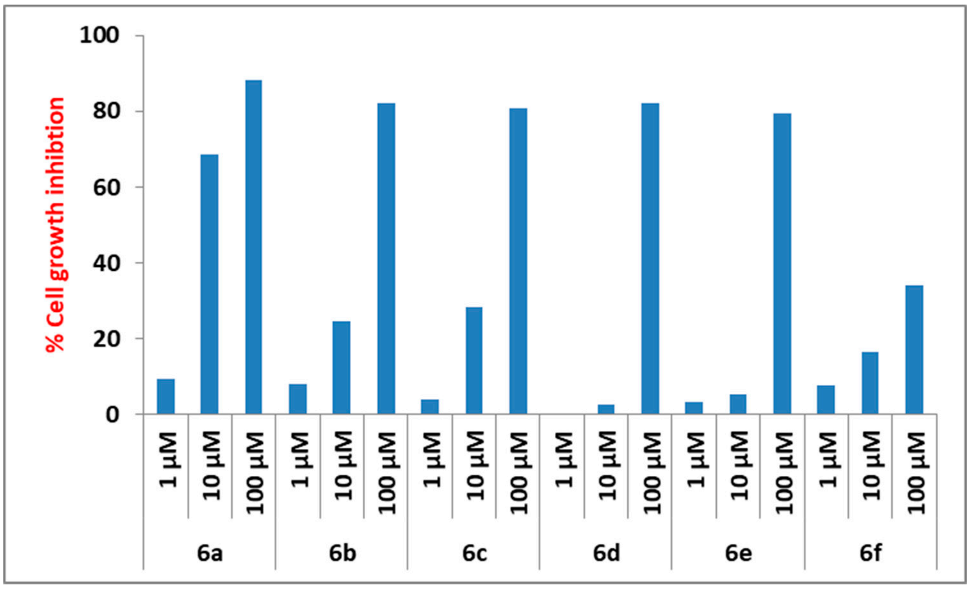

Figure **S51**. % Cell growth inhibition of the compounds **6(a-f)** against T47D cell line

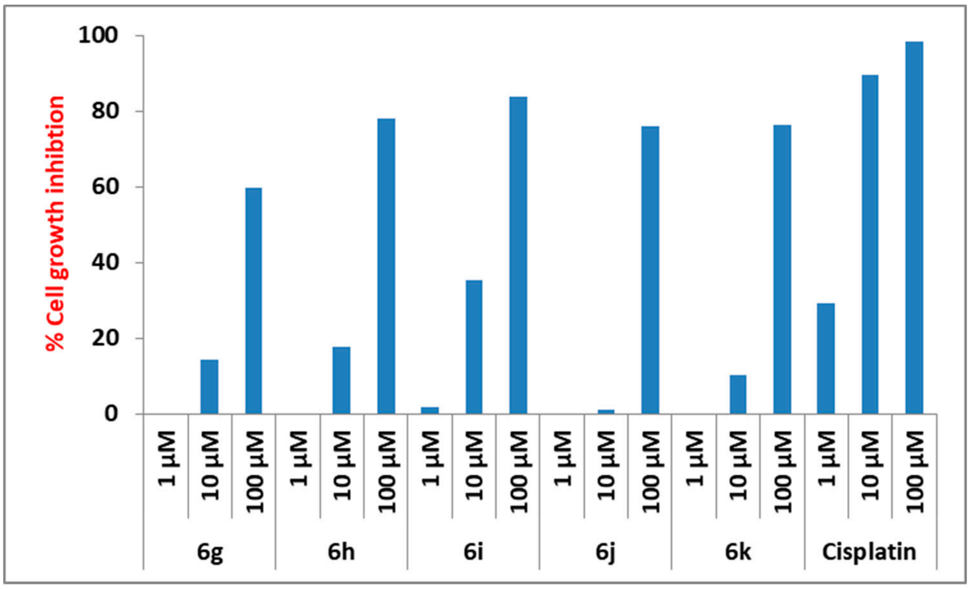

Figure **S52**. % Cell growth inhibition of the compounds **6(g-k)** and cisplatin against T47D cell line

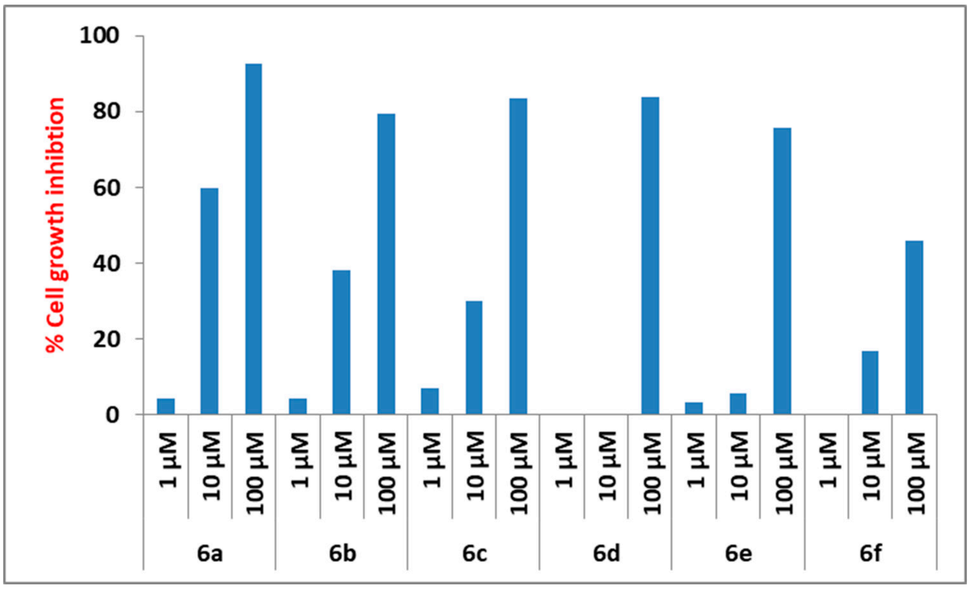

Figure **S53**. % Cell growth inhibition of the compounds **6(a-f)** against HSC3 cell line

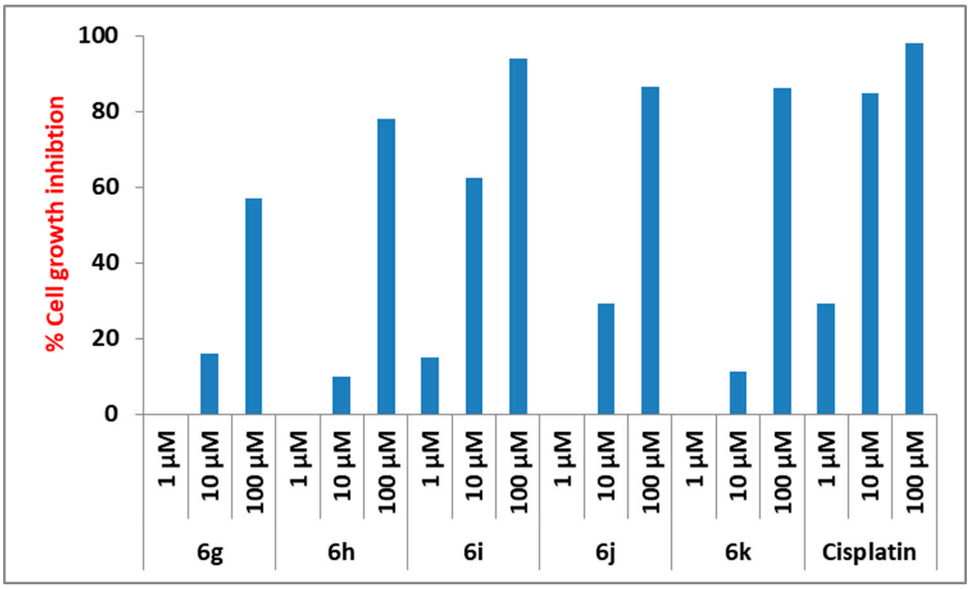

Figure **S54**. % Cell growth inhibition of the compounds **6(g-k)** and cisplatin against HSC3 cell line

Docking pose of ZZ6

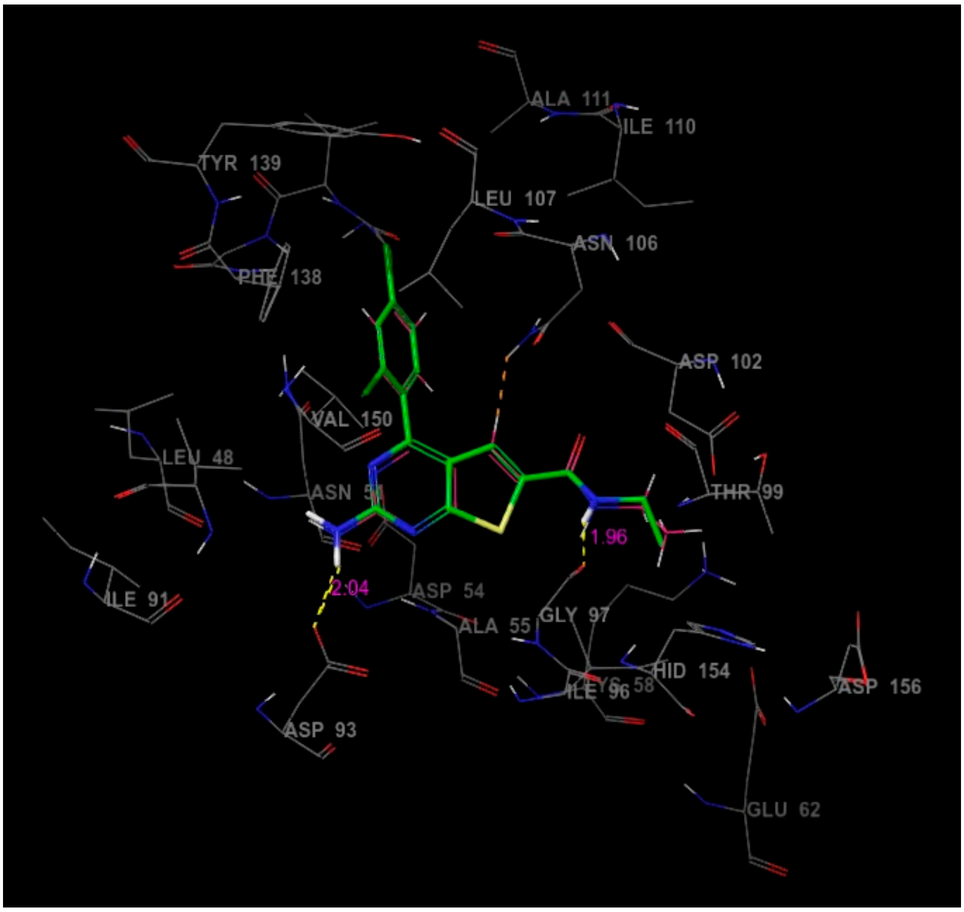

Figure **S55**. The docked ZZ6 (thick tube model; green-colored carbons) and the original ZZ6 (thin tube model; pink-colored carbons) poses. The yellow dashed line indicates the hydrogen bond interactions between the ligand and the binding site of Hsp90.

Docking poses of ligands

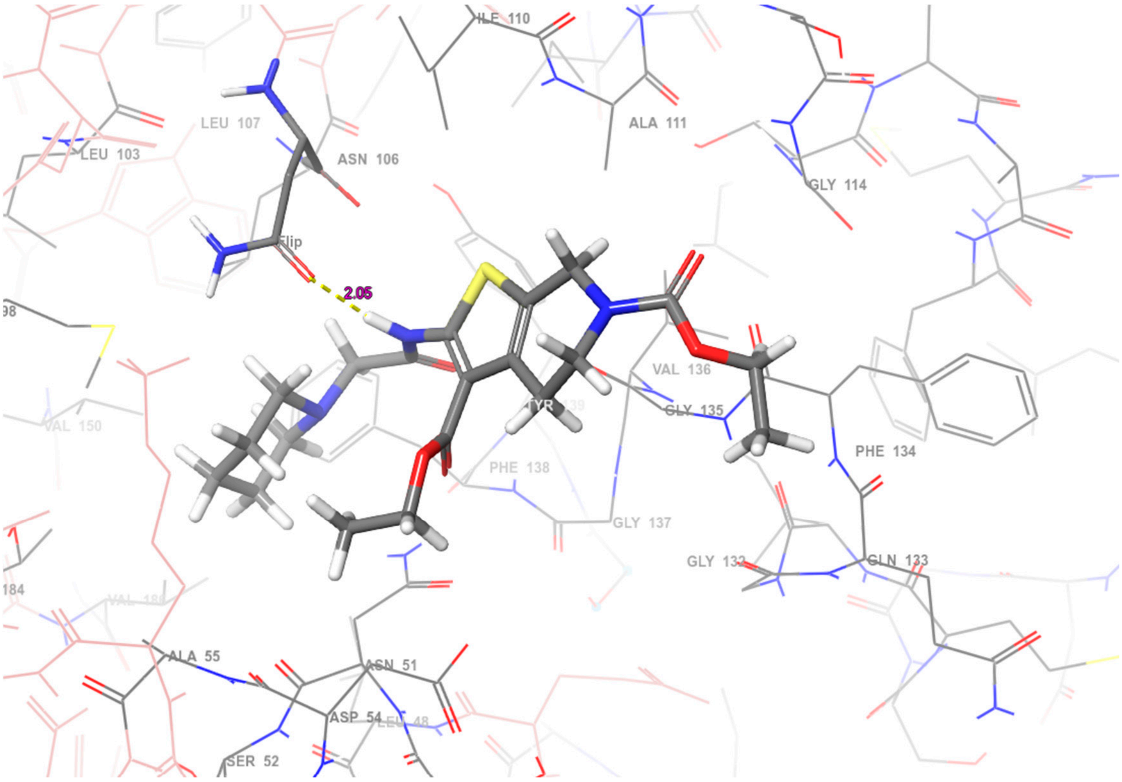

a

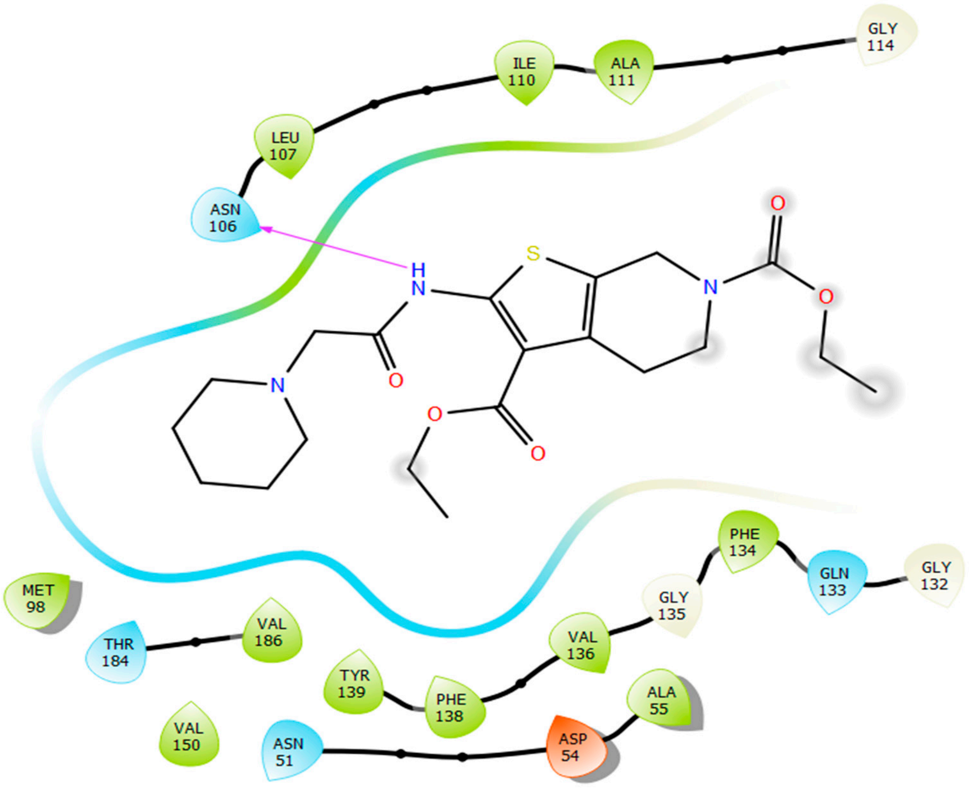

b

Figure **S56**. Molecular interactions of **6a** in 3D (**a**) and 2D (**b**) representations in alignment with the binding-site residues.

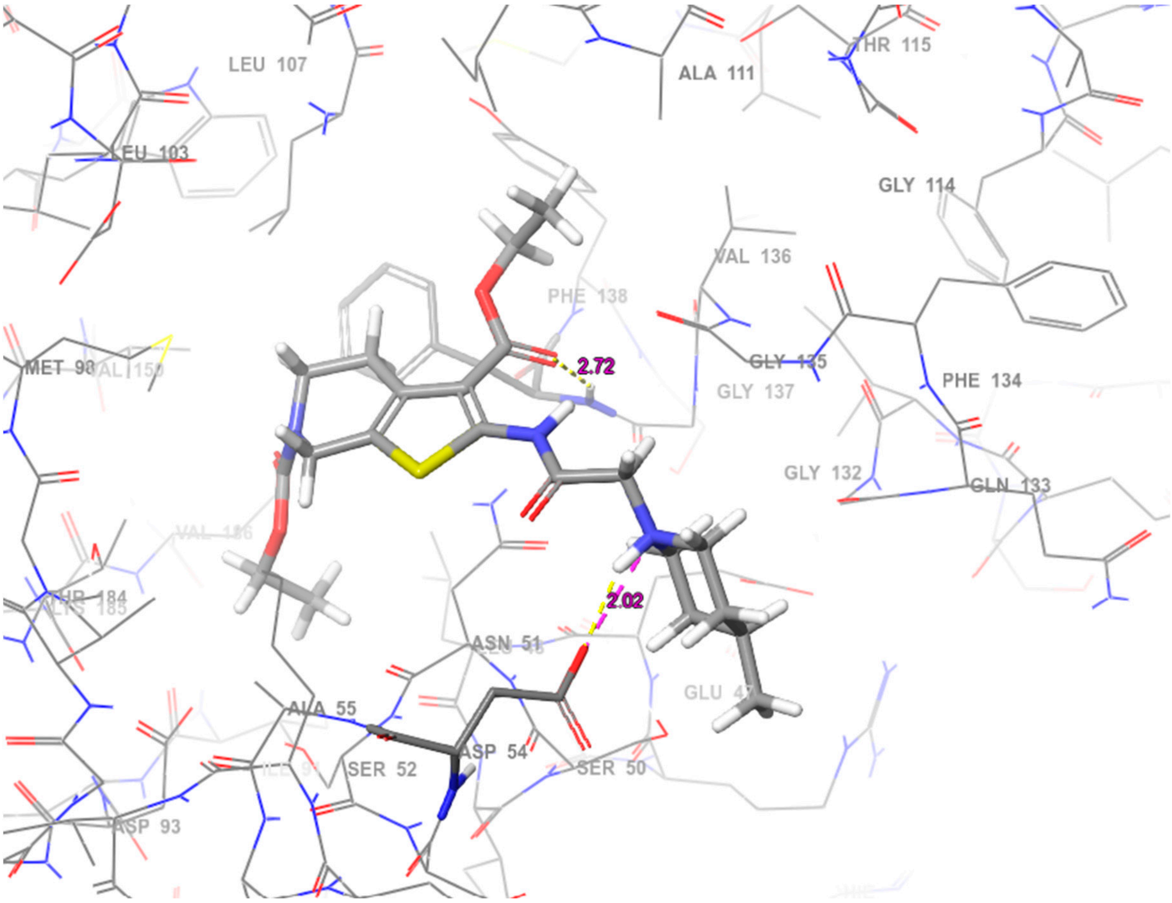

a

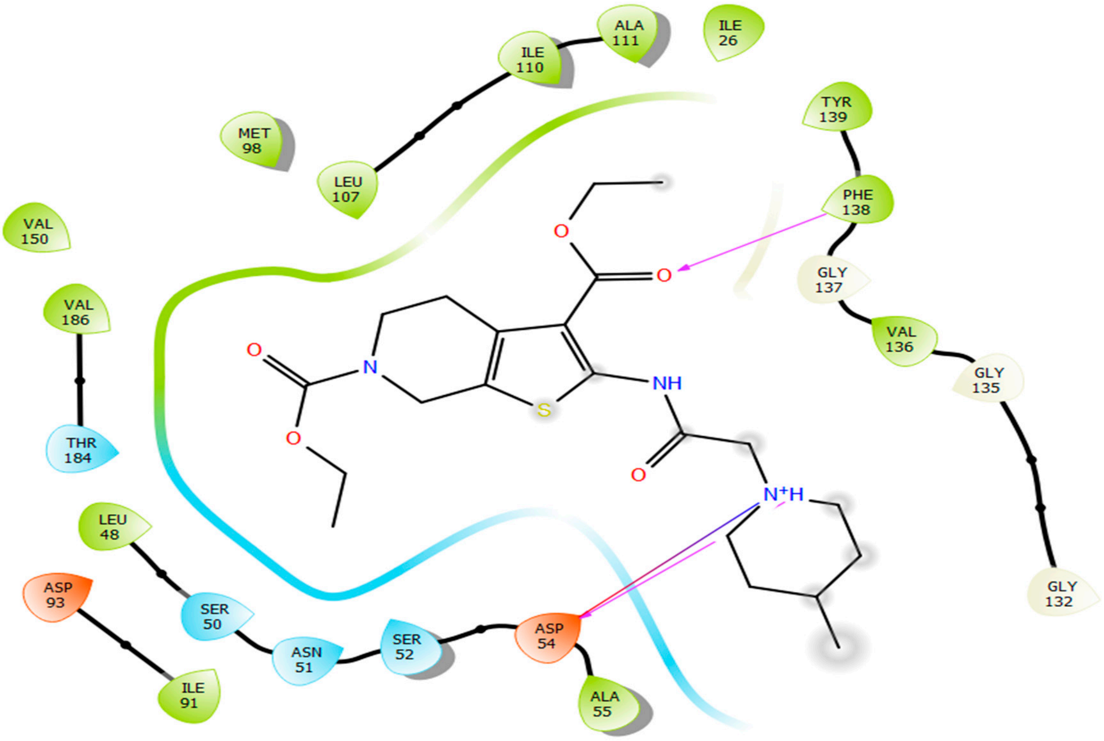

b

Figure **S57**. Molecular interactions of **6b** in 3D (**a**) and 2D (**b**) representations in alignment with the binding-site residues.

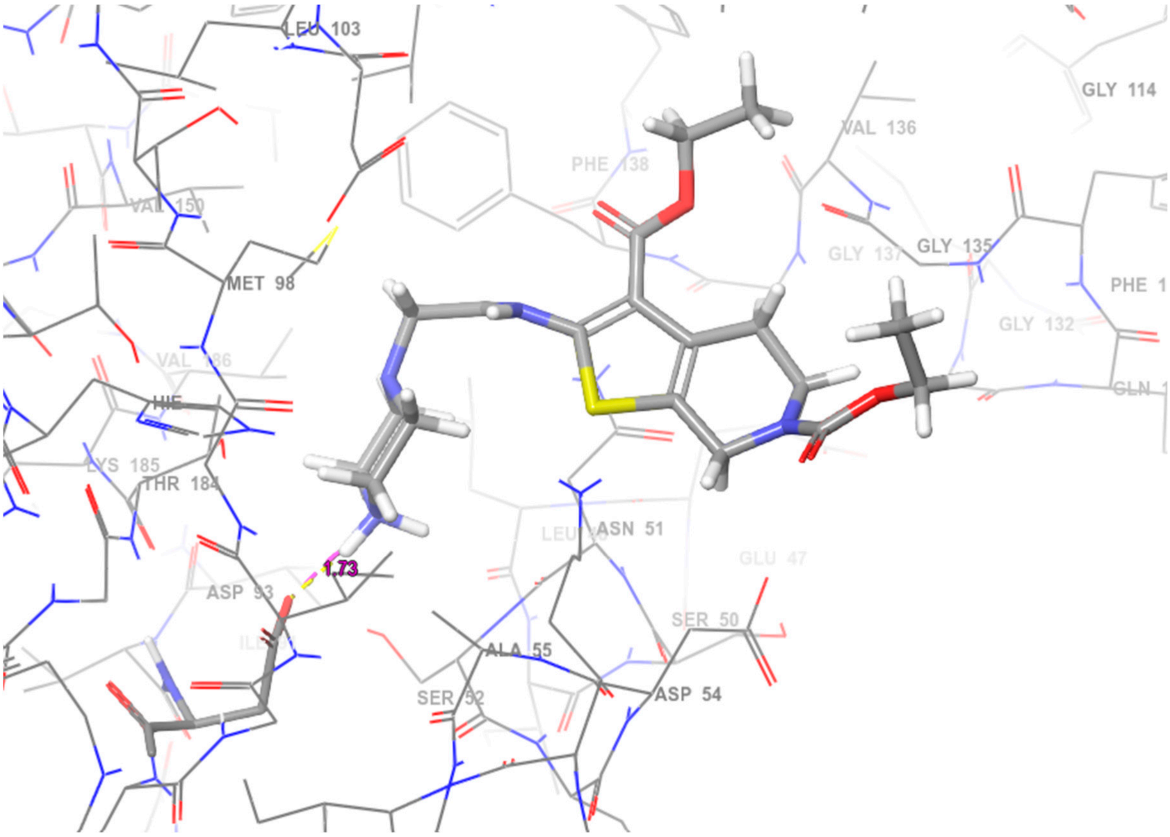

a

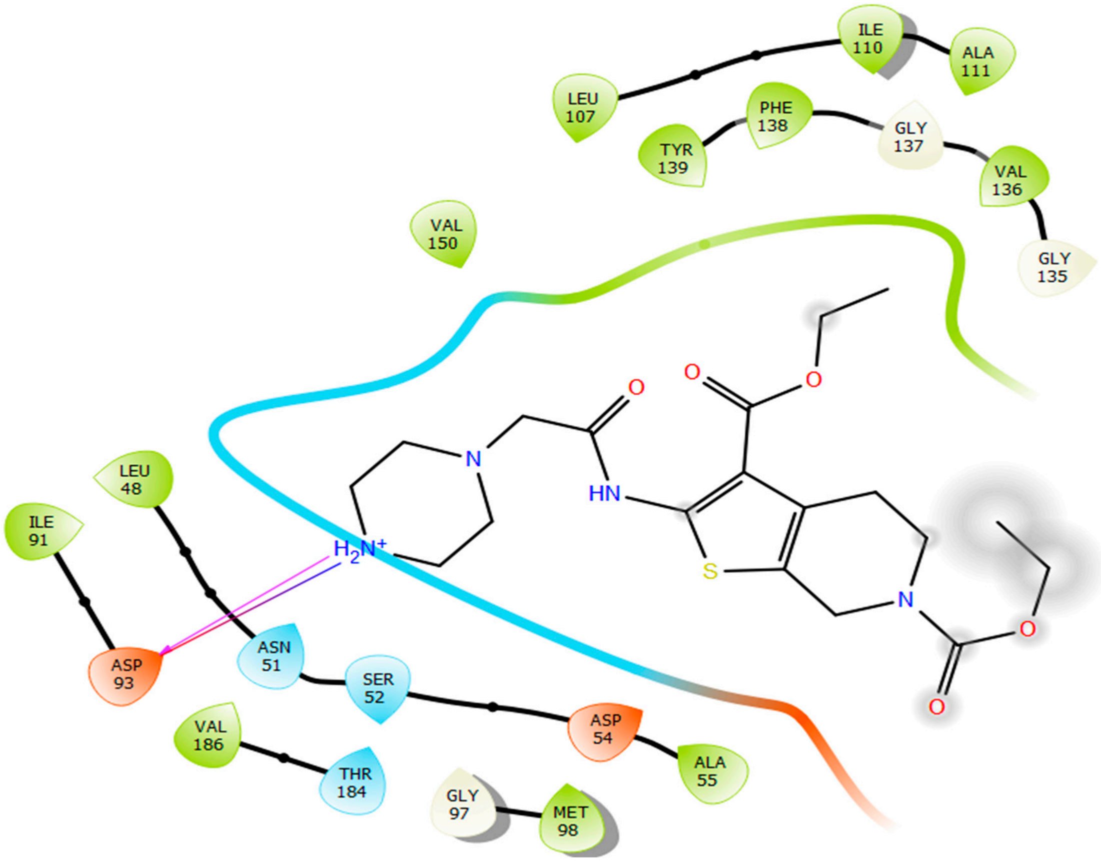

b

Figure **S58**. Molecular interactions of **6c** in 3D (**a**) and 2D (**b**) representations in alignment with the binding-site residues.

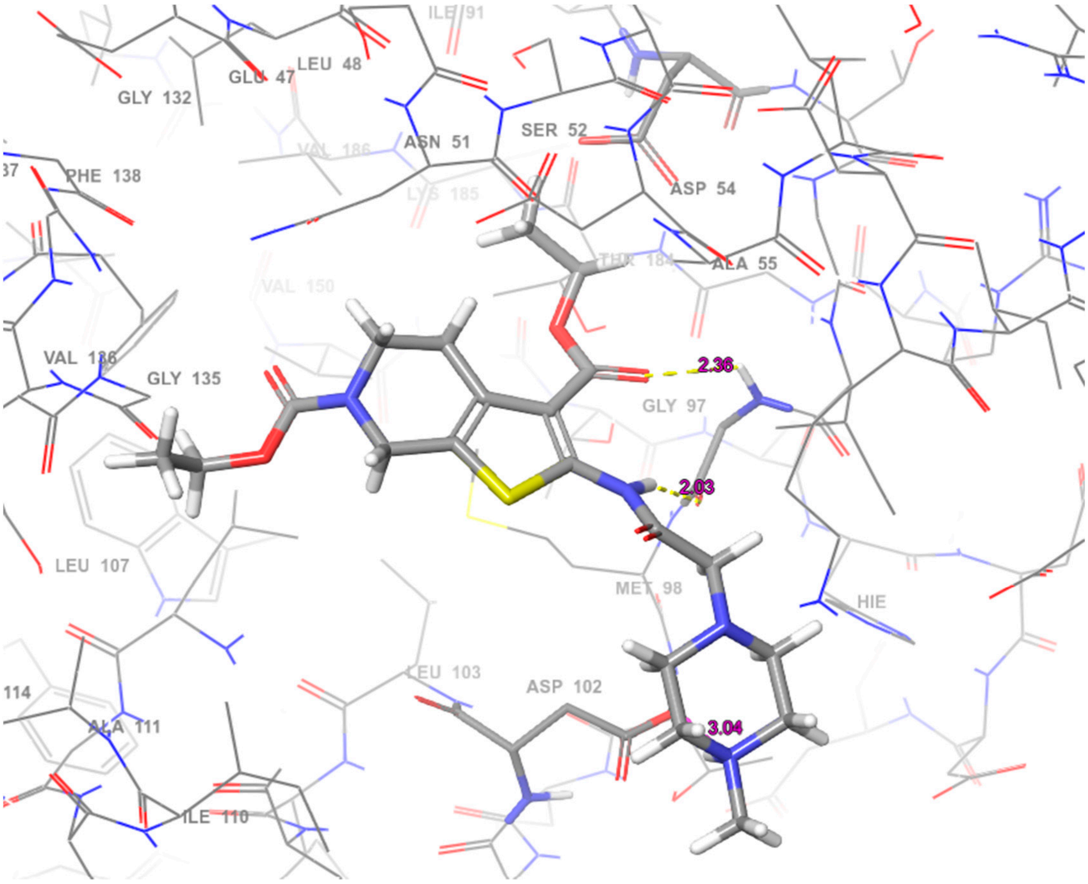

a

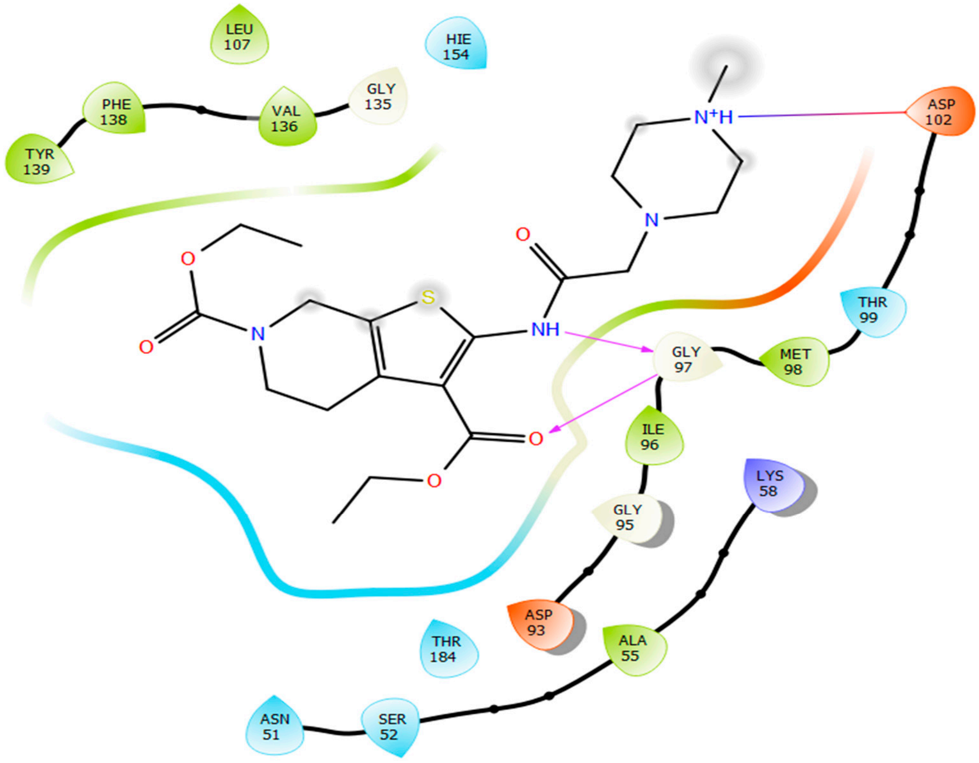

b

Figure **S59**. Molecular interactions of **6d** in 3D (**a**) and 2D (**b**) representations in alignment with the binding-site residues.

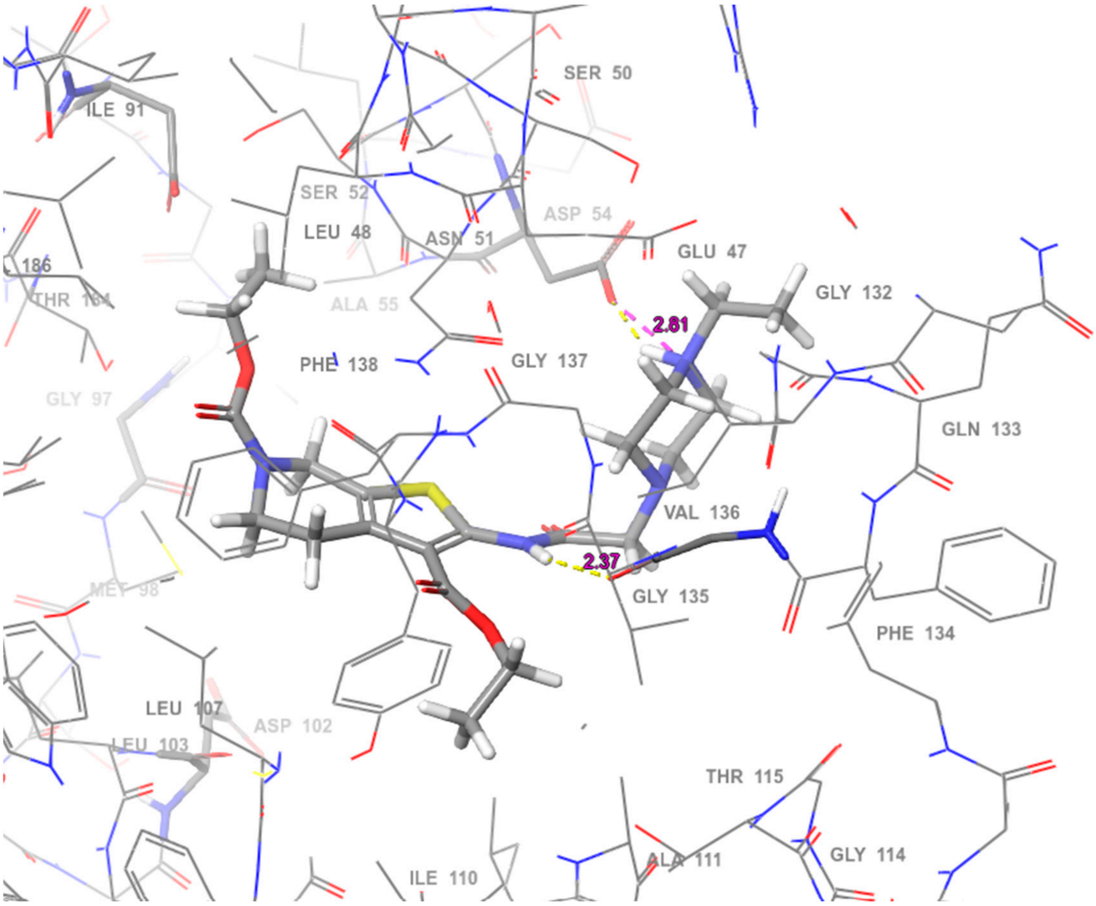

a

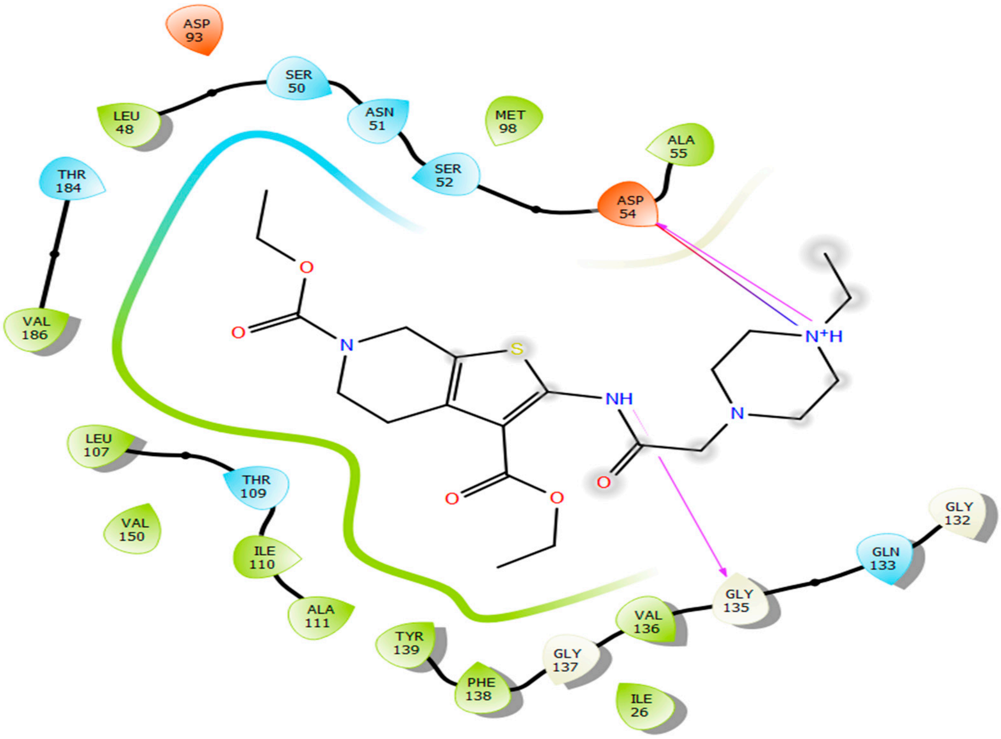

b

Figure **S60**. Molecular interactions of **6e** in 3D (**a**) and 2D (**b**) representations in alignment with the binding-site residues.

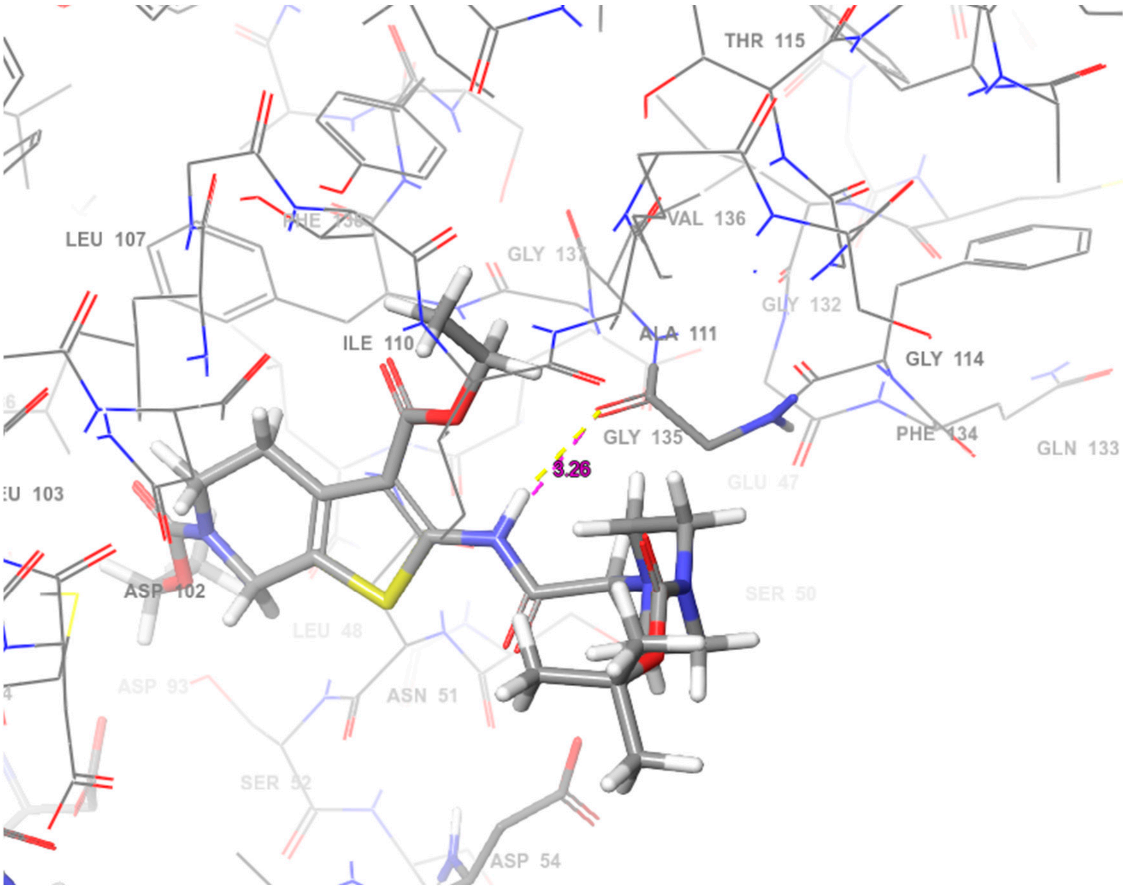

a

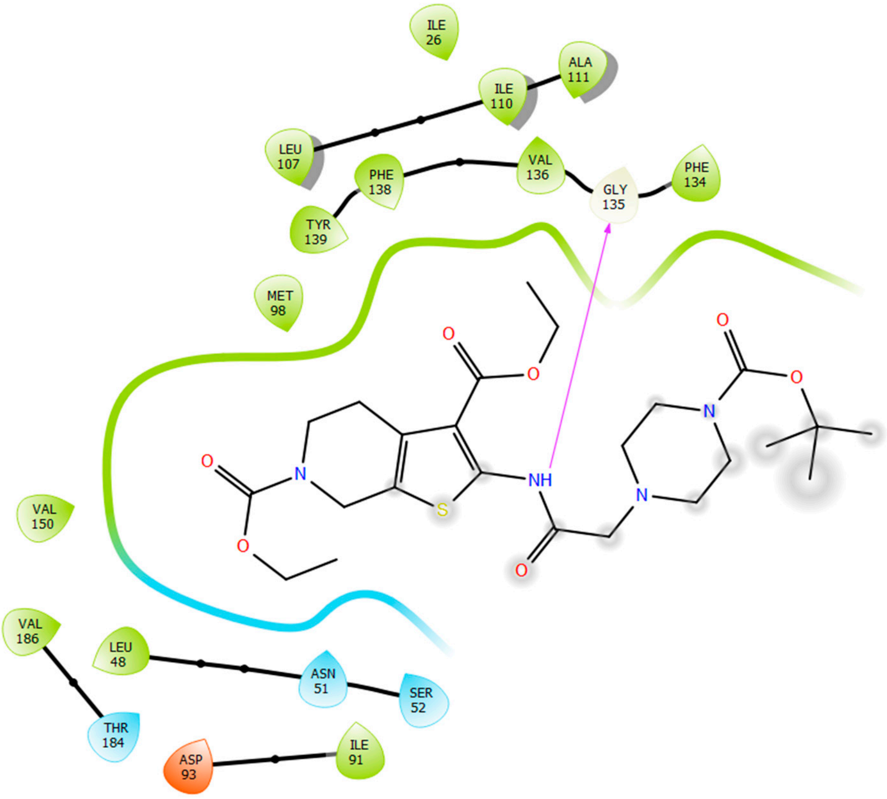

b

Figure **S61**. Molecular interactions of **6f** in 3D (**a**) and 2D (**b**) representations in alignment with the binding-site residues.

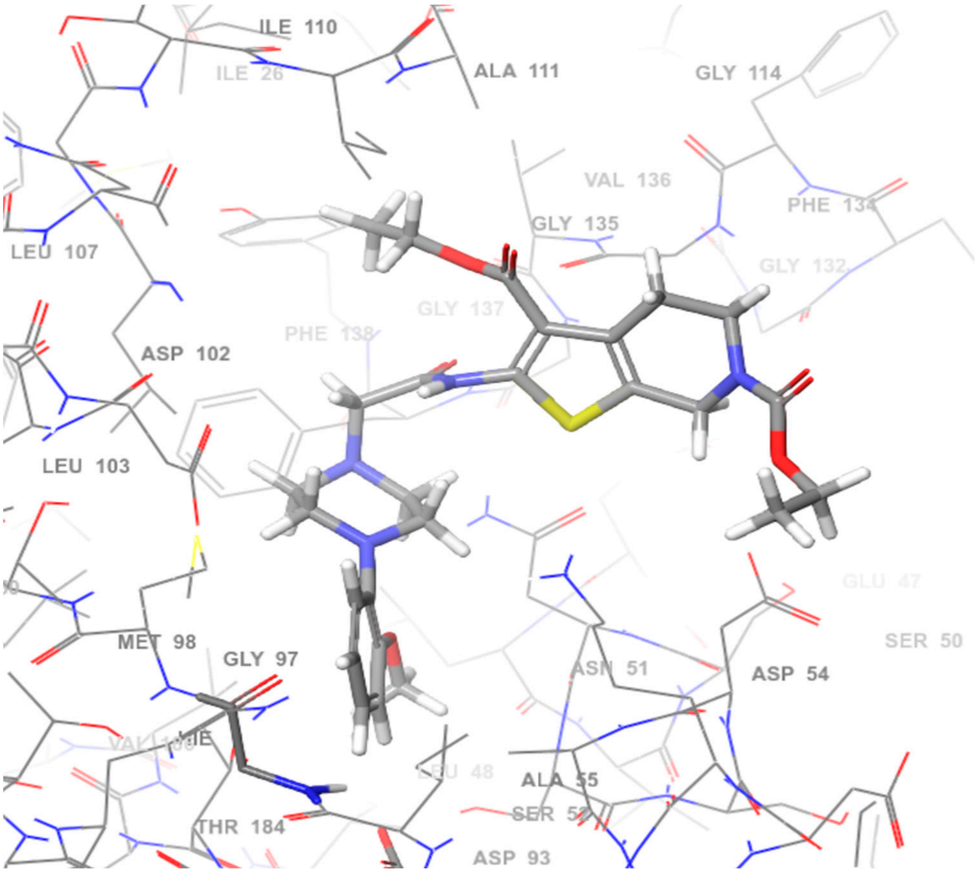

a

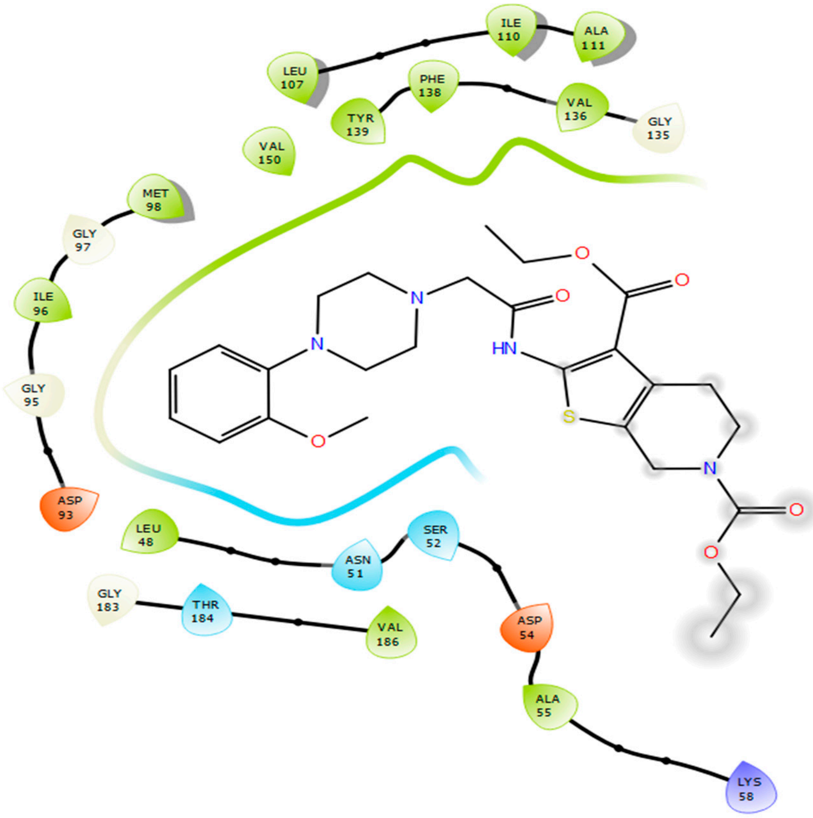

b

Figure **S62**. Molecular interactions of **6g** in 3D (**a**) and 2D (**b**) representations in alignment with the binding-site residues.

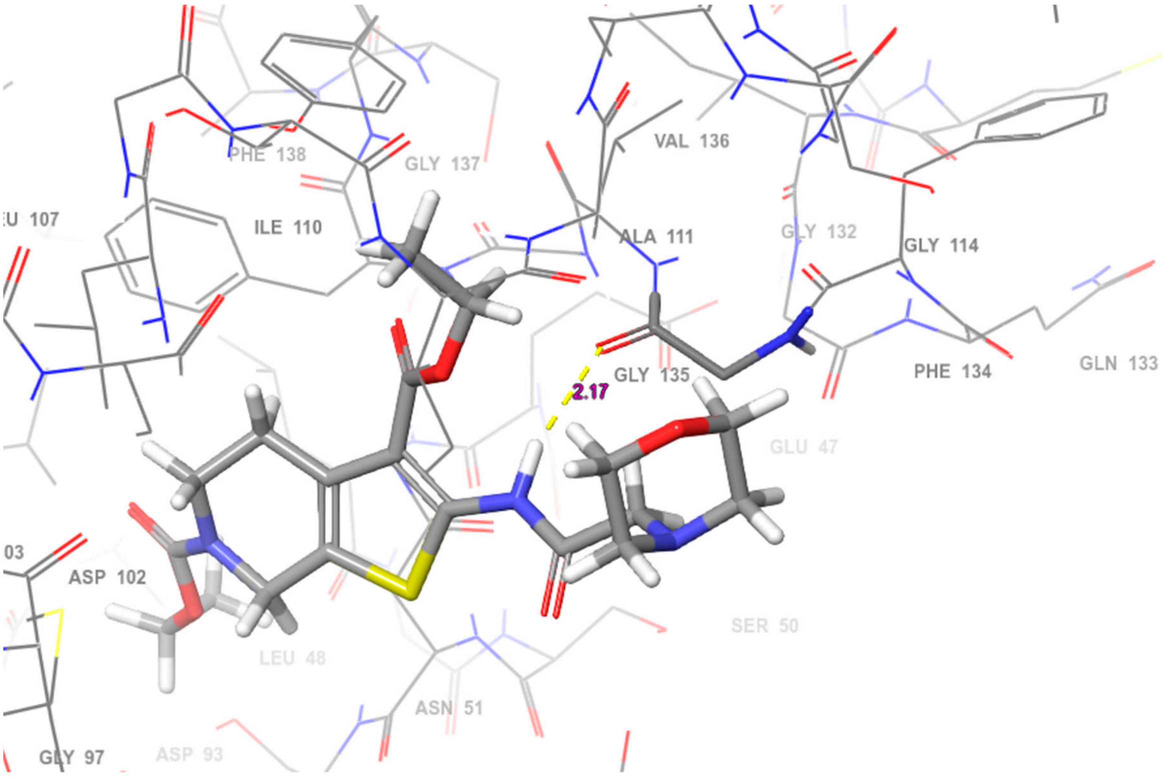

a

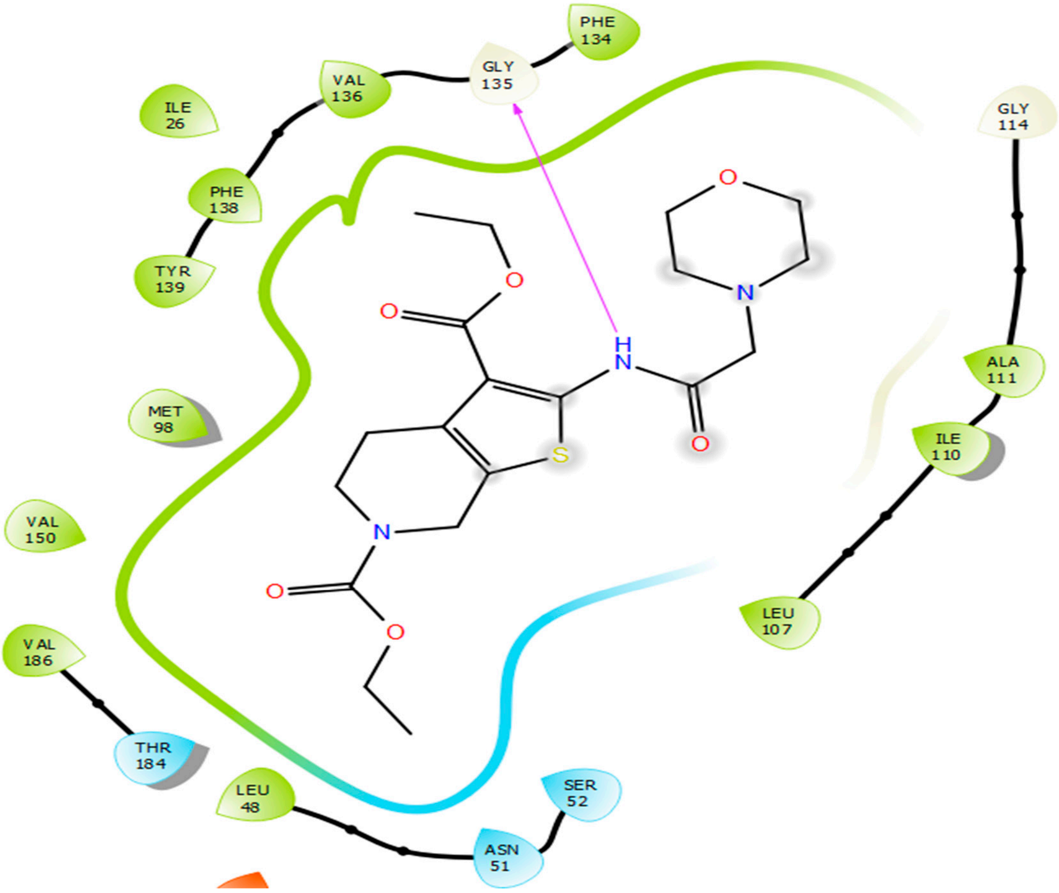

b

Figure **S63**. Molecular interactions of **6h** in 3D (**a**) and 2D (**b**) representations in alignment with the binding-site residues.

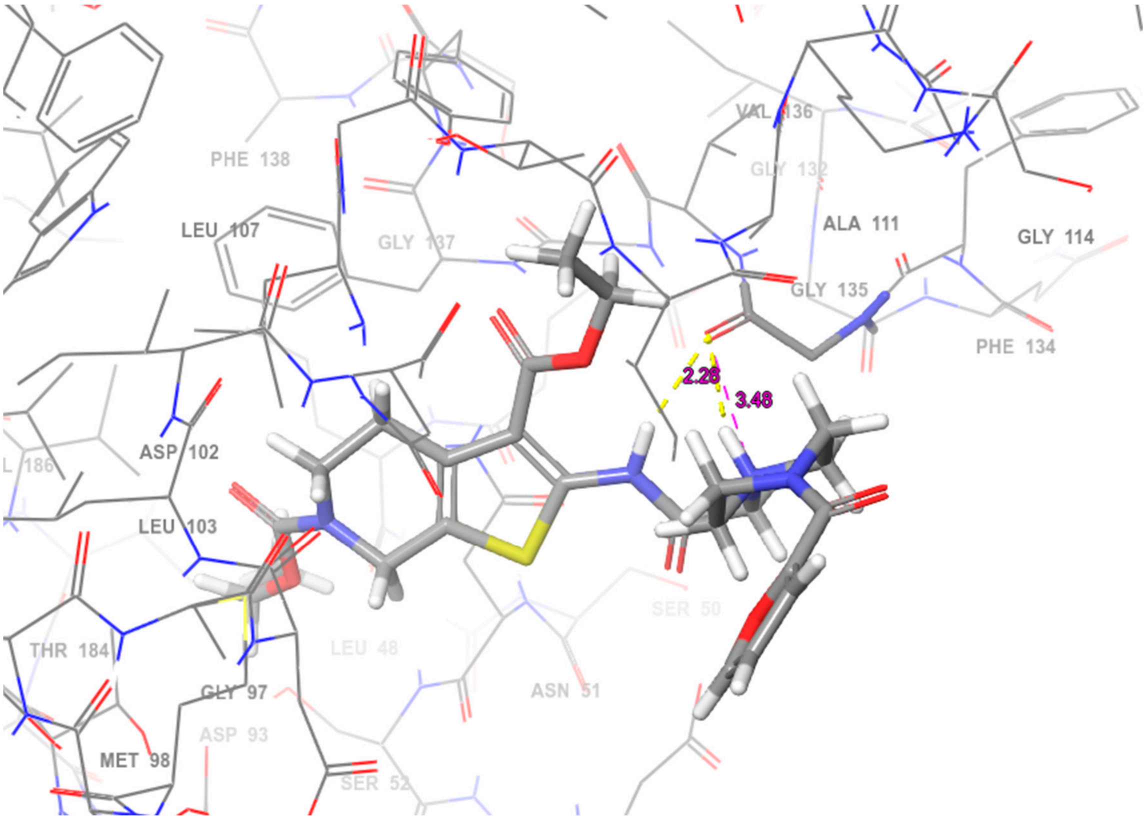

a

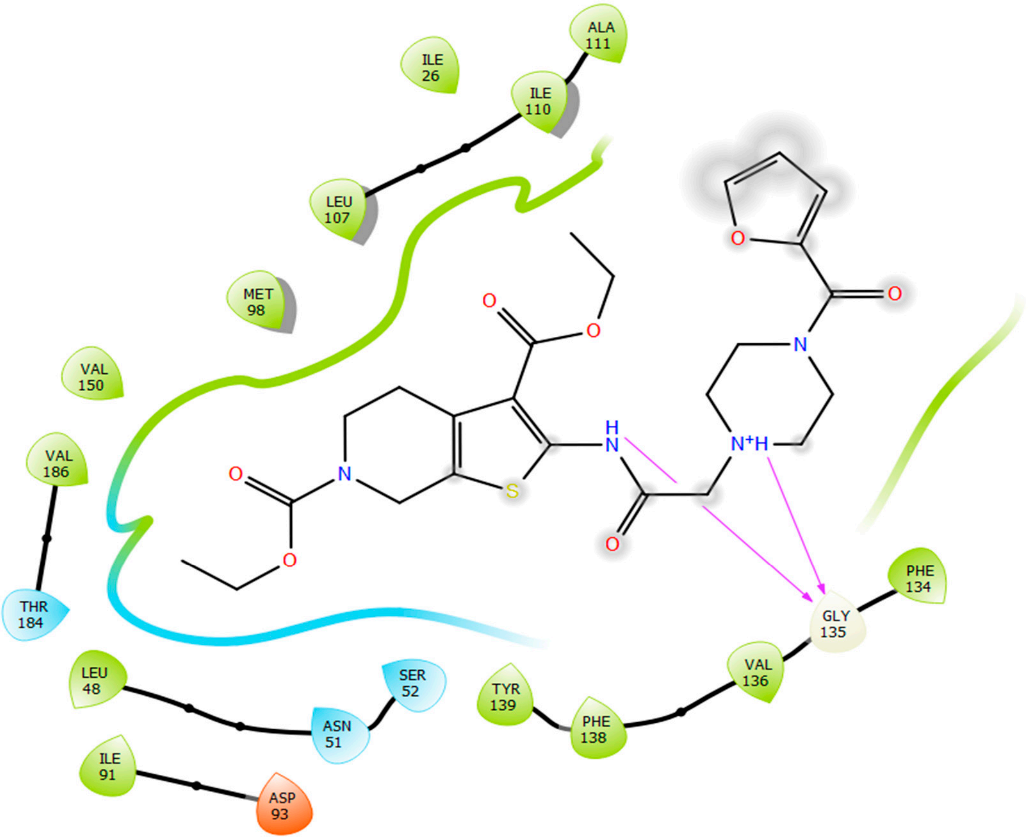

b

Figure **S64**. Molecular interactions of **6j** in 3D (**a**) and 2D (**b**) representations in alignment with the binding-site residues.

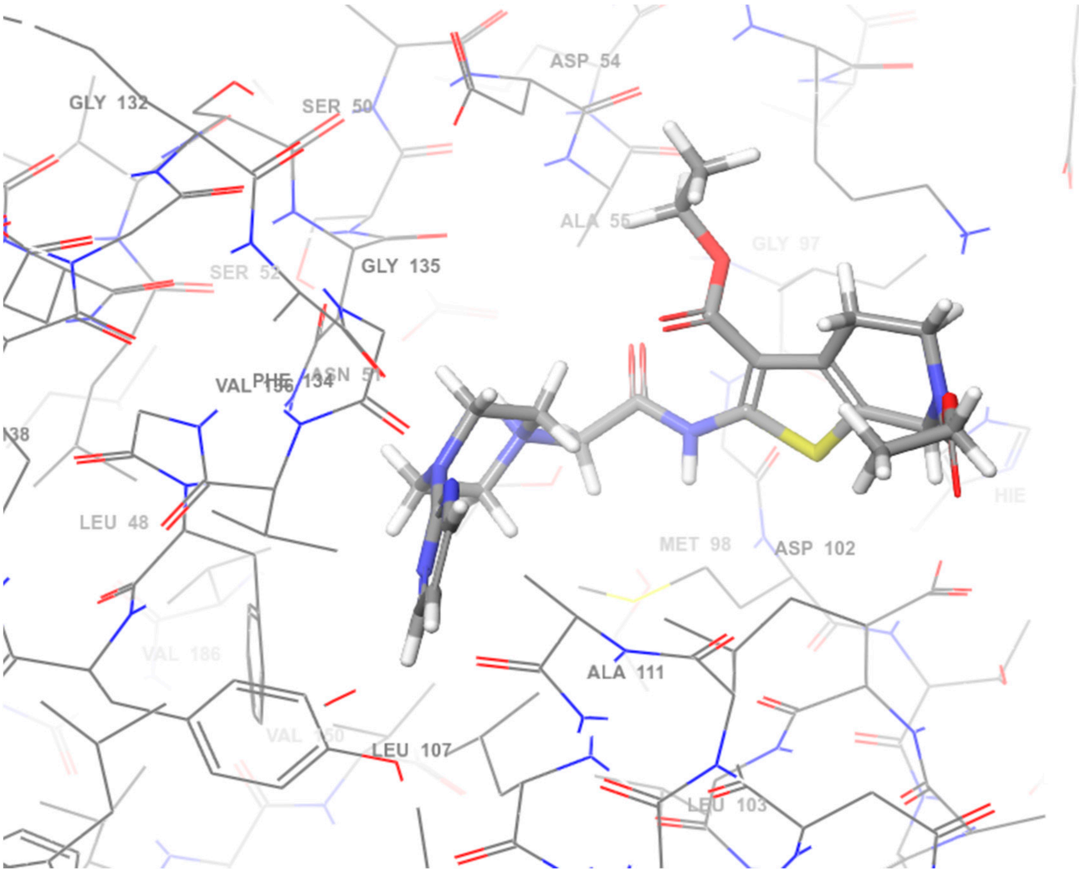

a

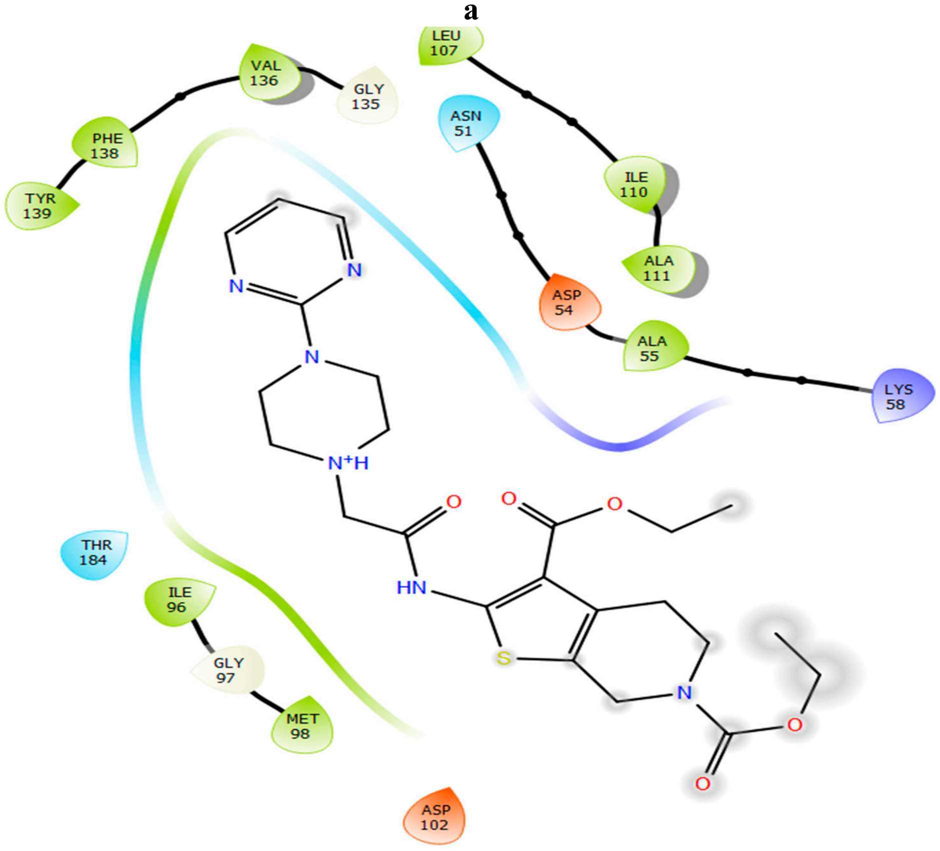

b

Figure **S65**. Molecular interactions of **6k** in 3D (**a**) and 2D (**b**) representations in alignment with the binding-site residues.
